# Molecular docking studies of coumarin hybrids as potential acetylcholinesterase, butyrylcholinesterase, monoamine oxidase A/B and β-amyloid inhibitors for Alzheimer’s disease

**DOI:** 10.1186/s13065-018-0497-z

**Published:** 2018-12-04

**Authors:** Samina Khan Yusufzai, Mohammad Shaheen Khan, Othman Sulaiman, Hasnah Osman, Dalily Nabilah Lamjin

**Affiliations:** 1 School of Industrial Technology, Universiti Sains Malaysia, 11800 Penang, Malaysia; 2 Industrial Chemistry Programme, Faculty of Science and Natural Resources, Universiti Malaysia Sabah, 88400 Kota Kinabalu, Sabah Malaysia; 3 School of Chemical Sciences, Universiti Sains Malaysia, 11800 Penang, Malaysia

**Keywords:** Coumarin, Neurodegenerative disorder, Alzheimer’s disease, Acetylcholinesterase, Butyrylcholinesterase, Monoamine oxidase

## Abstract

Coumarins are the phytochemicals, which belong to the family of benzopyrone, that display interesting pharmacological properties. Several natural, synthetic and semisynthetic coumarin derivatives have been discovered in decades for their applicability as lead structures as drugs. Coumarin based conjugates have been described as potential AChE, BuChE, MAO and β-amyloid inhibitors. Therefore, the objective of this review is to focus on the construction of these pharmacologically important coumarin analogues with anti-Alzheimer’s activities, highlight their docking studies and structure–activity relationships based on their substitution pattern with respect to the selected positions on the chromen ring by emphasising on the research reports conducted in between year 1968 to 2017.
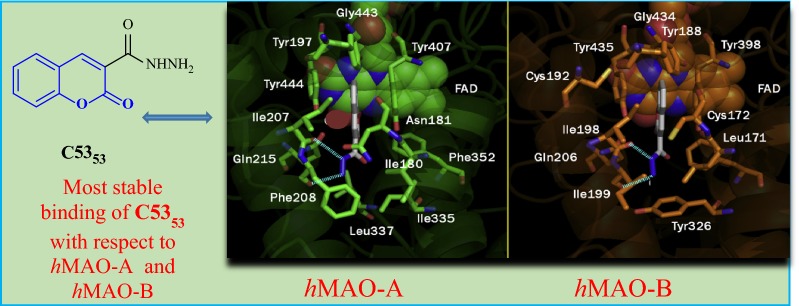

## Introduction

**Fig. 1:**
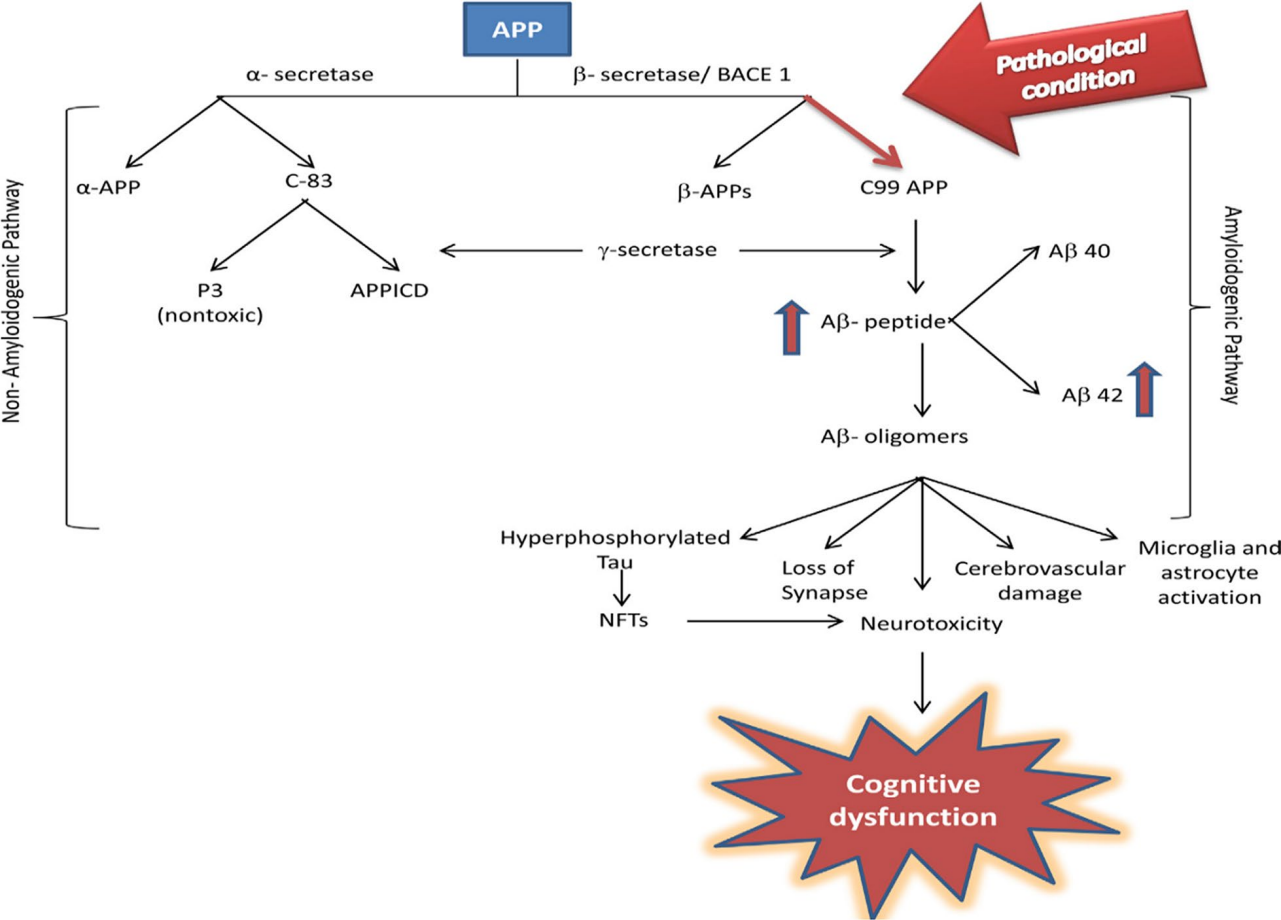
Diagrammatic presentation of APP processing pathways [1]

**Fig. 2:**
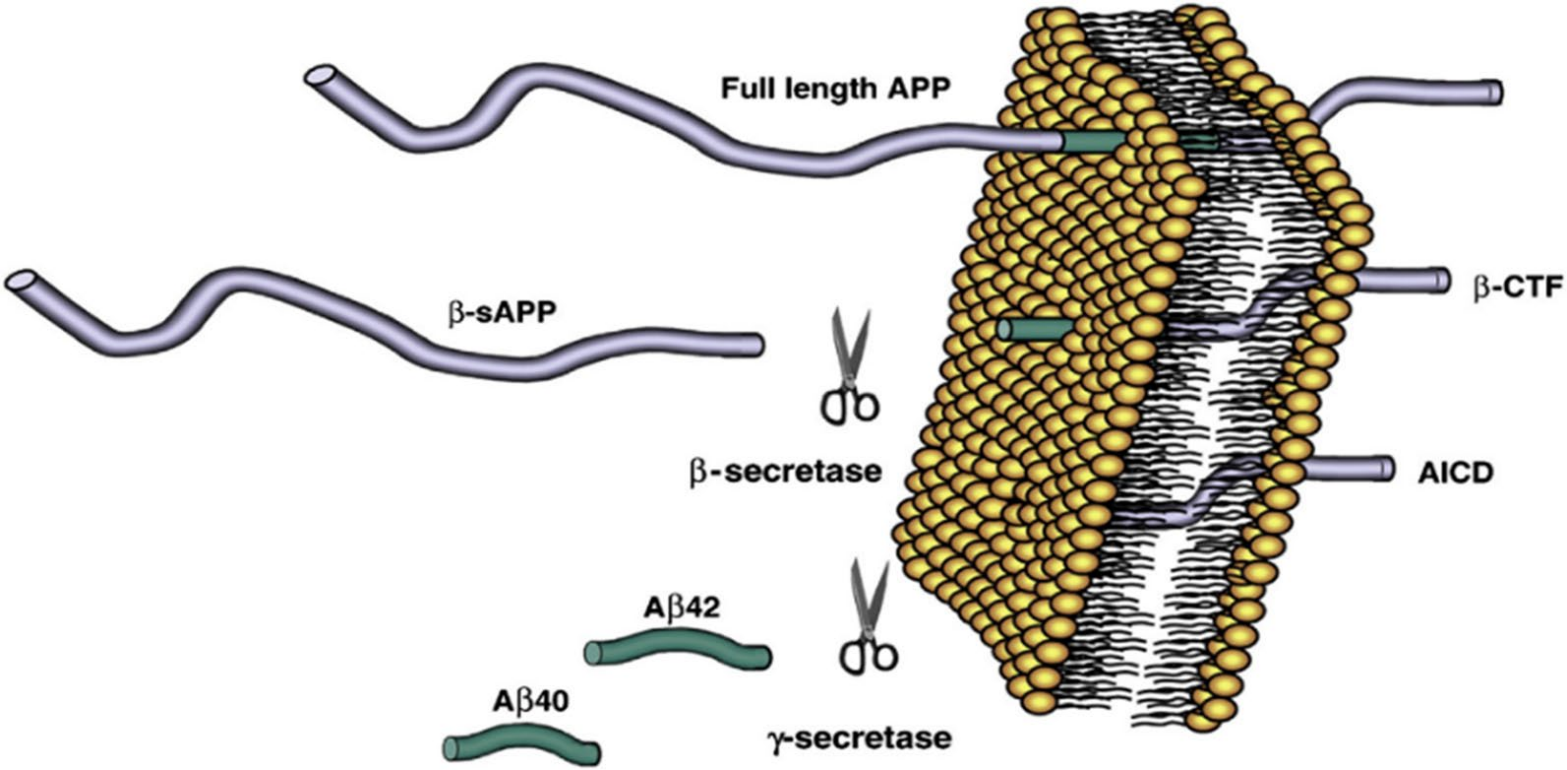
Generation of soluble Aβ fibrils from amyloid precursor protein [2]

Alzheimer’s disease (AD) is the most common form of neurodegenerative disorder and the most prevalent cause of dementia commonly affecting the elderly. It is a progressive disorder of the brain that is associated with the loss of presynaptic markers of the cholinergic system in the brain, which is related to memory and ability to carry out daily activities. It is said to be progressive as its symptoms worsen over time. Two main causes of AD are plaques and neurofibrillary tangles (NFTs) which results due to the accumulation of beta-amyloid protein (Aβ) outside the neurons. Aβ is formed by the proteolytic cleavage of amyloid precursor protein (APP) which occurs by α-secretase and is aberrantly processed by β- and γ- secretases resulting in an imbalance between production and clearance of Aβ peptide and thus Aβ forms highly insoluble and proteolysis resistant fibrils known as senile plaques (Fig. 1) [1]. These plaques will interrupt the neuron transmission at synapses and cause information transfer to fail leading to the neuronal cell death. NFTs are composed of the tau amyloid protein fibrils (Fig. 2) [2].

**Fig. 3:**
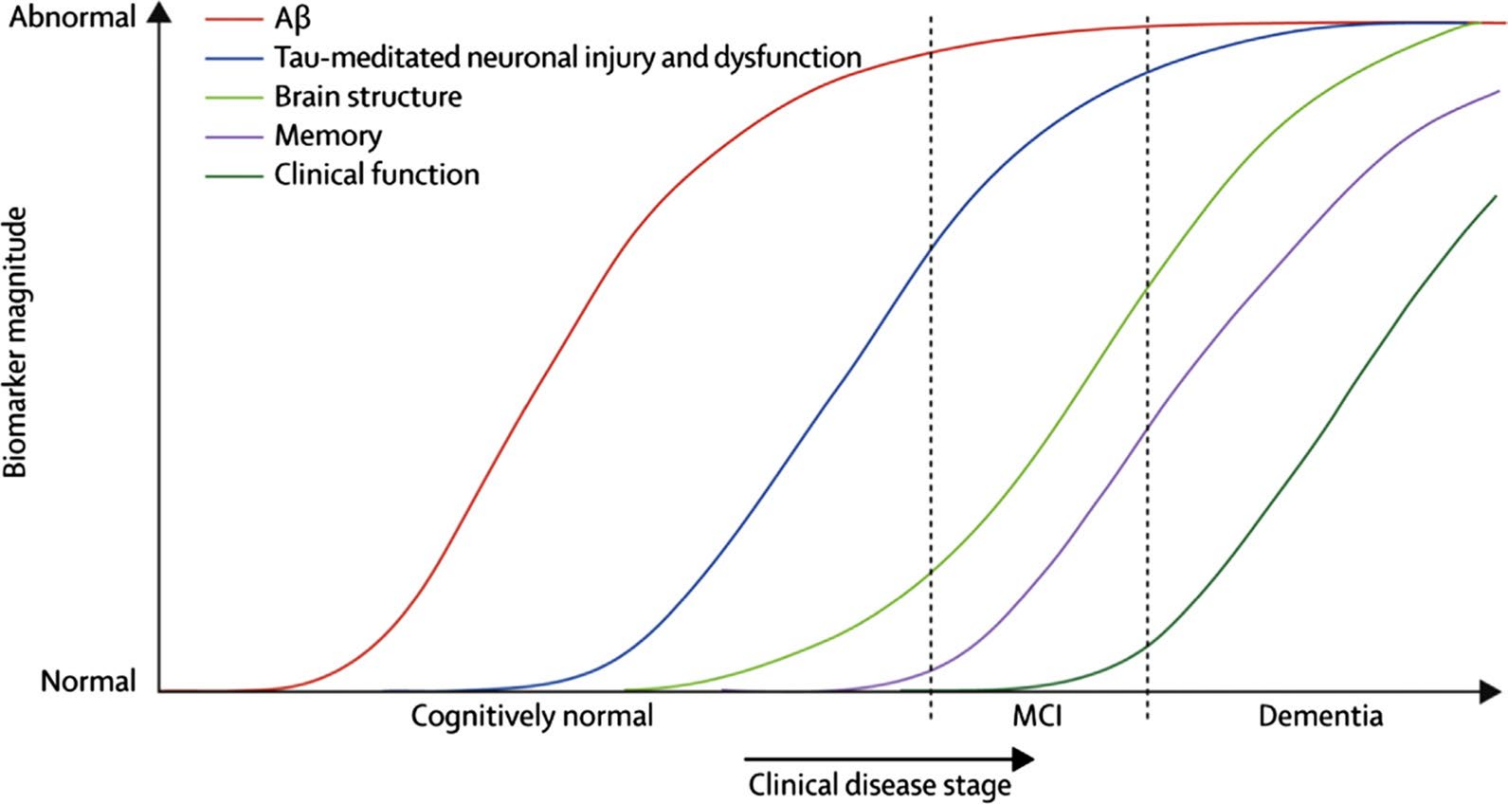
Hypothetical model for biomarker dynamics in the progression of Alzheimer’s disease [4]

Tau is a component of microtubules that provides the internal support structure for the transport of nutrients and essential molecules within the cell. When tau is hyperphosphorylated, it forms insoluble fibrils that blocks the transport of nutrients and essential molecules in the neuron thus leading to cell death [3]. Jack et al. proposed a hypothetical model which explains about the progression of AD and how pathological events such as deposition of Aβ fibrils and increased levels of tau protein in cerebrospinal fluid (CSF), lead to cognitive impairment and dementia (Fig. 3) [4]. To date, the cure of this disease is yet to discover. Nonetheless, researchers have found various alternatives to slow down its progression. Among these alternatives are, inhibition of acetylcholinesterase (AChE), APP, β-secretase, γ-secretase, monoamine oxidase (MAO) and metal chelators [5].

The first line treatment that is given to AD patients is AChE inhibitors because not only they facilitate cholinergic transmission, they also interfere with the synthesis, deposition and aggregation of toxic Aβ. This might lead to the improvement of cognition and some behavioural problems [6–8]. The enzyme butyrylcholinesterase (BuChE) has the same role as AChE, which is to hydrolyse the acetylcholine in the synaptic cleft. However, their inhibition might help in enhancing the efficiency of treatment for the AD patients. Xie et al. stated that even though the activity of AChE decreases as the disease progresses, the activity of BuChE shows a significant increase in the hippocampus and temporal cortex. BuChE inhibitors might help to improve cholinergic activity by restoring the AChE/BuChE activity ratios as seen in the healthy brain [9]. Recent investigations are focusing more on dual AChE/BuChE inhibitors [8–10]. Monoamine oxidase B (MAO-B) is an important factor that is involved in oxidative stress and oxidative stress is said to be among the multiple factors, which induce the AD. It is widely established in the literature that the activity of MAO-B can increase up to threefold in the temporal, parietal and frontal cortex of AD patients as compared to the controls. This increase in MAO-B activity produces higher levels of H_2_O_2_ and oxidative free radicals, which has been correlated, with the development of Aβ plaques. MAO-B inhibitors, hold the potential to be developed into effective anti-Alzheimer’s drugs, as it has been reported before, that MAO-B inhibitors such as selegiline and rasagiline has shown to significantly improve the learning and memory deficits in the animal models, associated with AD and to slow the disease progression in AD patients [11–13]. Zatta et al. reported that dyshomeostasis and miscompartmentalization of metal ions such as Fe^2+^, Cu^2+^ and Zn^2+^ occurs in the brain of AD patients. The formation of Aβ plaques, neurofibrillary tangle as well as production of reactive oxygen species (ROS) and oxidative stress are closely linked to the highly concentrated metal ions in the neuropil and plaques of the brain [14]. Modulation of such biometals in the brain represents an additional rational approach for the treatment of AD [12, 14]. Another approach that gained interest among the researchers was to lower the Aβ level by inhibiting the β-secretase (BACE1), which is a transmembrane aspartyl protease, responsible for N-terminal cleavage of the APP which leads to the production of Aβ peptide [15].

Coumarin and its derivatives are reported to display wide range of biological activities such as anti-diabetic and antidepressant [16], anti-oxidant [17], anti-cancer [18], anti-proliferative [19], antinociceptive [20], anti-bacterial and anti-tubercular [21], hepatoprotective, anti-allergic, anti-HIV-1, antiviral, antifungal, antimicrobial and antiasthmatic [22]. The benzopyrone moiety of the coumarin nucleus is known as the fundamental for the design of hybrid molecule that can simultaneously inhibit AChE and AChE induced β-amyloid accumulation. Studies have also shown that naturally occurring as well synthetic coumarin analogues exhibit potent AChE, BuChE, dual AChE/BuChE and MAO inhibitory activity [12, 23–25]. Coumarin’s versatility allows chemical substitutions to occur at different sites in its structure, thus making it a compelling molecule for drug discovery [7, 8]. Modification of coumarin ring to develop new analogues of coumarin with superior activity is the main focus of the current review which is based on the reports which were taken in between the year 1968–2017.

## Coumarin analogues as AChE inhibitors

**Fig. 4:**
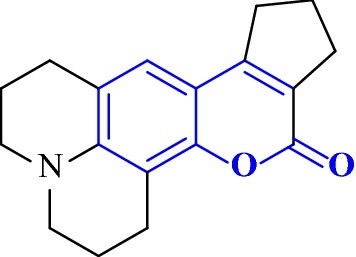
Molecular structure of coumarin 106 or **C1**

Fallarero et al. [7] reported an active AChE inhibitor among a coumarin library consisting of 29 coumarins, including coumarin itself and several derivatives of the 7-hydroxy and 7-methoxy-coumarin as well as seven synthetic coumarins. The molecule that showed most active AChE inhibitory activity is **C1**, chemically known as 2,3,5,6,7,9,10,11-octahydrocyclopenta[4,5]pyrano[2,3-f]pyrido[3,2,1-ij]quinolin-12(1H)-one or coumarin 106 (Fig. 4).

**Fig. 5:**
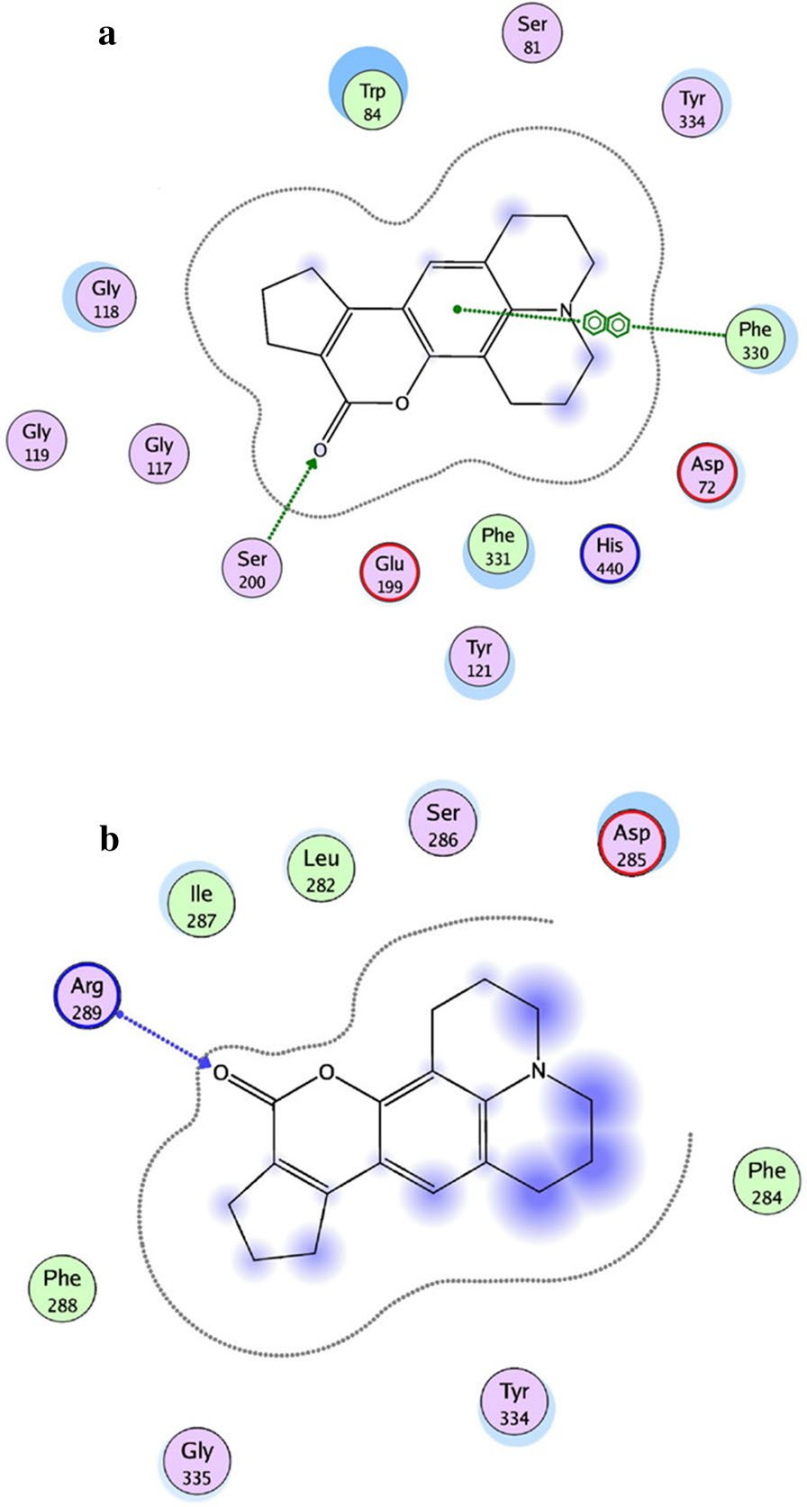
**a** Propose binding of **C1** at the active gorge site. **b** Propose binding of **C1** at the peripheral anionic site [7]

In order to recognize how this compound act as AChE inhibitor and interacts with the target, Fallarero et al. [7] docked **C1** into the enzyme AChE and predicted its binding mode. The result that was obtained showed that **C1** was able to penetrate into the enzyme’s active site gorge and bind to the AChE peripheral anionic site (PAS) as a secondary binding site (Fig. 5). It was reported before that binding to the PAS of the AChE might decrease the accumulating effects of the enzyme on the β-amyloid peptide, and hence the ability of **C1** to bind to the peripheral anionic site of AChE proves its potential as drug lead or molecular probe for the AD treatment [7].

**Fig. 6:**
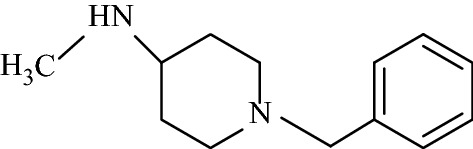
Acetamide pendent derivative **C3**

**Fig. 7:**
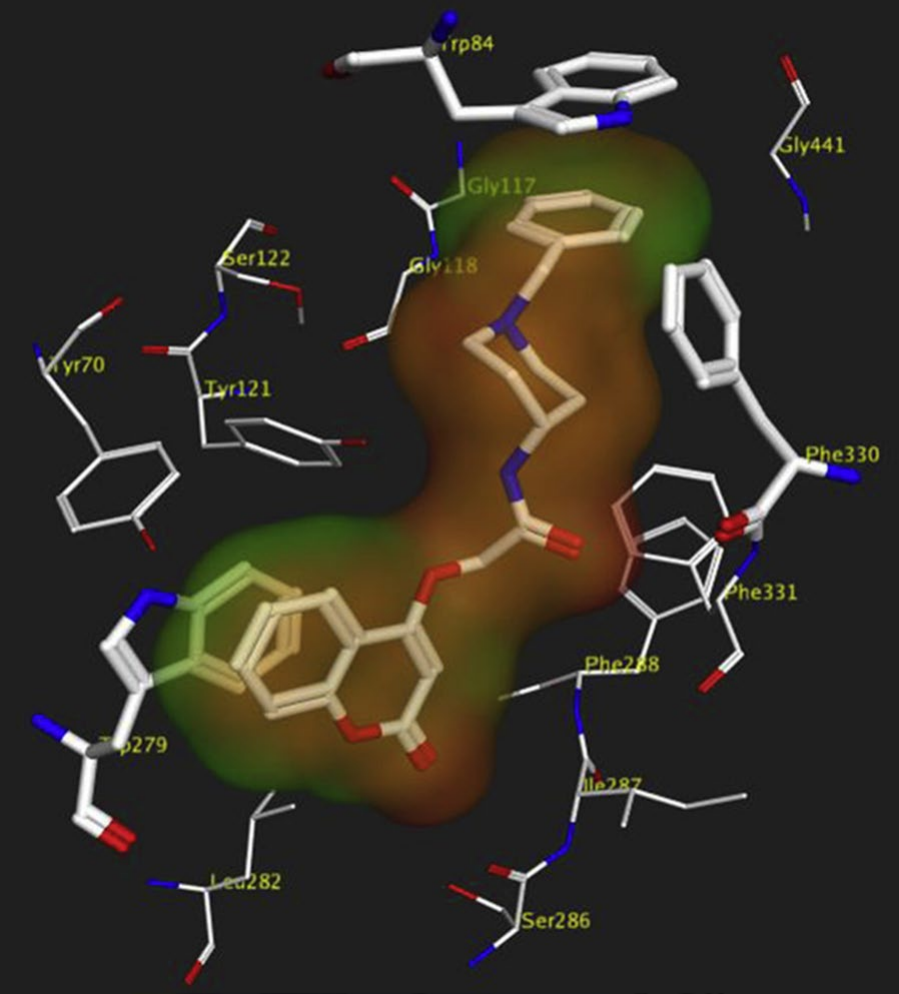
Proposed binding mode of compound **C3** within the active site of TcAChE [27]

Razavi et al. designed and synthesized a series of 4-hydroxycoumarin derivatives [27]. They screened them towards electrophorus electricus acetylcholinesterase (eelAChE) and horse serum butyrylcholinesterase (eqBuChE) using modified Ellman’s methodology, which was previously described by Kapkova et al. [28]. Commercially available donepezil, was used as the internal standard. Donepezil is one of most used AChEIs in AD therapy, acting as a dual binding site, reversible inhibitor of AChE with high selectivity over BuChE. The result obtained showed that among the 19 coumarin derivatives, the acetamide pendent (**C2**) derivative, *N*-(1-benzylpiperidin-4-yl)acetamide (**C3**) (Fig. 6), displayed the highest AChE inhibitory activity with the IC_50_ value of 1.2 μM. The increase in this activity was further justified by the help of docking study of **C3**. The best docking pose of **C3** and amino acids in the active site of *Torpedo californica* acetylcholinesterase (TcAChE) is represented in Fig. 7.

It was stated, that the type of cyclic amine attached to the 2-oxo or 4-oxoakoxycoumarin backbone influenced the increase in the inhibitory property. The strong anti-AChE activity of **C3** was found to be due to the ligand recognition and trafficking, for which Phe330 was responsible, through the formation of a π-cation interaction with the ligand, at the bottom of the active site of TcAchE. Additionally, the π–π interaction between the coumarin moiety and Trp279 of PAS was also found to stabilize the ligand in the active site of TcAchE, due to which the enzyme inhibition was more potent.

**Fig. 8:**
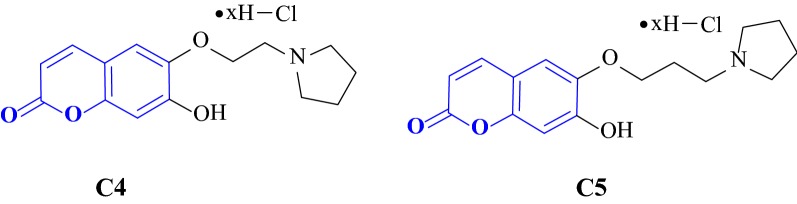
Standards scopoletin (**C4**) and galantamine (**C5**)

**Fig. 9:**
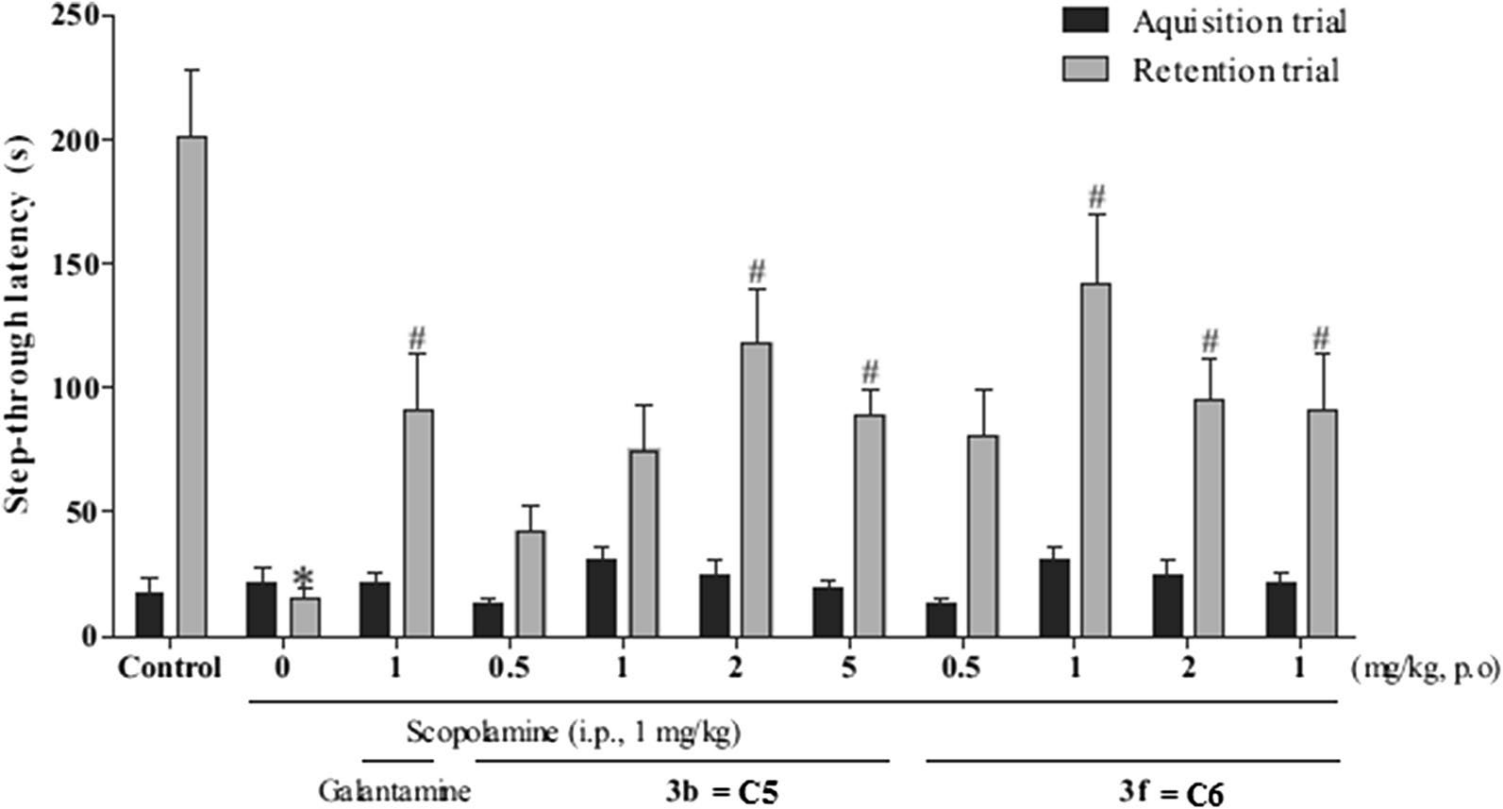
Effects of **C6**, **C7**, and galantamine on the passive avoidance task in scopolamine-induced memory deficit model. *P < 0.05 versus vehicle-treated controls. ^#^P < 0.05versus scopolamine-treated group. Data are expressed as mean ± SEM [29]

Nam et al. [29] synthesized a series of aminoalkyl coumarin hybrids based on the structure of scopoletin and tested their in vitro AChE inhibition properties using mouse brain homogenates the internal standards scopoletin (**C4**) and galantamine (**C5**) (Fig. 8). It was reported that among all the derivatives synthesized, the pyrrolidine-substituted coumarins 7-Hydroxy-6-(2-(pyrrolidin-1-yl)ethoxy)-2H-chromen-2-one hydrochloride (**C6**) and 7-Hydroxy-6-(3-(pyrrolidin-1-yl)propoxy)-2H-chromen-2-one hydrochloride (**C7**), exhibited the most potent inhibitory activities with IC_50_ values of 6.85 and 2.87 μM and compound **C7** was even found to express a 160-fold higher anti-AChE property than the lead structure scopoletin (IC_50_ = 476.37 μM) and nearly equal to that of galantamine (IC_50_ = 2.50 μM). Additionally, these derivatives also ameliorated scopolamine-induced memory deficit in mice when they were fed orally at the dose level of 1 and 2 mg/kg. The activity profiles of **C6** and **C7** are shown in Fig. 9 [29].

**Fig. 10:**
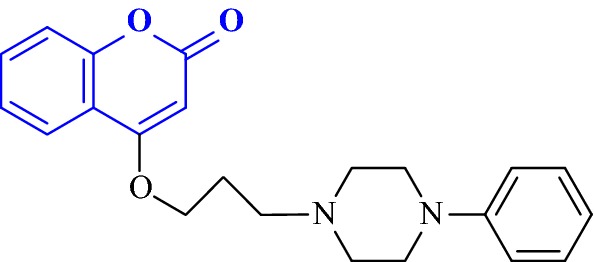
Phenylpiperazine derivative **C8**

Singla and Piplani synthesized a series of 15 novel coumarin hybrids in which coumarin moiety was linked to different substituted amines via an appropriate linker as potential inhibitors of AChE [30]. They performed the molecular docking studies in order to evaluate their potential as dual binding site acetylcholinesterase inhibitors for the treatment of cognitive dysfunction caused by increased hydrolysis of acetylcholine and scopolamine induced oxidative stress. Among all the synthesized compounds, the compound 4-[3-(4-phenylpiperazin-1-yl)propoxy]-2Hchromen-2-one (**C8**), was found to be post potent displaying higher AChE inhibitory activity of IC_50_ = 2.42 μM against the standard drug donepezil with the IC_50_ value of 1.82 μM and hence displaying significant binding interactions with both the binding pockets viz Trp86 and Trp286, respectively, of the acetylcholinesterase enzyme (Fig. 10). Molecular docking study of **C8** indicated that it interacts with all the crucial amino acids present at the catalytic active site (CAS), mid-gorge and PAS of TcAChE through hydrophobic, Van der Waal and π–π stacking interactions resulting in higher inhibitory potency of AChE enzyme as compared to other 14 analogues of the series.

**Fig. 11:**
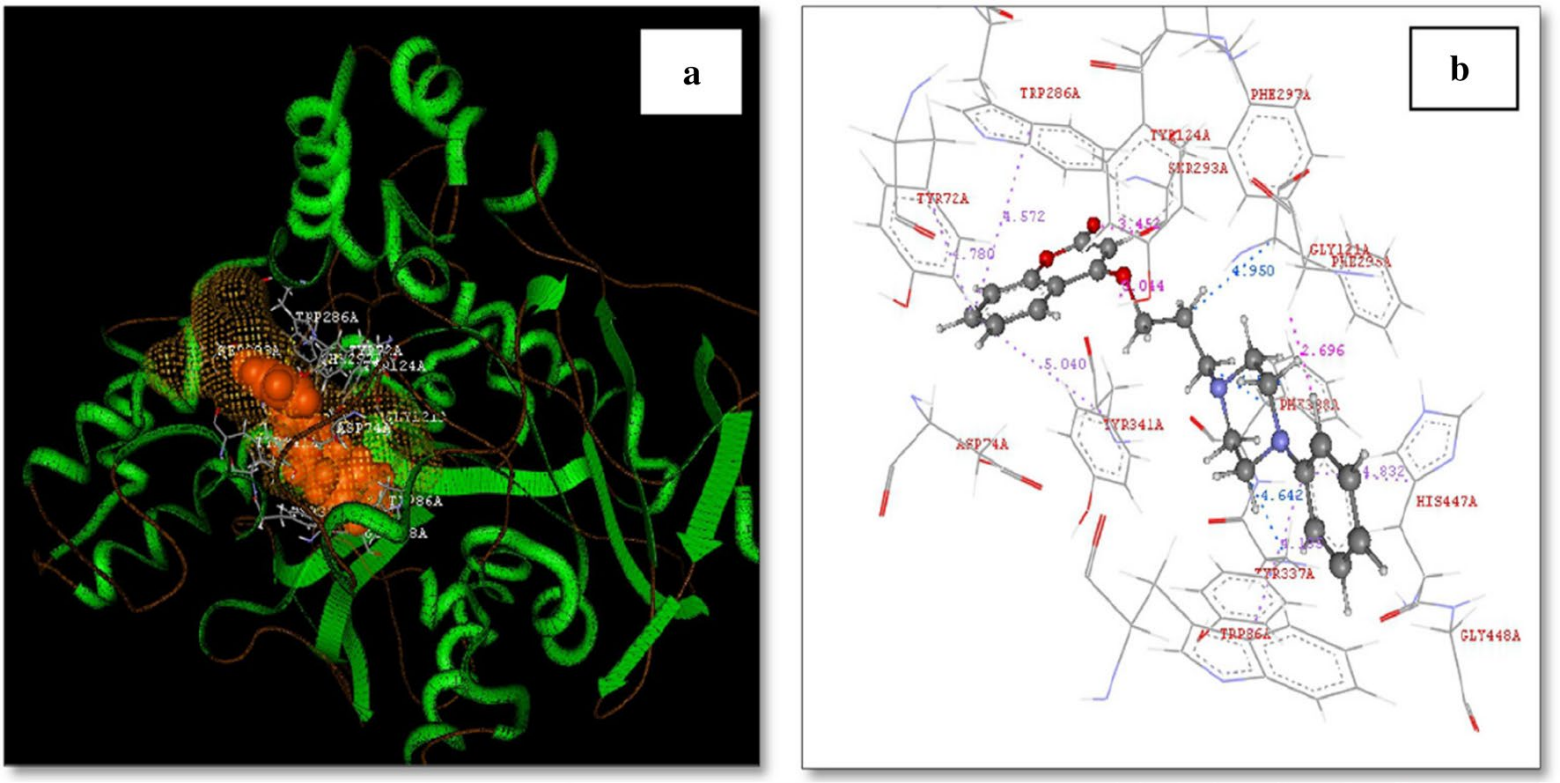
**a** 3D Orientation of the best docked pose of compound **C8** in the active site cavity of AChE. **b** Hypothetical binding motif of **C8** within the crystal structure of AChE surrounding active amino acids [30]

In detailed observation it was reported that the phenylpiperazine fragment of compound **C8** was found to enter into the gorge of the AChE enzyme, resulting in parallel π–π stacking interactions with the catalytic site of amino acids Trp86 (4.32 Å) and His447 (4.97 Å), thus adopting a sandwich like form. Coumarin moiety was also found to interact via aromatic π–π interactions with ring-to-ring distance of 4.5–4.7 Å with the indole and phenyl rings of Trp286 and Tyr72, which were located at the peripheral anionic site (Fig. 11).

**Fig. 12:**
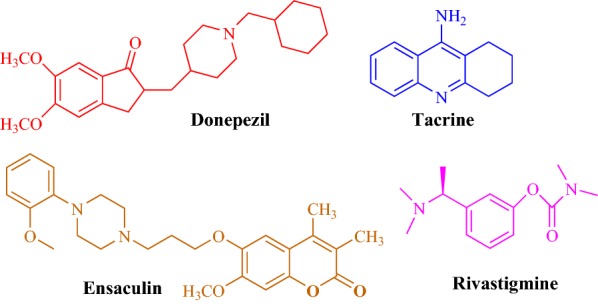
Structures of the acetylcholinesterase inhibitors as FDA approved Alzheimer’s disease therapeutics

Zhou et al. designed and synthesized three series of coumarin derivatives (Series A, B and C) with different phenylpiperazine moiety as substituents to study their potential for the treatment of AD with respect to donepezil, a standard drug [31]. While designing their series they minutely considered quite a number of factors, which influence or affect the inhibition of cholinesterase enzyme. As recent research have revealed that those compounds which can effectively dual-bind with AChE possess very good therapeutic importance as they can effectively cause the prevention of Aβ aggregation. These therapeutic acetylcholinesterase inhibitors (AChEIs) can facilitate cholinergic transmission, interfere with the synthesis, deposition and aggregation of toxic Aβ-peptides [32, 33]. Certain anti-AChEIs, which play effective role in the memory improvement and cognitive functions and are used to treat AD on clinical level for past many years are reportedly: tacrine, donepezil, rivastigmine, and ensaculin (Fig. 12). Among all these standard drugs specifically ensaculin which is a derivative of coumarin and composed of benzopyran and piperazine moiety has been used in its HCl salt form the under trade name of KA-672 HCl has been reported to slow down or prevent this progressive neurodegeneration [34, 35].

**Fig. 13:**
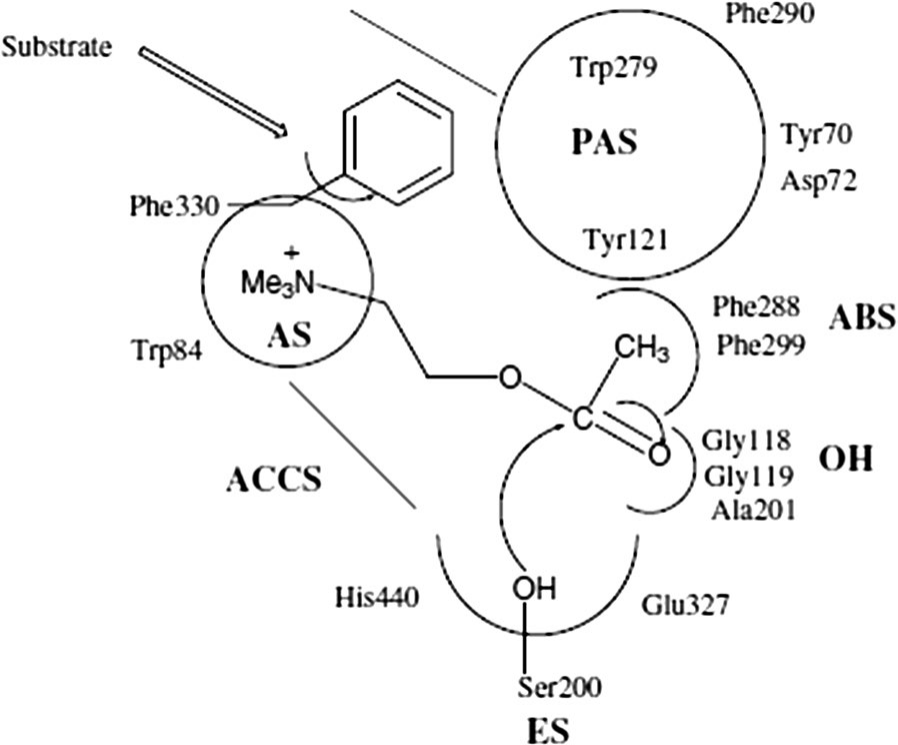
Active site and PAS of AChE. Numbers refer to residue positions in Torpedo California AChE [36]

The Torpedo California has reported the three dimensional X-ray structure of AChE for comparative study between the enzyme and the inhibitors [36] and the X-ray structure of the transition state of the AChE has also been reported [37]. Figure 13 shows the insight structure of the active sites of AChE which mainly consists of 4 binding sites: (i) Anionic substrate (AS) binding site—contains Trp84, Glu199, and Phe330 aromatic residues with negative charges where the nitrogen of quaternary ammonium group of AChE and various other positive active sites bind through interaction between the quaternary nitrogen (or other active sites) and electrons of the aromatic residues. (ii) Ecstatic site (ES): contains three residues Ser200-His440-Glu32718 which forms the catalytic triad. (iii) Acyl binding site (ABS): consists of Phe288, and Phe299, which binds to the acetyl group of AChE enzyme [33]. (iv) PAS: consists of Trp279, Tyr70, Tyr121, Asp72, Glu199, and Phe290, which can bind to 9-aminoacridine (tacrine) [38–41]. Exclusively molecules are shown to interact with PAS but can also interact with both viz catalytic ES and PAS and this helps in the prevention of pro-aggregation of AChE toward Aβ [42].

**Fig. 14:**
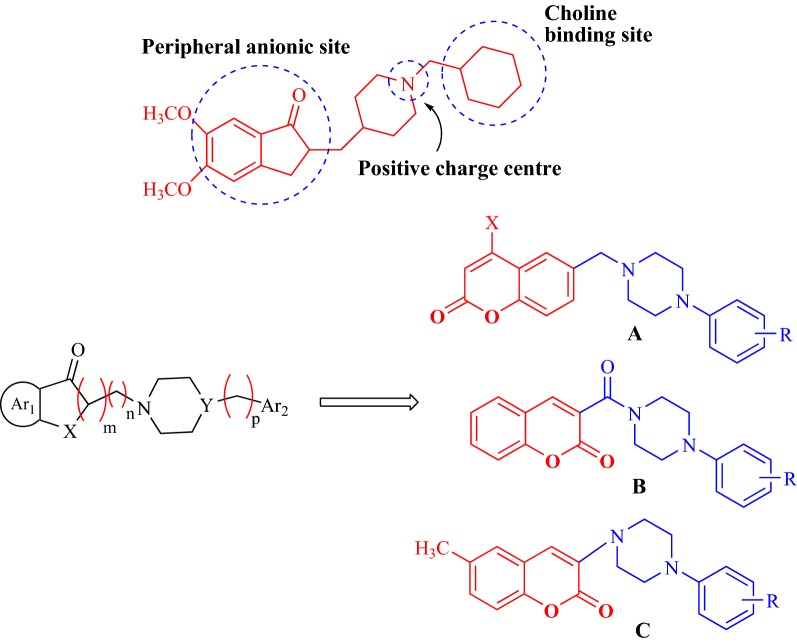
Design strategy of the target compounds of series A, B and C

These factors were the core reason behind choosing ensaculin, as the basic skeleton and designing the three series of anti-AChE coumarins analogues with phenylpiperazine substituted at position 6 of coumarin in series A, at position 3 in series B and at position 4 in series C, which were similar in structure to the frame of ensaculin (Fig. 14). Donepezil was chosen as the standard drug for comparing the obtained results.

**Fig. 15:**
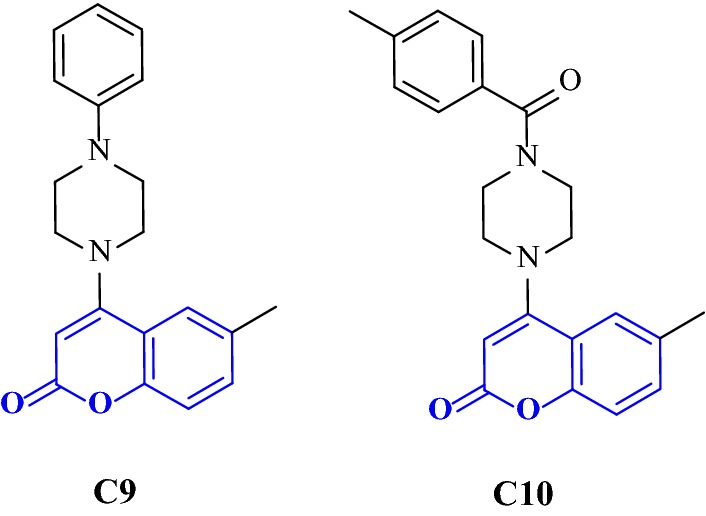
4-Phenylpiperazine substituted coumarin derivatives **C9** and **C10**

The anti-AChE results concluded that coumarin derivatives with substitution at position 3 (series B) and 4 (series C) of the coumarin ring were better that the derivatives with substitution at position 6 (series A). On comparison of series A with ensaculin, the reason behind the dullness was clear. It was concluded that the presence of only one atom in the linking chain between coumarin skeleton and phenylpiperazine moiety in series A was the cause for this as it cannot reach the requirement for gorge, with respect to the presence of four atoms in ensaculin [33]. The two most potent 4-phenylpiperazine substituted coumarin derivatives of series C were 6-methyl-4-(4-phenylpiperazin-1-yl)2H-chromen-2-one (**C9**) and 6-methyl-4-(4-(4-methyl-benzoyl)piperazin-1-yl)2H-chromen-2-one (**C10**) with IC_50_ value of 4.5 and 5.3 μmol/L. The distance between carbonyl-carbon atom and nitrogen atom of piperazine ring was in agreement with donepezil hence making these 4-substituted compounds good anti-AChE inhibitors (Fig. 15).

## Coumarin analogues as dual AChE/BuChE inhibitors

**Fig. 16:**
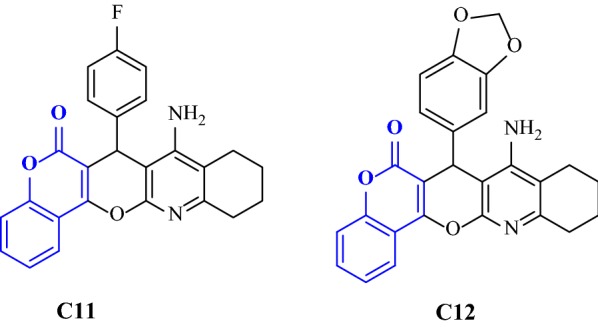
Molecular structure for **C11** and **C12**

Khoobi et al. have designed and synthesized novel hybrid derivatives of 2 known scaffolds which are tetrahydroaminoquinoline and coumarin to form novel series of 8-amino-tetrahydrochromeno[3′,4′:5,6]pyrano[2,3-b]quinolin-6(7H)-one. These derivatives were screened for their AChE/BuChE inhibitor activities using colorimetric Ellman’s method. The result obtained showed that compound 8-amino-7-(4-fluorophenyl)-9,10,11,12-tetrahydrochromeno[3′,4′:5,6]pyrano[2,3-b]quinolin-6(7H)-one (**C11**) was the most active compound against eelAChE whereas compound 8-amino-7-(benzo[d][1,3]dioxo-5-yl)-9,10,11,12-tetrahydrochromeno [3′,4′:5,6]pyrano [2,3-b]quinolone-6(7H)-one (**C12**) showed the most potency as BuChE inhibitor (Fig. 16) [8].

**Fig. 17:**
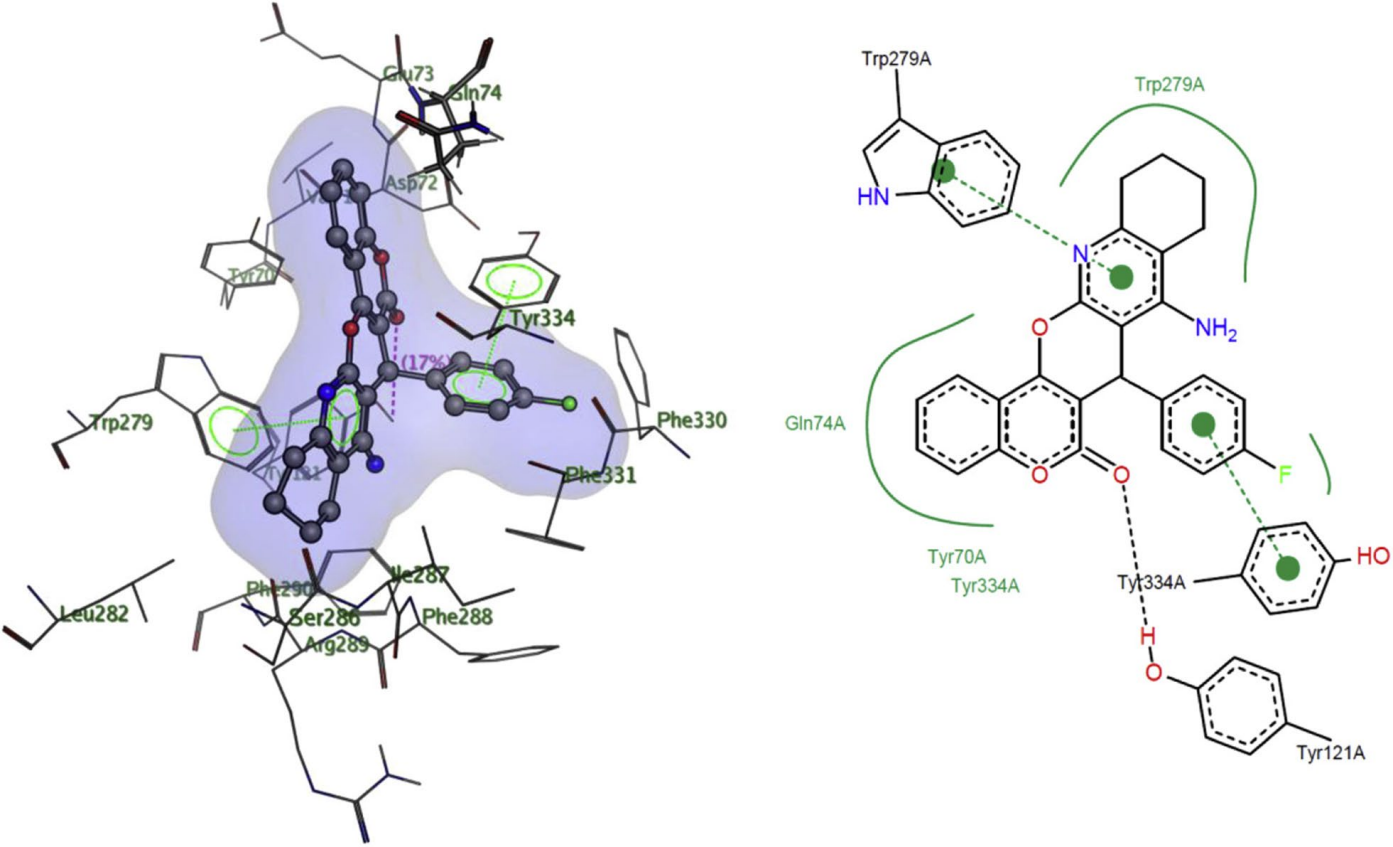
Residues involved in the interactions with **C11** and the 2D representation of binding mode of **C11** in the gorge of AChE [8]

Khoobi et al. stated that the increase in the inhibitory activity of compound (**C11**) might have been caused by the insertion of aromatic groups at position 7, and the presence of electron withdrawing substituents that is fluoro at position 4, that was able to form π–π interaction in the hydrophobic cavity. In addition, the increased activity of (**C12**) was due to the lipophilic interaction of electron donating groups such as methyl and methoxy with the active site. They carried out a kinetic study on compound **C11** to determine the kinetic type of AChE inhibition and the result showed that it possessed a mixed type inhibition where it can interact with both, PAS and CAS. To identify the binding mode of compound **C11**, it was docked at the gorge of eelAChE. The obtained result showed a proper fitting of compound **C11** in the gorge of eelAChE (Fig. 17). The phenyl ring at position 7 was turned towards the hydrophobic pocket of the binding cavity composed of Phe330, Tyr334 and Phe331, and the ligand-receptor complex was stabilized by the π–π stacking between the phenyl side chain of Tyr334 and the phenyl moiety of compound **C11**. π–π stacking between pyridine ring and indole side chain of Trp279 was able to donate specific conformation to the compounds so that the lipophilic cyclohexane ring gets fitted in the hydrophobic packet composed by Phe290, Leu282, Phe288, Ile287 and Ser286 whereas the coumarin carbonyl moiety forms a hydrogen bonding with hydroxyl of Tyr121 and CH-π stacking between side chain of Gln74 and phenyl ring of coumarin scaffold. Based on the results obtained, the hybrid of tetrahydroaminoquinoline and coumarin scaffold designed and synthesize by Khoobi et al. can undergo further modifications and proposes as promising lead structure.

**Fig. 18:**
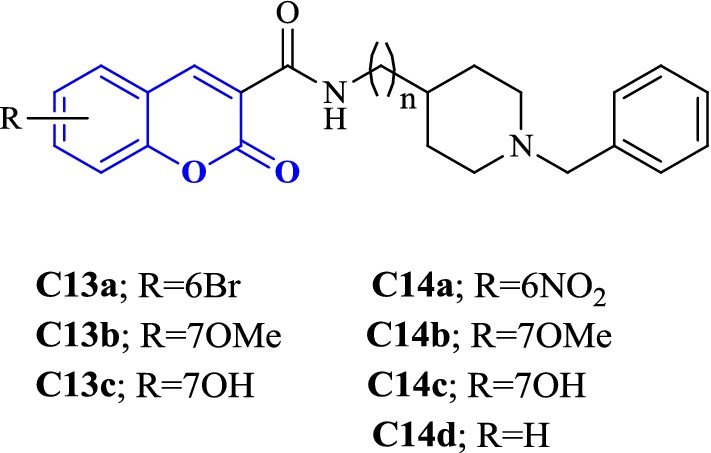
Chemical structures of coumarin-3-carboxamides

Asadipour et al. designed novel coumarin-3-carboxamides bearing *N*-benzylpiperidine moiety as potent AChE/BuChE inhibitors (Fig. 18). The docking result concluded that most of the synthesized hybrids were potent anti-AChE and among all the compounds, compound (**C14a**) displayed the most potent activity as anti-AChE inhibitor with IC_50_ of 0.3 nM, which was almost 46-fold more than the standard drug donepezil [10]. In depth structural study of protein–ligand demonstrated, that the synthesized compounds possessed dual binding site interaction mode, which was also in agreement with the performed kinetic studies. Recent studies have reported that dual inhibitory properties might help in improving the symptoms related with dementia and treating AD [43, 44]. This dual interaction mode of the compounds was due to its designing. The compounds were synthesized by linking two fragments, *N*-benzylpiperidine (CAS binding motif) and coumarin (PAS binding motif) with two types of linkers, carboxamide or *N*-ethylcarboxamide. It was reported that the compounds with *N*-ethylcarboxamide linker were more active than their counterparts with carboxamide linker, which can be seen for compound **C14a** having a nitro group at position 6 of the coumarin ring. Presence of various substituent were found contributing for different level of anti-ChE activity. Bromo, nitro and methoxy substituents were reported to increase the anti-AChE activity whereas hydroxyl group at position 6 or 7 of the coumarin ring was found decreasing the AChE inhibitory activity. Comparison of 7-methoxy analogues (**C13b**, carboxamide linked and **C14b**, *N*-ethylcarboxamide linked) with the 7-hydroxy counterparts (**C13c**, carboxamide linked and **C14c**, *N*-ethylcarboxamide linked) revealed, that O-methylation of 7-hydroxy coumarins improves the AChE activity. Compounds of *N*-ethylcarboxamide series were also found to show higher BuChE inhibitory activity with the IC_50_ value ≤ 420 nM as compared to the compounds of carboxamide series with the IC_50_ value of ≥ 20 μM. Additionally, it is quite interesting to find out that the position of methoxy group in 7- and 8-methoxy isomers, (linked through *N*-ethylcarboxamide) was influencing BuChE/AChE selectivity in 71 versus 9000 ratio. However, *N*-ethylcarboxamide linked compound **C14d**, was reported as a dual cholinesterase inhibitor with more favorable balancing between AChE/BuChE inhibitions as compared to the standard drug donepezil. It was found to display anti-AChE activity with IC_50_ 26 nM and anti-BuChE activity with IC_50_ 371 nM.

**Fig. 19:**
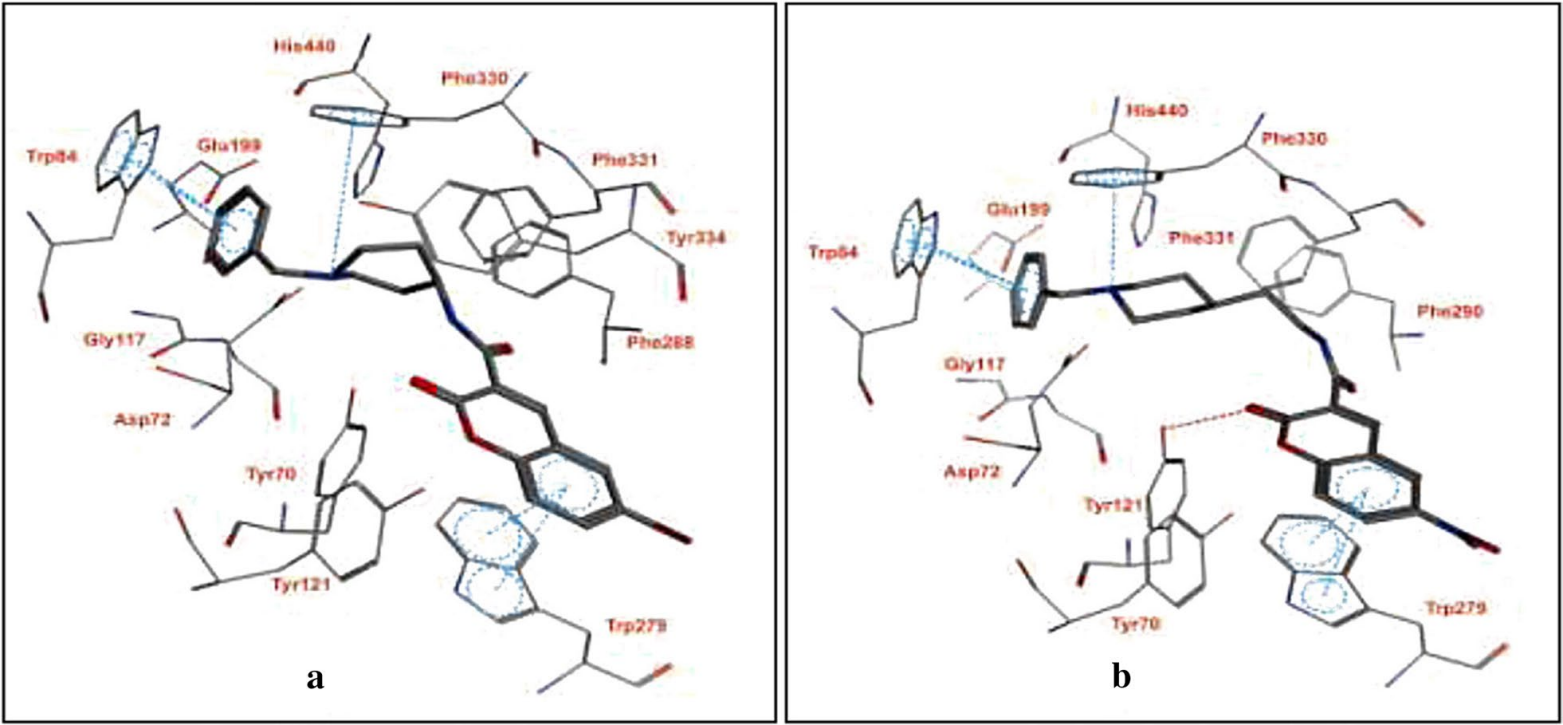
**a** Representative model for interactions of compounds **C13a**. **b C14a** docked into the binding site of AChE. Hydrogen bond is indicated as red dotted line [10]

**Fig. 20:**
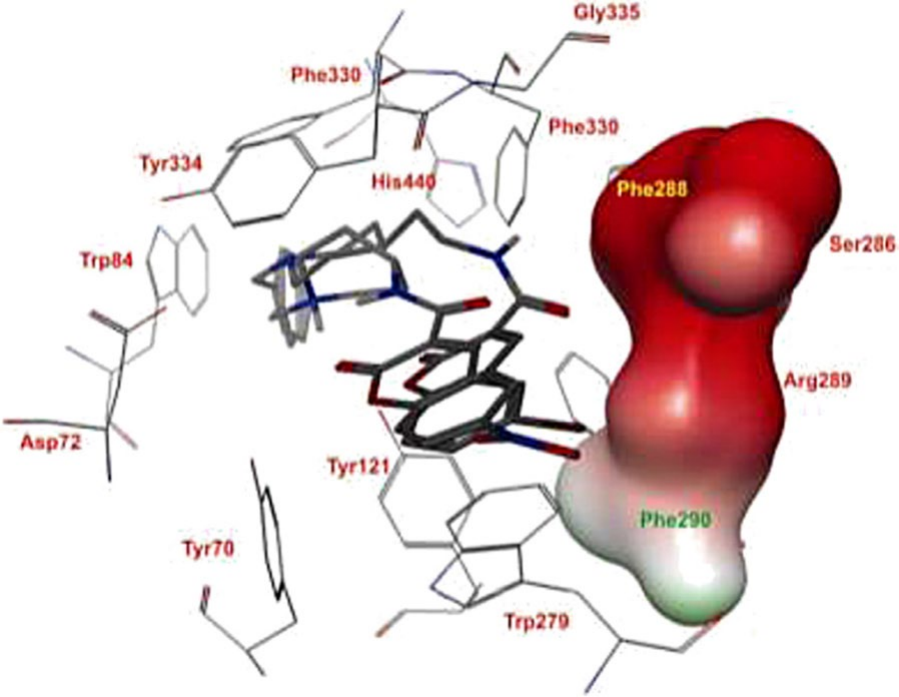
The relative orientation of carboxamide linker of the best docking poses of **C13a** and **C14a**. The carboxamide linker of **C14a** oriented to a hydrophillic pocket (red surface) of the active site [10]

In order to get the insight detail of the binding modes and structural modifications of the most active compounds from the carboxamide (**C13a**) and *N*-ethylcarboxamide (**C14a**) linked series. Asadipour et al. performed automated docking against AChE using the optimized parameters. Both the active molecules were found to behave similarly in terms of orientation to that of the standard donepezil within the active site. This reveals the fact that the *N*-benzylpiperidine fragment of the compounds helped them to anchor in the midgorge of the enzyme and the coumarin part was accommodated in the rim of the gorge (Figs. 19 and 20). The good anti-ChE activity of the compounds of *N*-ethylcarboxamide linker series over carboxamide linker series was found to due to their more favourable interactions with the targeted enzymes. The *N*-ethylcarboxamide linked compound **C14a** was observed to form hydrogen bond in between the carbonyl of coumarin and hydroxyl of Tyr 121. Moreover, the carbonyl group of its amide moiety was also to be directed towards the hydrophobic pocket comprising of Ser286, Phe290, and Arg 289. Both these two interactions were not observed for the compound of carboxamide linker series (**C13a**). The common interactions, which were observed for both the linker compounds, were the π–π interaction observed due to the parallel disposition of the phenyl ring of benzyl moiety to Trp84, a π-cation between the quaternary nitrogen of piperidine ring with Phe330 and a π–π stacking of coumarin ring and Trp279.

**Fig. 21:**
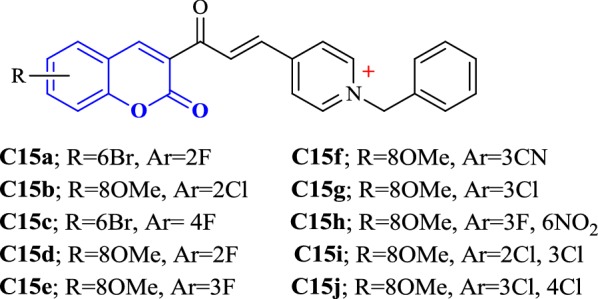
*N*-Benzyl pyridinium coumarin derivatives

Alipour et al. synthesized novel coumarin derivatives (Fig. 21) as potent and dual binding site acetylcholinesterase inhibitors bearing *N*-benzyl pyridinium moiety which was attached to the coumarin nucleus via alpha beta unsaturated carbonyl linker. The reason behind the introduction of this linker was its conformational restriction caused by the presence of conjugated double bond which makes the molecule free from any conformational alternations and helps in further study related to substituent modifications. Most of the designed compounds were found to exhibit IC_50_ values in nanomolar range and among all, compound (E)-4-(3-(6-Bromo-2-oxo-2H-chromen-3-yl)-3-oxoprop-1-enyl)-1-(2-fluorobenzyl)pyridinium chloride (**C15a**) was found to be the most active against acetylcholinesterase enzyme displaying IC_50_ value of 0.11 nM and compound (E)-1-(2-Chlorobenzyl)-4-(3-(8-methoxy-2-oxo-2H-chromen-3-yl)-3-oxoprop-1-enyl)pyridinium chloride (**C15b**) gave the most potent inhibition of BuChE (IC_50_ = 125 nM). Alipour et al. observed that the steric and electronic features of the substituents in the coumarin nucleus played a great role on the anticholinesterase activity of the target compounds. They reported that the movement of fluoro substituent from ortho-position (**C15a**) to para-position (**C15c**, (IC_50_ = 0.46 nM)) causes a 4 times decrease in the inhibition properties.

**Fig. 22:**
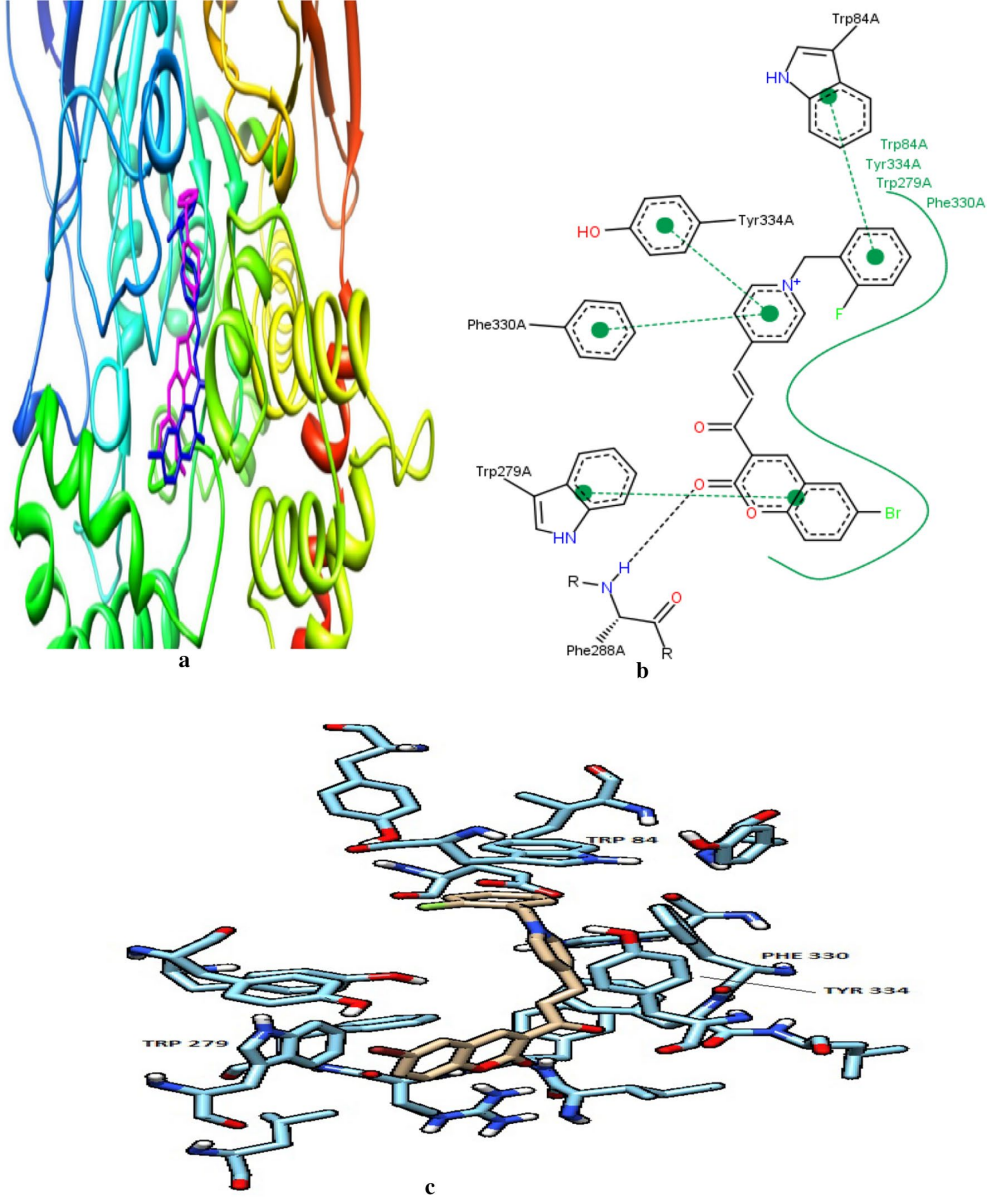
**a** Superimposition of **C15a** (blue) and donepezil (magenta) in the gorge of TcAChE. **b** 2D representation of binding mode of **C15a** and amino acid residues in the gorge of TcAChE created by PoseView. **c** Docking of **C15a** in the active site of TcAChE [45]

These observations were also in agreement with the docking studies. Fluoro at ortho-position was found to disrupt the π–π stacking interactions due to the rotation of the phenyl ring, which was in the other case observed for compound **C15d**. But the movement of fluoro to meta-position (**C15e**) increased the inhibitory properties due to proper stacking of the phenyl ring along with Trp84. Flouro at para-positionagain found to reduce the activity because of the steric hindrance with the amino acids present at the bottom of AChE gorge (Fig. 22). Additionally, larger sized substituents (**C15b**; IC_50_ = 0.46 nM) were found to enhance the inhibitory activity regardless of their electronic properties as compared to the smaller sized substituents (**C15d**; IC_50_ = 26 nM). Moreover, the electron-withdrawing nature of the substituents present at position 3 of the benzyl moiety were found to enhance the activity according to their strong electron withdrawing nature **C15e** (F, IC_50_ = 0.47 nM) > **C15f** (CN, IC_50_ = 76 nM) > **C15g** (Cl, IC_50_ = 86 nM), whereas the insertion of any substituent at any position of the benzyl ring exceptionally decrease the inhibitory activities of the target compounds as observed for **C15h** (IC_50_ = 330 nM), **C15i** (IC_50_ = 20 nM) and **C15j** (IC_50_ = 440 nM) [45].

**Fig. 23:**
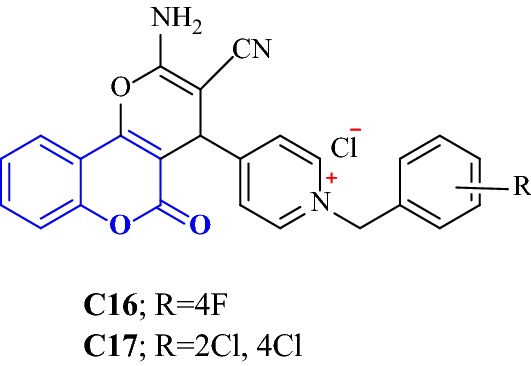
Chemical structures for 5-oxo-4,5-dihydropyrano coumarin derivatives

Khoobi et al. [8] designed and synthesized 5-oxo-4,5-dihydropyrano[3,2-c]chromene derivatives and attached them to *N*-benzylpyridinium scaffold before subjecting them for their acetylcholinesterase (AChE) and butyrylcholinesterase (BuChE) inhibitory activities against the standard drug donepezil. From their findings, they found out that the 4-(2-Amino-3-cyano-5-oxo-4,5-dihydropyrano[3,2-c]chromen-4-yl)-1-(4-fluorobenzyl)pyridinium chloride derivative (**C16**) was the strongest AChE inhibitor with IC_50_ value 0.038 μM and strongest AChE/BuChE selective with SI value of more than 48 and compound 4-(2-Amino-3-cyano-5-oxo-4,5-dihydropyrano[3,2-c]chromen-4-yl)-1-(2,4-dichlorobenzyl)pyridinium chloride (**C17**) was the strongest BuChE inhibitor with IC_50_ value of 0.566 μM (Fig. 23).

**Fig. 24:**
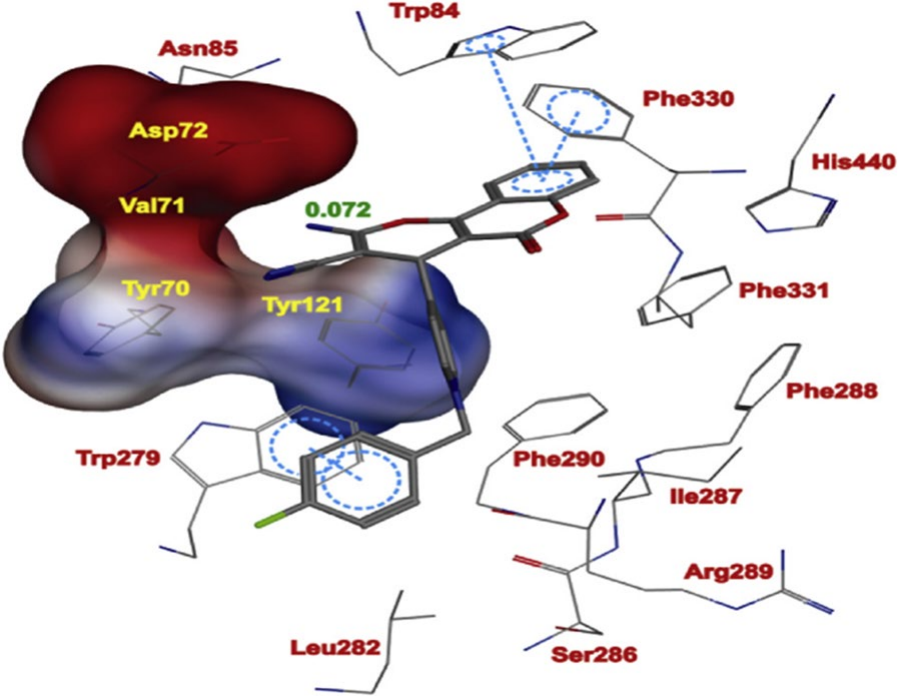
A representative model for interaction of compound **C16** and the AChE [46]

To get the insight information related to structure activity relationship and possible binding modes they docked this compound into the active site of the enzyme using Autodock Vina 1.1.1. The ability of the target compounds to interact with the two distinct regions of the active site, which are the CAS and PAS is their most common feature. Within the active site of the enzyme AChE, the vicinity portion of the catalytic site (CS) is called as the central CAS and the top position of the gorge of AChE is called as PAS. The residues importantly responsible for the interaction with the target compounds (inhibitors) are Phe330, Trp84 from CAS to Trp279 from PAS to in all these Phe330 is the key residue for ligand recognition and trafficking. As the chemical space (inhibitor) is made up of two recognized fragments which are pyranochromen-2-one, and *N*-benzylpyridinium, therefore the pyrano chromene pendent of the inhibitor was found to oriented in such a manner that a π–π interaction was achieved with Phe330 and Trp84 and the positively charged amino group on the pyran ring was prone to negative area by the Asp72 of carboxylic acid side chain. In addition to these a π–π interaction between the benzyl group on pyridinium ring and Trp279 further stabilized the resulted complex (Fig. 24).

**Fig. 25:**
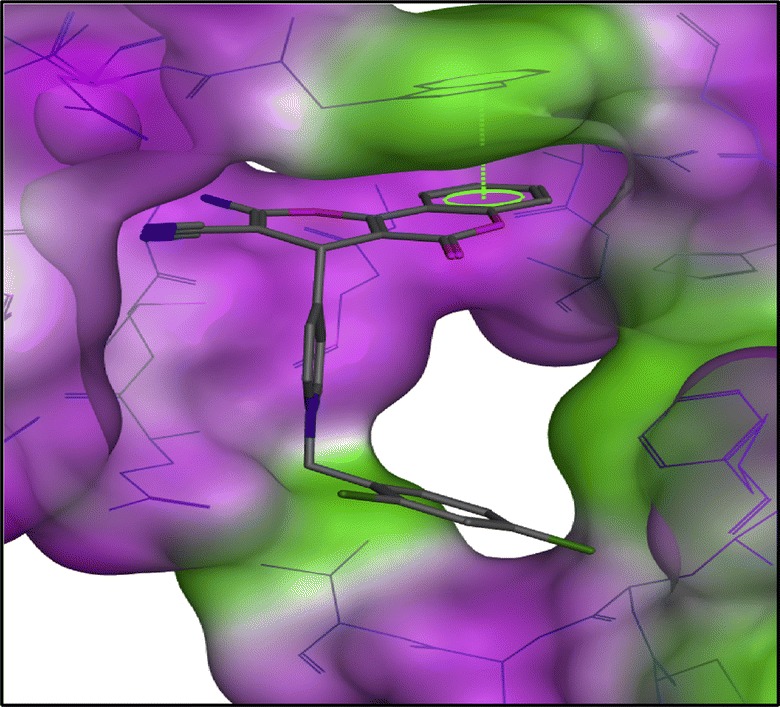
The predicted binding mode of compound **C17** in the active site of BuChE [46]

Same ways compound **C17** was docked against BuChE and its mode of interaction is depicted in Fig. 25, where a π–π stacking between the coumarin ring and Trp79 is quite clear. The orientation of the ligand (inhibitor/target compound) resulted in the exposure of the partially charged amino and nitrile groups to the oxyanionic hole composed of carbonyl moieties of Gln64, Asn65, Asp67, Asn80, Thr117 and Gly118. Additionally the hydrophobic moiety of the ligand i.e. 2,4-dichlorophenyl moiety was found to best fit in the hydrophobic pocket composed of Pro282, Leu283, Phe326, Tyr329 and Gly330 [46].

**Fig. 26:**
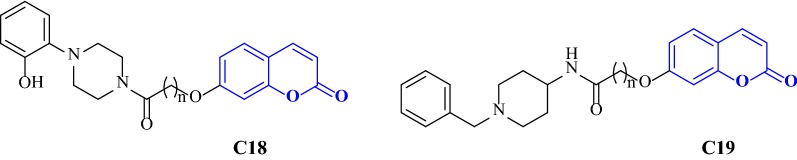
Benzylpiperidinylamino derivative **C18** and (2-fluorophenyl)piperazine derivative **C19**

Alipour et al. [47] designed and synthesized the derivatives of 7-hydroxy coumarin and attached them to various amines via an amidic linkage, and further checked their potential as AChE and BuChE inhibitors using donepezil as a standard drug. Interestingly, benzylpiperidinylamino derivative, *N*-(1-benzylpiperidin-4-yl)acetamide (**C18**) was found to be the most potent compound against AChE displaying a very good IC_50_ value of 1.6 μM and the (2-fluorophenyl)piperazine derivative (**C19**) was the most potent compound against BuChE with IC_50_ 15 μM. It is important to mention that the effect of structural modification was similar for both the activities but the most active compound against AChE was not the most active against BuChE. Therefore, further docking studies were performed in order to get the clue behind this interesting behaviour of **C18** (Fig. 26).

**Fig. 27:**
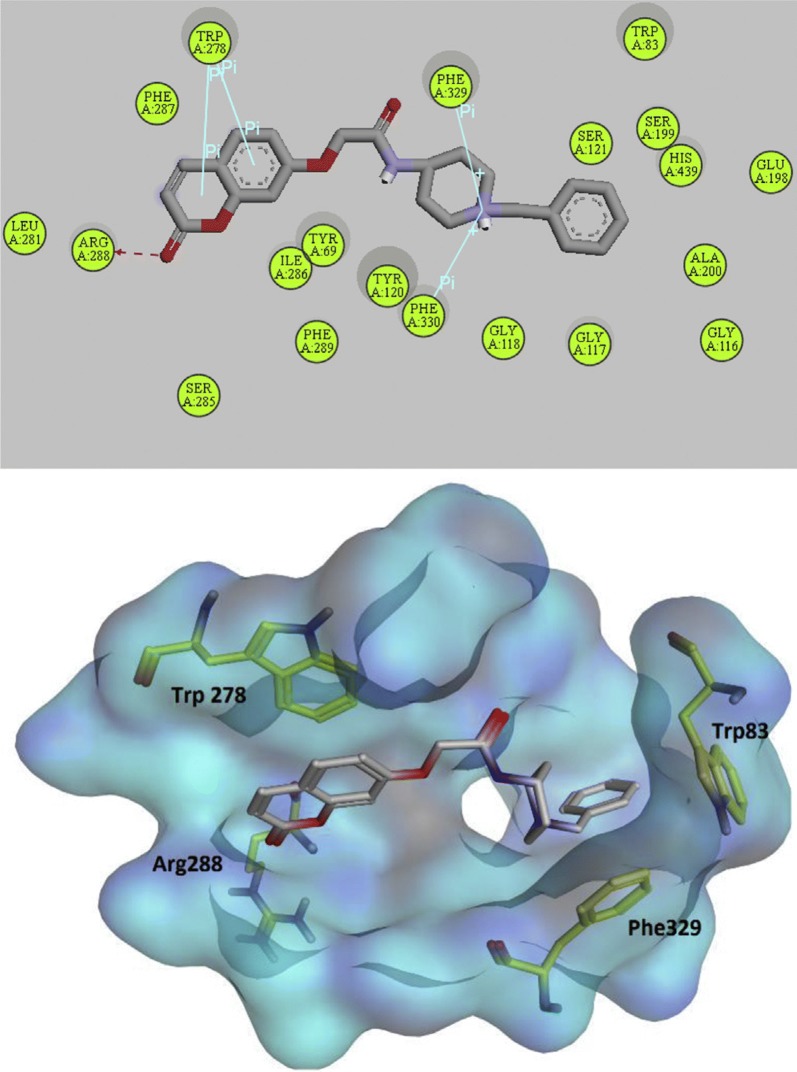
The schematic 2D and 3D representations of compound **C18**, docked in the active site of AChE [47]

Autodoc Vina program was used to examine the best-docked pose of all the synthesized inhibitors. It was found that all the inhibitors were oriented similarly into the active site of the enzyme. However, for the most active compound **C18**, interesting results were noted down (Fig. 27). The ligand was found to be nicely accommodated in the gorge of AChE active site, in such a manner that the benzylpiperidin moiety was noted to lean towards CAS. A T-shape edge-to-edge π–π stacking interaction of phenyl ring against Trp83 was observed, specifically and piperidine ring at the vicinity of Phe329 and phe330 achieved a π-cation interaction. Nevertheless, coumarin ring was noted to gain a π-stacking with the aromatic ring of Trp278 in PAS and its carbonyl moiety was found bonded to Arg288 via hydrogen bond. In this binding mode CAS and PAS both were well occupied by the ligand and hence it is in agreement with the mixed mode inhibition pattern of **C18** [47].

**Fig. 28:**
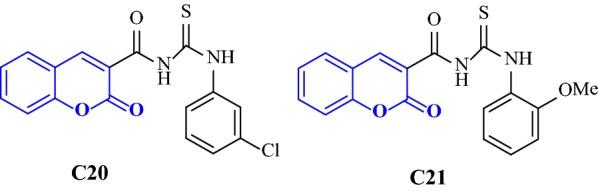
Chemical structure for thiourea coumarin derivatives **C20** and **C21**

Saeed et al. [48] synthesized a series of coumarin linked thiourea derivatives and tested their potential inhibitory activity against AChE and BuChE. Among all the compounds synthesized specifically compounds 1-(2-Oxo-2H-chromene-3-carbonyl)-3-(3-chlorophenyl)thiourea (**C20**) and 1-(2-Oxo-2H-chromene-3-carbonyl)-3-(2-methoxyphenyl)thiourea (**C21**) were found to be strongest inhibitors against AChE and BuChE with IC_50_ values of 0.04 and 0.06 μM, respectively (Fig. 28). Docking studies were performed to get the detail of the inhibitory behaviour and probable binding modes of all inhibitors against both cholinesterase using the standard drug donepezil with especial focus on the most active compounds.

**Fig. 29:**
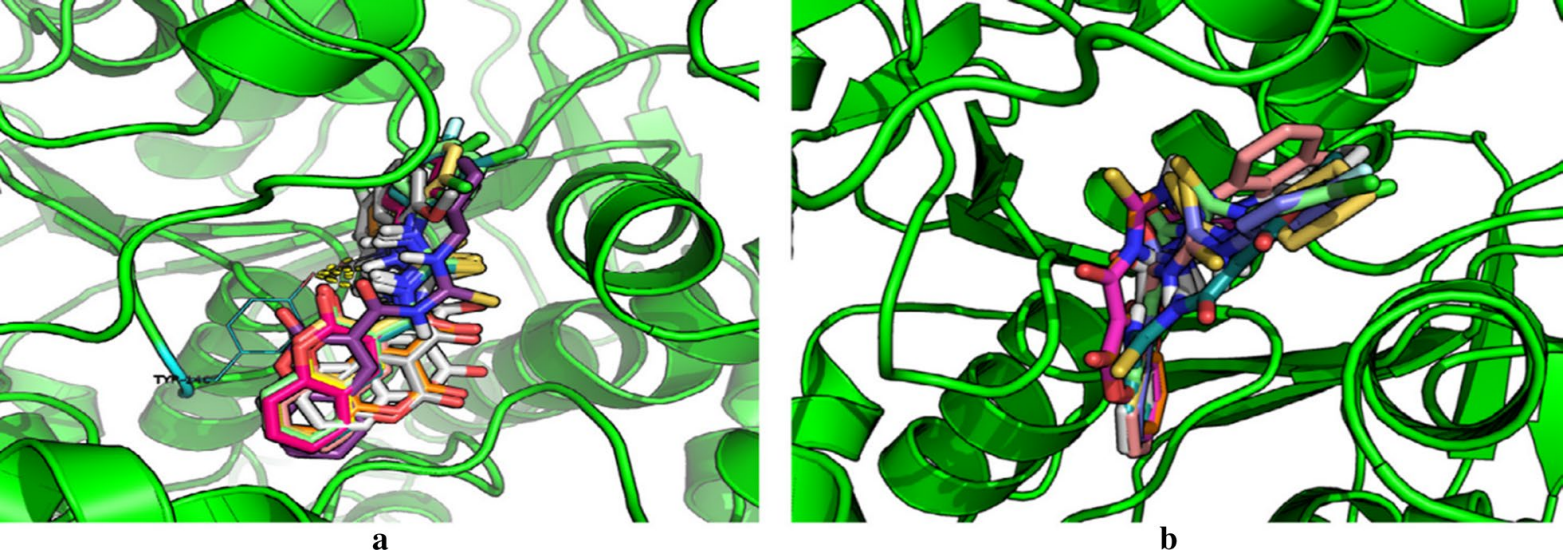
**a**, **b** Binding models of the synthesized compounds in AChE and BuChE enzymes active sites. Yellow dashed lines in (**a**) showing hydrogen bonding between Tyr146 and one of thioureas hydrogen atom from different compounds [48]

Docking results concluded similar binding modes with different docking scores for mostly all of the compounds which were found to be well docked near the catalytic triad of AChE (Glu225, Ser226, His466) forming hydrogen bond with Tyr146 via O-HAN interaction (Fig. 29a) but slightly different binding modes in the catalytic triad of BuChE (Glu224, Ser225, His 494) with no hydrogen bonding which might be due to their slightly different active site architecture (Fig. 29b) [48].

**Fig. 30:**
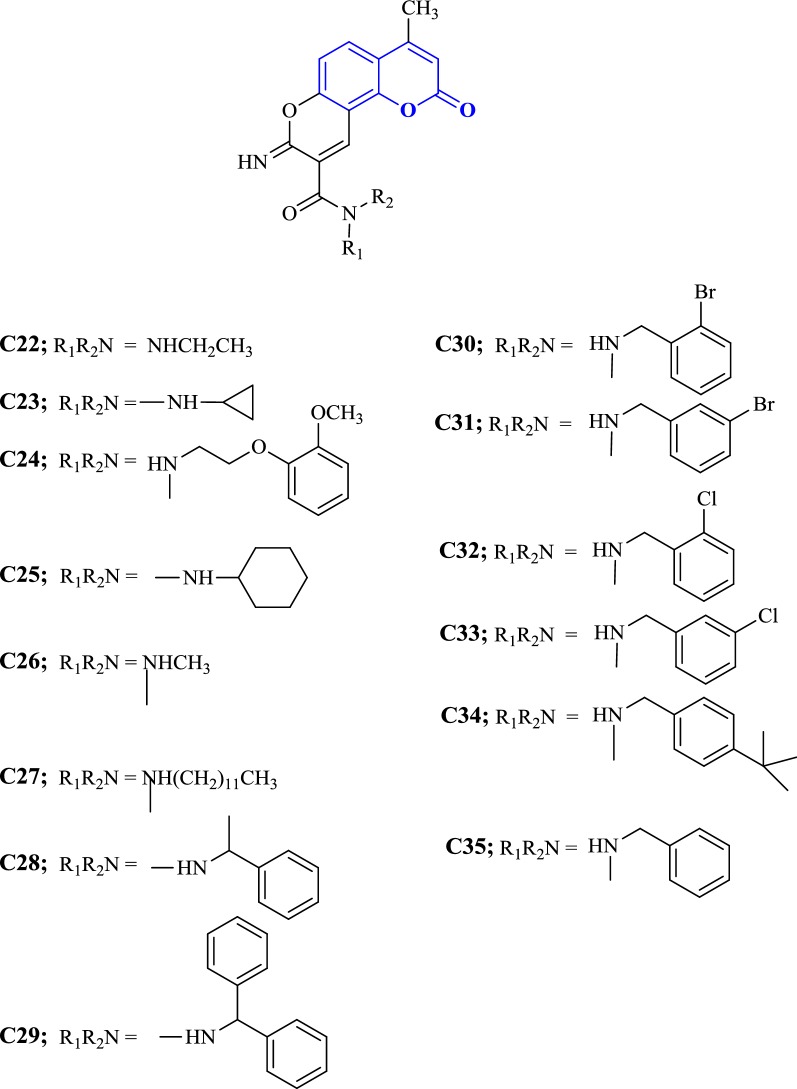
Chemical structures of tricyclic coumarin derivatives **C22**–**C35**

Shaik et al. [49] designed tricyclic coumarin derivatives bearing iminopyran ring connected to different amido moieties and talked in detail about their in vitro dual AChE/BuChE inhibitory properties against galantamine, tacrine, donepezil and rivastigmine as positive controls (Fig. 30). Compound **C31** was specifically the most potent AChE inhibitor with IC_50_ 0.003 μM and **C22** was the most potent inhibitor of BuChE with IC_50_ 11.32 μM. Importantly, compounds **C30**, **C31**, **C32**, **C33** and **C34** were found to be overall stronger anti-AChE inhibitors than all the chosen reference compounds. Compounds **C22**, **C23** and **C24** were reported as dual inhibitors of both the enzymes. It was interesting to note the relation between the structure–activity relationships, and the effect of the substituents in phenyl ring of amide moiety in the enzyme inhibition. The cyclohexyl moiety (**C25**) on amido part was reported to increase the anti-AChE activity to about 35-fold as compared to the cyclopropyl moiety (**C23**) on the amido part of the molecule. Elongation of the lipophilic side chain in compounds **C26** (IC_50_ = 1.4 μM), **C22** (IC_50_ = 0.249 μM) and **C27** (IC_50_ 0.022 μM) also increased the AChE inhibitory activity. The longer the aliphatic chain (**C27**) the stronger was its anti-AChE activity. On the other hand the BuChE inhibitory activity was limited to *N*-ethyl group from *N*-methyl group. By changing the aliphatic methyl group present at the benzylic junction (**C28**) with the aromatic phenyl ring (**C29**) at the same junction though increased the AChE activity but decreased the BuChE activity. The electron withdrawing substituent (–Cl, –Br) present at othro or meta positions of the benzyl moiety made the compounds 110–700 fold more potent (**C30**, **C31**, **C32**, and **C33**) than compounds with unsubstituted benzyl moiety (**C35**). In addition to these compound **C34** with para-(ter-butyl) benzyl moiety was also found to be 210 folds more potent than compound **C35**.

**Fig. 31:**
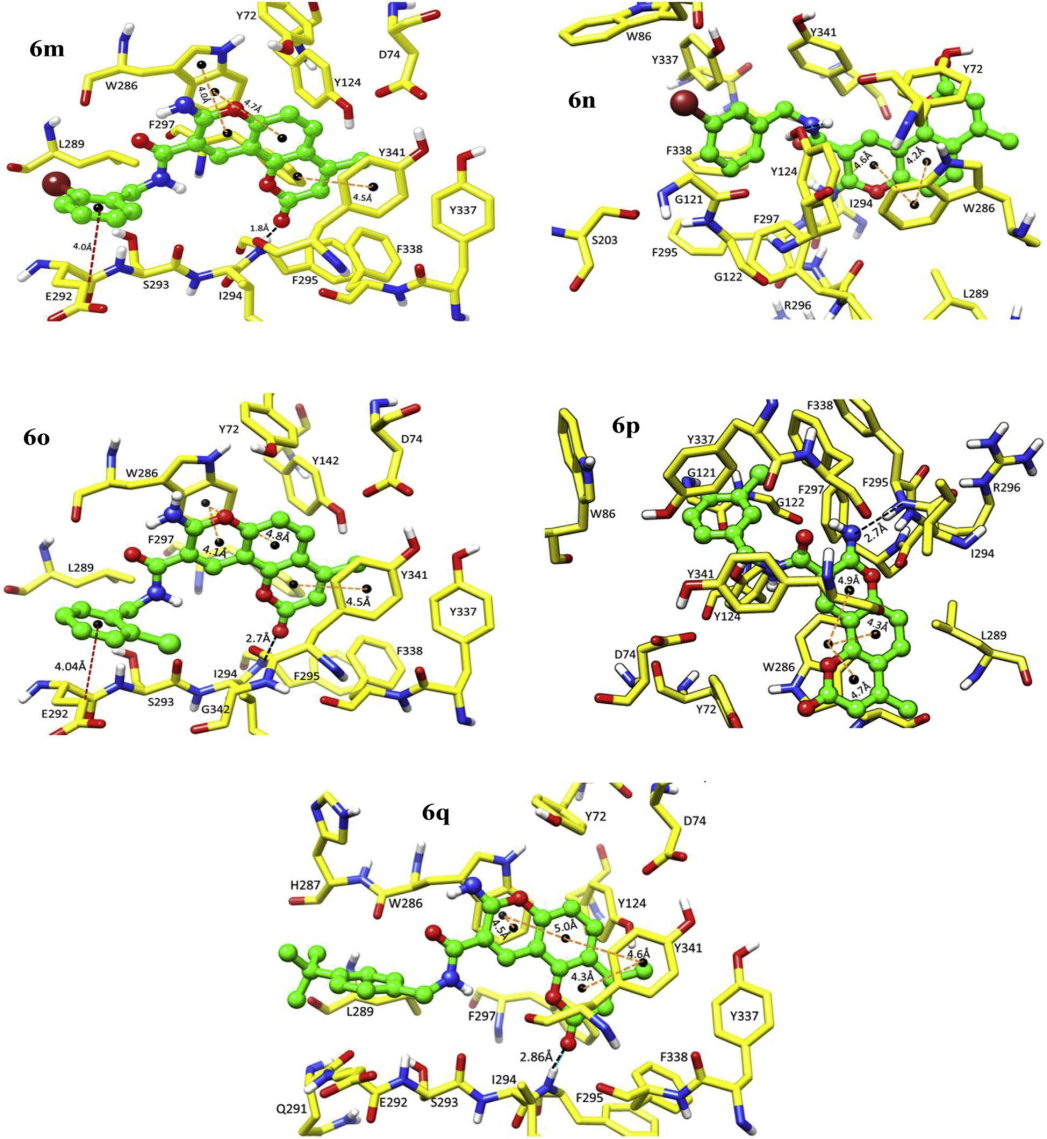
Docking pose of compounds **C30**–**C34** at the binding site of AChE [49]

In order to resolve the factors affecting the AChE activity Schrödinger maestro software was used to investigate the possible binding modes of the top 5 anti-AChE compounds viz **C30**–**C34** and therefore they were docked into the active site of the eelAChE. Compound **C30**, **C32** and **C34** were found to be similarly oriented into the active site of the enzymes whereas the binding modes of compounds **C31** and **C33** was found to be different from the binding modes of **C30**, **C32** and **C34**. The orientation of compounds **C30**, **C32** and **C34** accommodated the ligand in the PAS in such a way, that the tricyclic coumarin ring was noted to be sandwiched between Trp286 and Tyr341 via π–π stacking while the 2-bromo benzylamido moiety protruded to the opening of the PAS and was found bounded to Glu292 via CH-π interaction and a hydrogen bond interaction was achieved between the carbonyl moiety and the hydroxyl group of Phe295. In addition to these it was also noted importantly that compounds **C30**, **C32** and **C34** were extended outside the gorge of AChE, supporting the feature, for the AChE-induced Aβ aggregation inhibition. The orientation pose of ligands **C31** and **C33** were reversed to that of **C30**, **C32** and **C34**. These ligands was found to be nicely accommodated in the gorge of AChE active site, in such a manner that the benzylamido moiety was noted to lean towards CAS. A T-shape edge-to-face π–π stacking interaction of, 3-halo phenyl ring of benzylamido moiety against Phe338 was observed specifically which helped in hydrogen bond interaction with the OH of Tyr124 inside the mid gorge. In this binding mode CAS and PAS both were well occupied by the ligand and hence it is in agreement with the mixed mode inhibition pattern of **C31** but however on the other hand compound **C33** disclosed only one hydrogen bond interaction between imino NH and Phe 295 (Fig. 31).

**Fig. 32:**
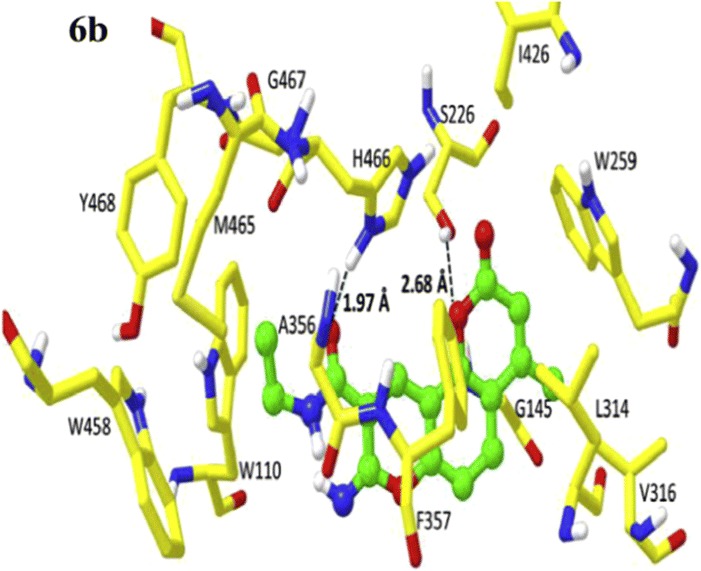
Docking pose of compounds **C22** at the binding site of BuChE [49]

The AChE is composed of aromatic amino acid residues like Tyr72, Tyr124, Trp286, Phe295, Phe297 and Tyr337 and BuChE is composed of aliphatic amino acid residues like Asn68, Gln119, Ala277, Leu286, Val288, and Ala328. This is the cause factor behind the poor ability of BuChE to form π–π stacking interactions as compared to AChE. Docking studies of the most active anti-BuChE compound (**C22**) revealed that it can effectively fit into the gorge of BuChE active site which makes it a more potent BuChE inhibitor than other member of the series. Its favourable orientation within the gorge accounts for higher affinity towards BuChE and effective π–π stacking interaction between Phe357 and tricyclic coumarin moiety. In addition, two hydrogen bonds were reported with the amino acids of the catalytic triad. One between the oxygen of coumarin and OH of Ser226 and the other between the carbonyl of coumarin and NH of His446. A CH-π interaction between Trp110 and *N*-ethyl group of the amide part was also reported. Moreover hydrophobic interactions with Trp110, Leu313, Leu314, Val316, Ala356, Ile426, Trp458, Met465, and Tyr468 aliphatic residues were also reported (Fig. 32) [49].

**Fig. 33:**
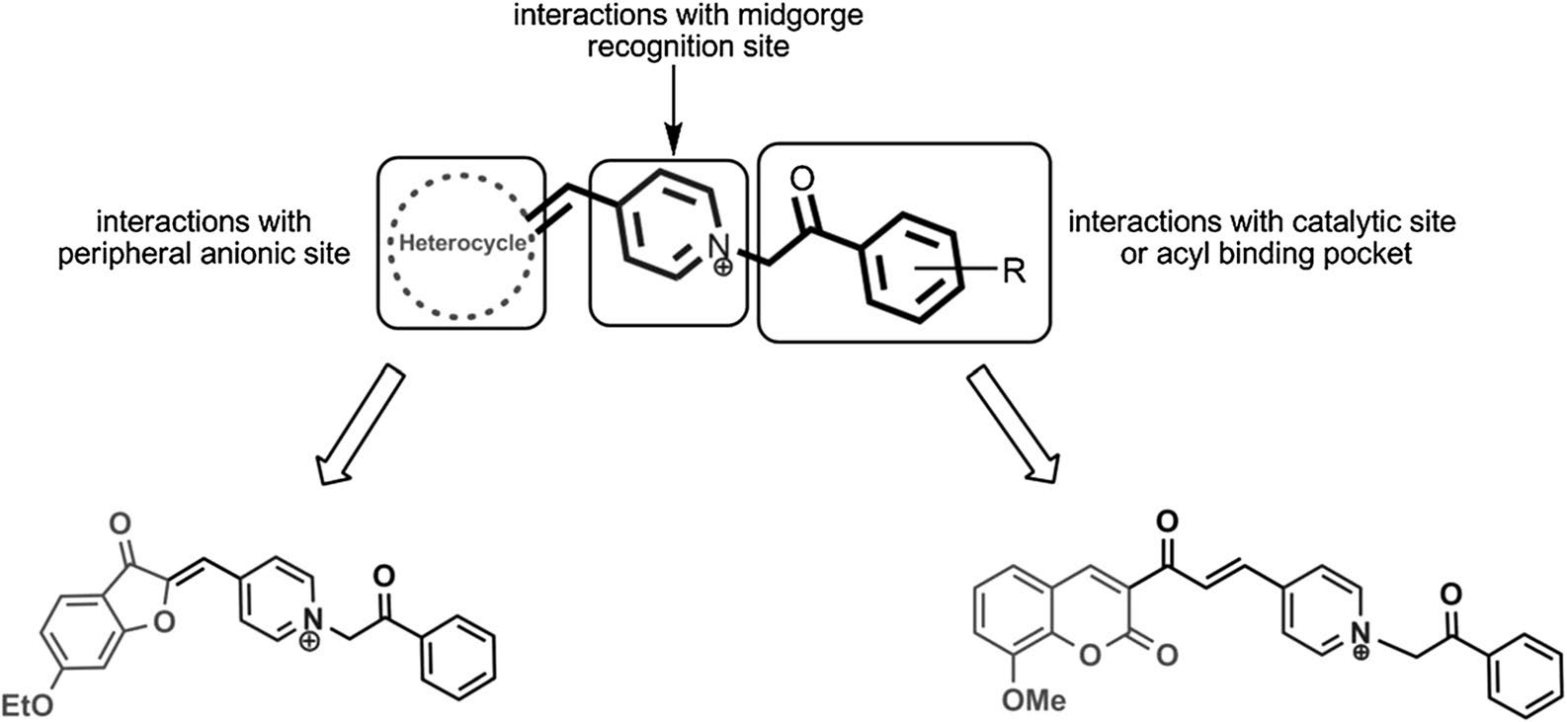
Designed structures as potential inhibitors of AChE and BuChE [50]

Alipour et al. [50] synthesized 3-coumaranone and coumarin derivatives encompassing the phenacyl pyridinium moiety as dual inhibitors of acetyl and butyrylcholinesterase. The obtained docking results suggested that all the synthesized compounds were dual binding inhibitors of AChE in the micromolar range with slightly different interactions with the receptor. Interestingly it was reported that 3-coumaranone derivatives were more good AChE inhibitors than the coumarin derivatives (Fig. 33).

**Fig. 34:**
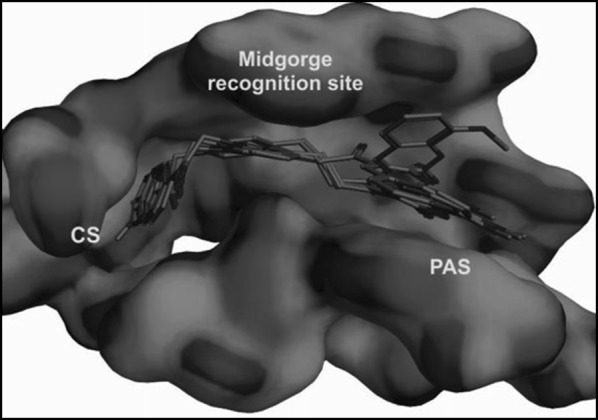
Superimpositions of the best-docked poses of coumarin analogues in the AChE binding site [50]

Taking into account the results obtained from the coumarin derivatives, it was revealed that coumarin analogues were protruded towards the CS and the longer molecules were reported to stretch out of the gorge and found interacting with Trp84 at the proximity of the CS via π–π interaction (Fig. 34).

**Fig. 35:**
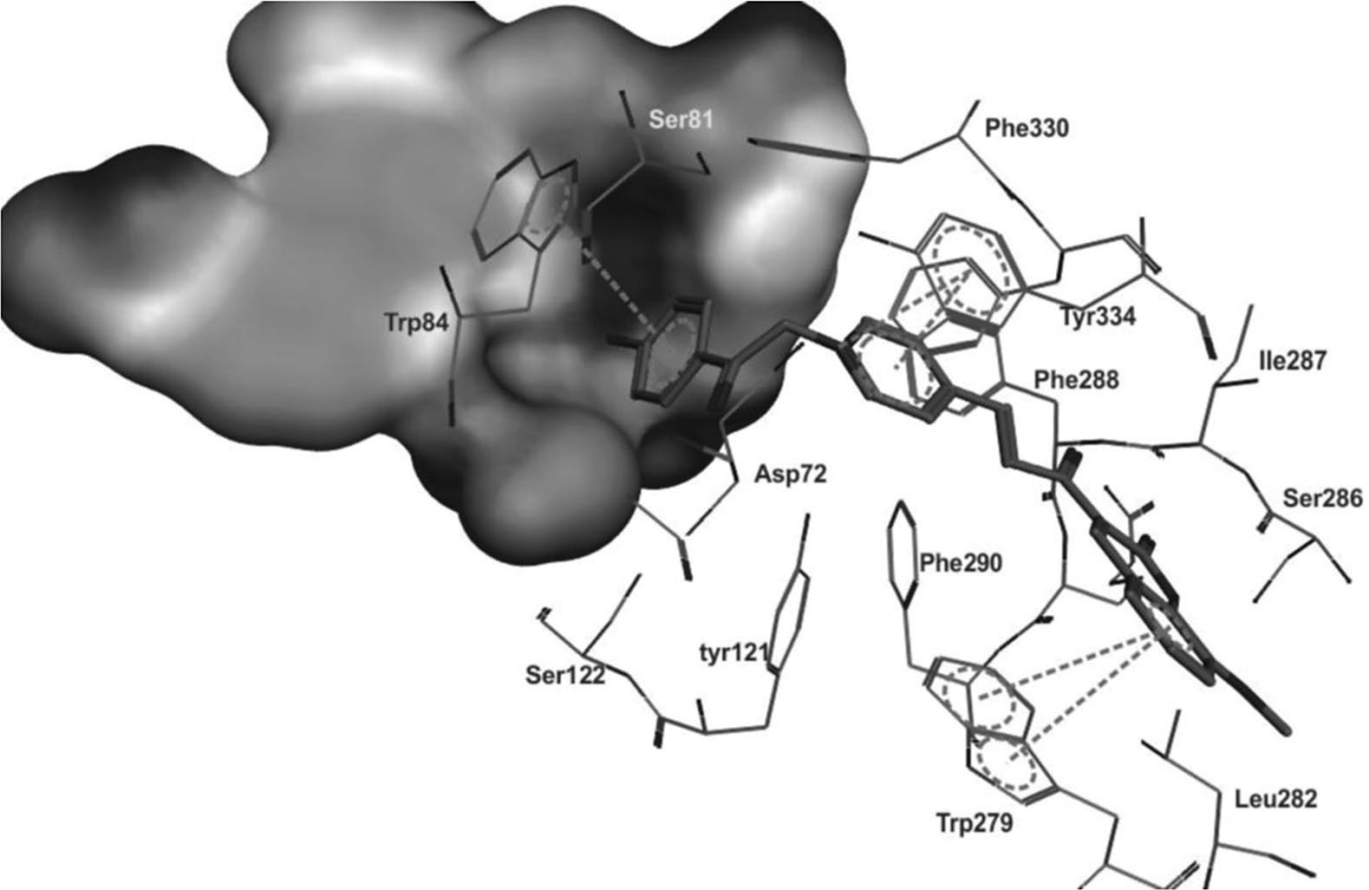
Interaction of compound **C40** docked into the binding site of AChE. The hydrophobic bromo substituent of **C40** is oriented toward a hydrophilic pocket of active site [50]

In terms of structure activity relationship the order of activity was immensely affected by the electron withdrawing nature and lipophilicity of the substituents present on the penacyl pendant which followed the activity order as: 4-F ≈ 4-H > 4-CH3 > 4-OCH3 > 4-Br (**C36**, **C37**, **C38**, **C39**, **C40**). The orientation of the phenacyl pendant is directed towards the hydrophilic pocket comprised of Gly441, Glu199, His440, and Tyr130. Therefore the substituents with strong lipophilic nature at para position of phenacyl ring (4-Br) are imperfectly accommodated within the lipophilic pocket, making the analogue a slow AChE inhibitor (**C40**) (Fig. 35). Electron withdrawing substituents which were low in lipophilic character (4-F) were found to be well tolerated at para position of phenacyl ring as reported for compound **C36**. On the other hand, substituents with good electron donating nature (4-CH_3_, 4-OCH_3_) at para position were reported to diminish AChE activity by weakening the π–π interaction with Trp84 (**C38**, **C39**). Moreover, coumarin analogues were found to be slow butyrylcholinesterase inhibitor as compared to the inhibition of AChE except for the derivative **C40** with methyl substituent at para position displaying the IC_50_ value of 8 μM [50].

**Fig. 36:**
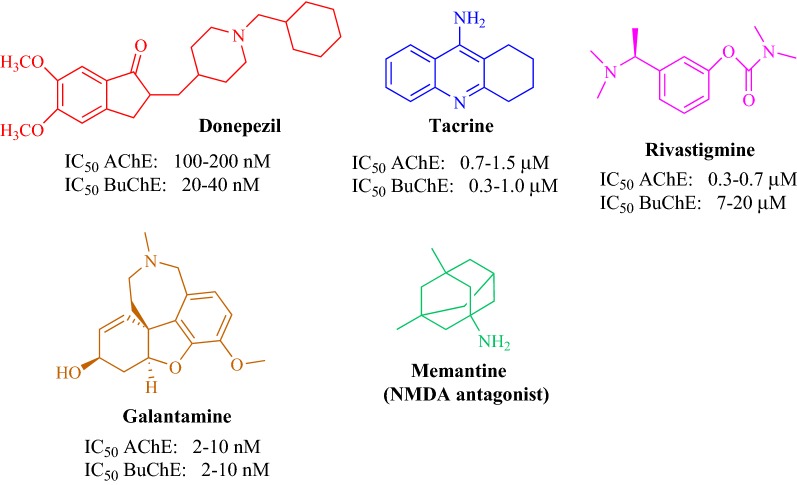
Marketed anti-AD drugs. For cholinergic drugs, inhibitory activity ranges on AChE and BuChE are reported

**Fig. 37:**
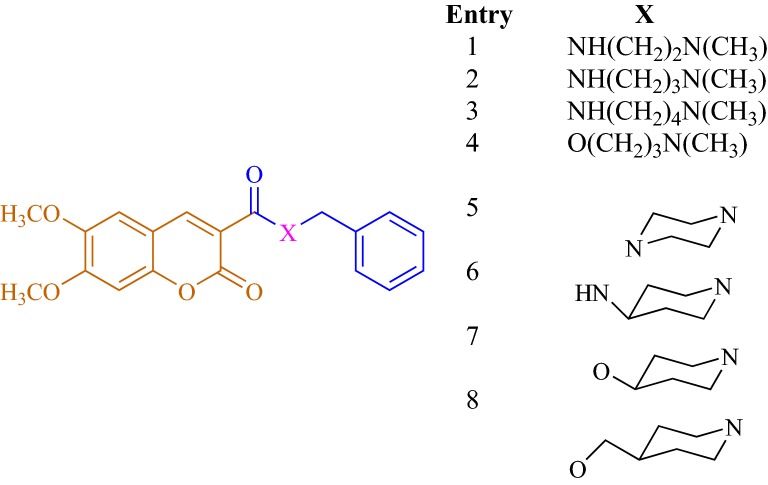
Coumarin alkylamines **C41a**–**C41h** (entry 1–8)

**Fig. 38:**
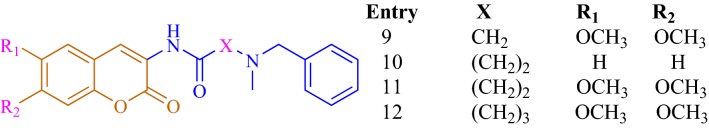
Coumarin alkylamines **C41i**–**C41l** (entry 9–12)

**Fig. 39:**
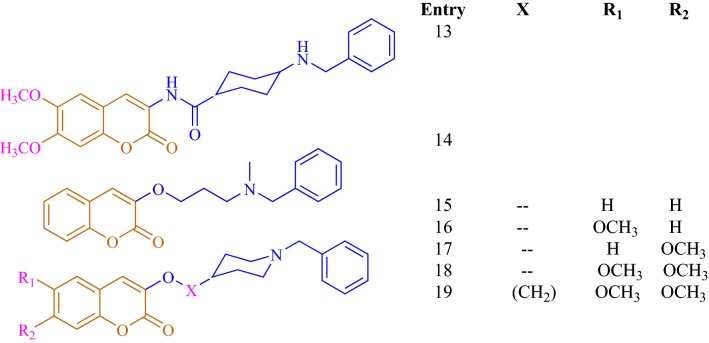
Coumarin alkylamines **C41m**–**C41s** (entry 13–19)

Catto et al. [51] reported a series of coumarin alkylamines (**C41a**–**C41s**; Entry 1–19), matching the structural determinants to one of the most commonly used standard marketing anti-AChE drug donepezil, as potent and dual binding site inhibitors for acetylcholinesterase (Figs. 36, 37, 38 and 39). Among the synthesized series, the 6,7-dimethoxycoumarin analogues which were composed of protonatable benzylamino group and linked via suitable linker to position-3 were found to be the most active AChE inhibitor as well as high selective over BuChE. It’s worth to mention here that the extent of inhibition was influenced by the length and the shape of the spacer and by the presence of methoxy substituents on the coumarin nucleus. The most active compound **C41m** (entry 13) with IC_50_ value of 7.6 nM was found to be the dual inhibitor revealing binding at the CS and PAS of AChE enzyme.

**Fig. 40:**
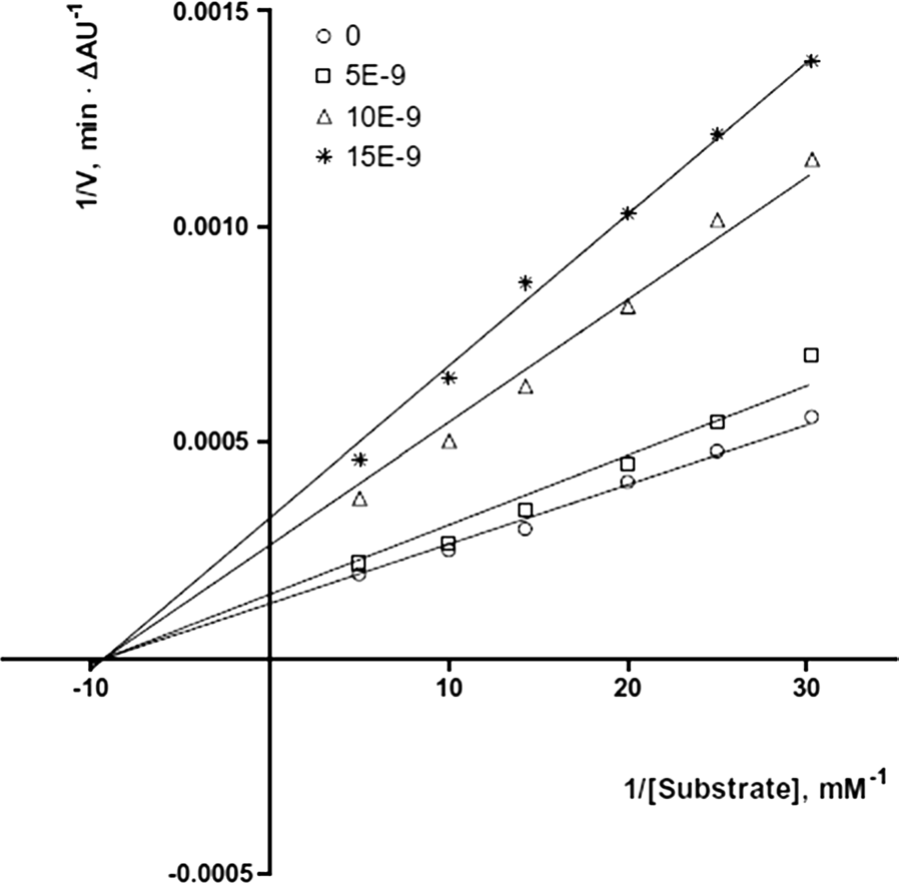
Lineweaver–Burk plot of inhibition kinetics of **C41m** (entry 13): reciprocals of enzyme activity (bovine AChE) vs reciprocals of substrate (*S*-acetylthiocholine) concentration in the presence of different concentrations (0–15 nM) of inhibitor **C41m** (entry 13) [51]

In order to get the insight behaviour of the coumarin analogues anti-ChE activities were performed on AChE collected from bovine erythrocytes and BuChE collected from equine serum by Ellman’s methodology. Results suggested that all the compounds except aminoethers **C41n, C41o** and **C41p** (entry 14, 15 and 16) were selective AChE inhibitor as compared to BuChE with IC_50_ values in the range of 66 μM for compound **C41j** (entry 10) to 7.6 nM for the cis-3-amino-cyclohexanecarboxylic acid derivative (**C41m**) whose IC_50_ value was the highest and very close to the reference donepezil, with IC_50_ 4.2 nM. Additionally compounds **C41a**–**C41c** (entry 1–3) also showed an increase in potency with the elongation of their polymethylene linker. Compound **C41a** (9.0 μM) was the less active compound from the amide series and the IC_50_ was noted to decrease on moving to the least compound **C41b** (86 nM) whilst compound **C41c** was the most active compound (21 nM). Compound **C41l** (entry 12), the amide analogue of compound **C41b**, was reported with the four-fold drop of inhibitory activity due to the presence of inverted amide function. On the other hand compound **C41i** (entry 9) with short methylene linker and aminoether compound **C41n** (entry 14) with longer triethylene linker was reported to exhibit low inhibition. The absence of two-methoxy group in positions 6 and 7 of the coumarin ring might the cause of this decrease AChE affinity and a total loss of AChE/BChE selectivity for compound **C41n**. The closely related compound **C41n** (IC_50_ = 4.5 μM, entry 14) and **C41o** (IC_50_ = 4.5 μM, entry 15) were found to be equally potent anti-AChE while the monomethoxy substituted analogues **C41p** (IC_50_ = 7.4 μM, entry 16) and **C41q** (IC_50_ = 12 μM, entry 17) were noted to be weaker but displaying moderate selectivity over BuChE. Moreover 6,7-dimethoxy analogue **C41r** (IC_50_ = 1.5 μM, entry 18) expressed low inhibitory property but good BuChE selectivity. It was noted that the elongation of spacer with a methylene group, from derivative **C41r** to derivative **C41s** (IC_50_ = 21 nM, entry 19) increased its potency as well as increased its BuChE/AChE selectivity ratio by 186. The inhibition mechanism of the enzyme AChE by the most active compound **C41m** was also interpreted by the kinetic studies and the results are displayed via the Lineweaver–Burk plot (Fig. 40). The inhibition constant Ki, was equal to 8.6 ± 1.5 nM. The plot displayed reversible and mixed type inhibition model, which was in accordance with the dual binding site model of interaction with the enzyme AChE [51].

**Fig. 41:**
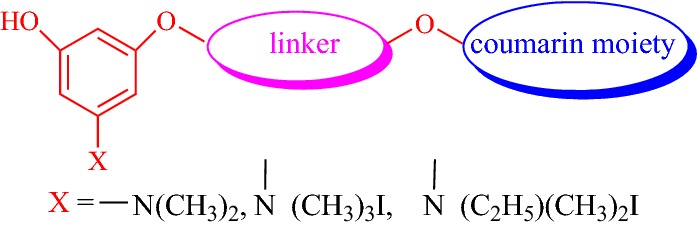
General structure of the synthesized coumarin derivatives

Researcher Leonardo Pisani from the above mentioned research group of Catto et al. also designed and synthesized another large series of coumarin derivatives. Pisani et al. linked the coumarin ring to 3-hydroxy-*N*,*N*-dimethylanilino or 3-hydroxy-*N*,*N*,*N*-trialkylbenzaminium moieties via a suitable spacer and subjected the derivatives for their further evaluation as acetylcholinesterase (AChE) and butyrylcholinesterase (BuChE) inhibitors (Fig. 41).

**Fig. 42:**
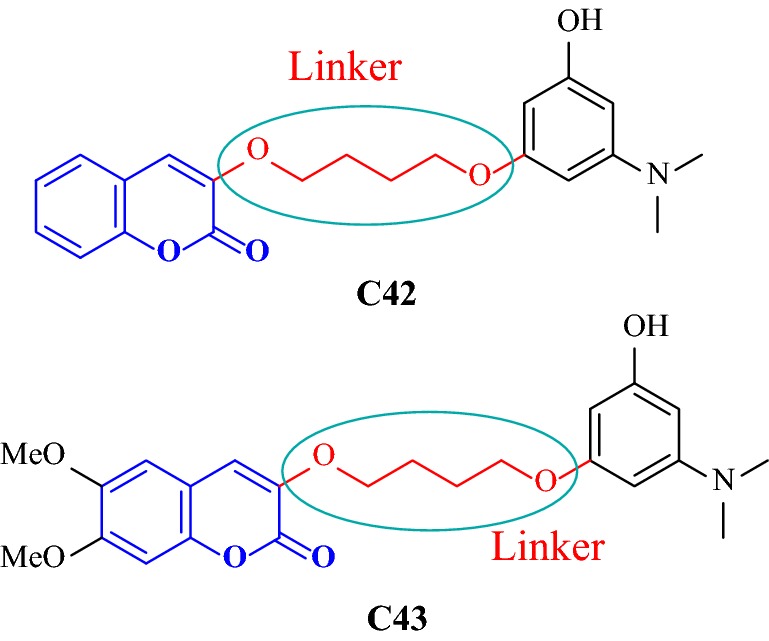
Molecular structures of dimethylamino coumarin derivatives **C42** and **C43**

Among all the derivatives the one with two methoxy groups at position 6 and 7 of the coumarin ring viz 3-{4-[3-(Dimethylamino)-5-hydroxyphenoxy]butoxy}-6,7-dimethoxy-2H-chromen-2-one (**C43**), was dramatically found to be the most potent anti-AChE displaying IC_50_ 0.236 nM even higher than the chosen reference donepezil. Compound **C43** and donepezil both were reported to exhibit higher IC_50_ values towards human AChE (IC_50_ = 39.7 nM) as compared to bovine AChE (IC_50_ = 26.0 nM). Even so compound **C43** was an excellent AChE inhibitor, unexpectedly its affinity profile towards BuChE was quite poor (IC_50_ = 71,000 nM). Same ways the activities of all the derivatives was checked and the BuChE and AChE inhibition data suggested that these compounds could be regarded as selective, sub-micromolar AChE inhibitors rather than potential BuChE inhibitors. Therefore, the surprisingly high AChE binding affinity observed in moving from inhibitor **C42**, (3-{4-[3-(Dimethylamino)-5-hydroxyphenoxy]butoxy}-2H-chromen-2-one) to **C43** (IC_50_ = 143 nM), which differs only in the 6,7-dimethoxy substitution at the coumarin ring was tried to investigated by molecular docking (Fig. 42). But unfortunately docking results were incapable to interpret this unexpected rise in AChE binding affinity for inhibitor **C43**. Hence, the MD study was performed.

**Fig. 43:**
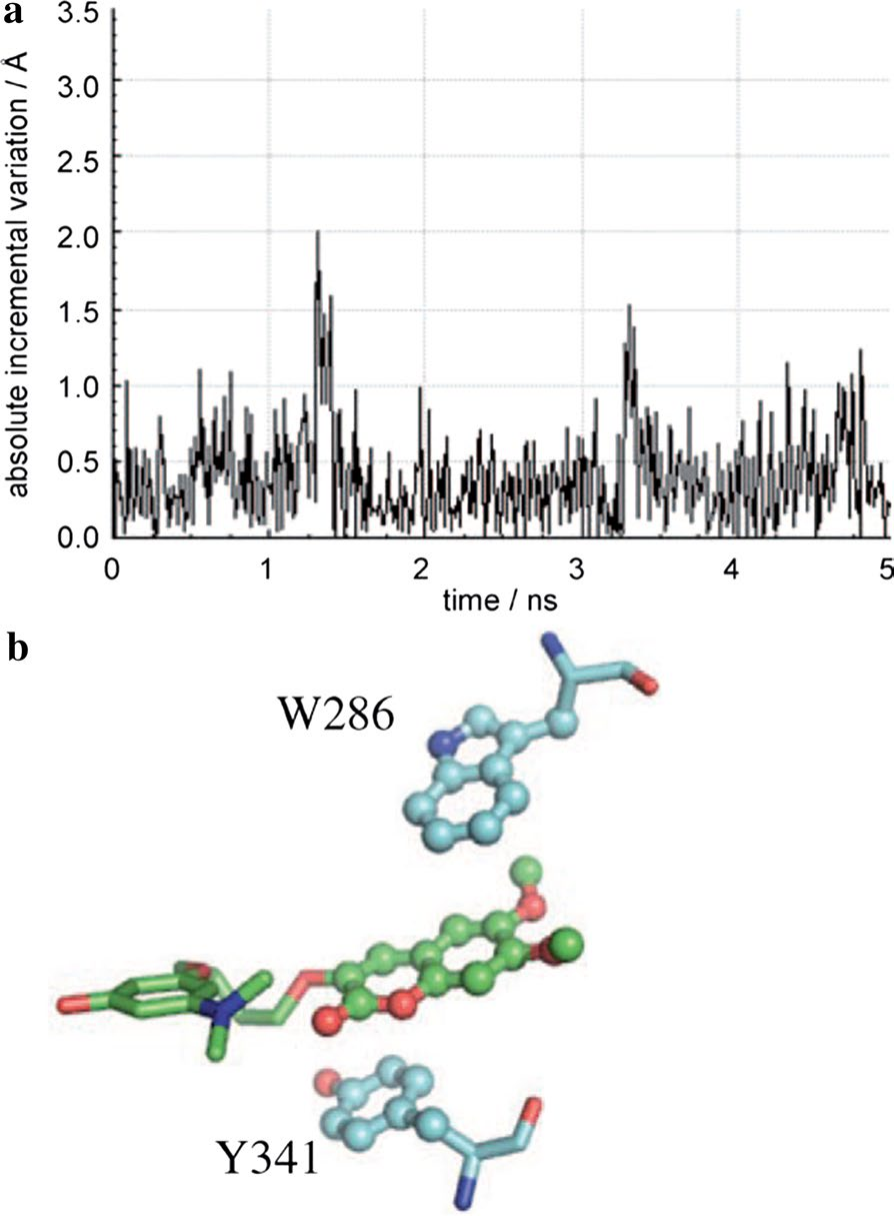
**a** Plot showing the absolute incremental variation (from the initial conformations obtained from molecular docking) of the half-sum of distances calculated from the centres of mass of residues W286 and Y341, and the centroid of the coumarin ring of inhibitor **C43** over 5 ns MD simulations. **b** A representative sandwich-like binding conformation of C43 taken after 5 ns MD simulations [52]

The top-scored docking poses of these two reference compounds were took into account for the MD studies to determine the variation in structure in the AChE complexes over time. Taking about the inhibitor **C43** first, an overall sandwich-like orientation was maintained throughout the MD procedure due to stabilizing π–π stacking interaction which was established because of the two methoxy substituents which stabilized the coumarin moiety into the PAS at the place whose large area was exposed to the solvent. These two methoxy groups which were facing the solvent, enabled inhibitor **C42**, to sandwiched its coumarin moiety into the open slot between indolic and phenolic rings of Trp286 and Tyr341, respectively (Fig. 43). In the primary binding site of the enzyme AChE, the inhibitor **C43** was reported to orthogonally orient its 3-hydroxy-*N*,*N*-dimethylanilino fragment to Trp86 and thus forming network of hydrogen bonds with the two solvent water molecules through their *N*,*N*-dimethylamino and phenolic group. On the other hand, inhibitor **C42** was reported to be smarter to dive deeper in the gorge of AChE in order to diminish the exposure to the solvent. However for **C42** the sandwich interaction with Trp286 and Tyr341 was not reported because the coumarin ring was found to form a T-shaped orthogonal π–π interaction with Trp286 or, a parallel -π interaction with Tyr341 [52].

To interpret the pronounced molecular enzymatic selectivity a comparison between AChE and BuChE top-scored solutions of inhibitor **C43** was done. The results revealed that, 3-hydroxy-*N*,*N*-dimethylanilino moiety was orthogonally oriented to Trp82, and its phenolic and ethereal oxygen atoms were reported to interact with Glu197 and Ser198 (BuChE numbering) of the catalytic triad by hydrogen bonds. Moreover, due to absebnce of PAS in BuChE possibility of π–π interaction is not present as for AChE. Hence, the AChE/BuChE selectivity could be expressed via different molecular binding conformations observed and it is supported by the different energy scores (i.e. 57.27 Vs 50.84 kJ/mol).

**Fig. 44:**
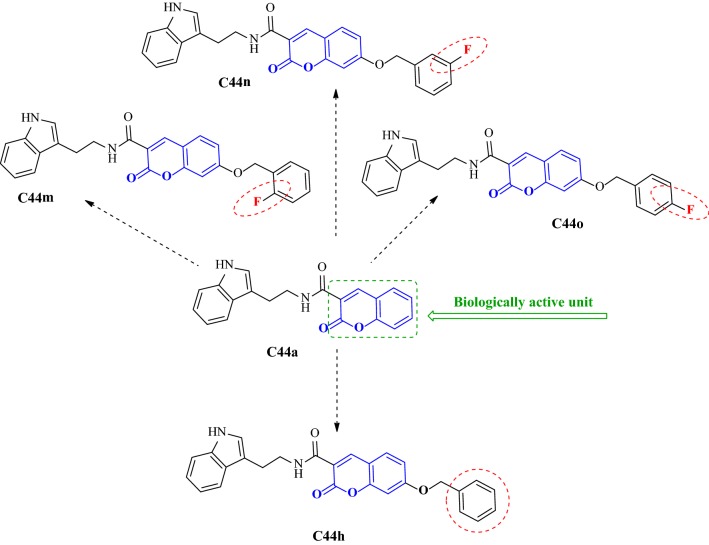
Comparative structure of coumarin carboxamides **44a**, **C44m**, **C44n**, **C44o** and **C44h**

Ghanei-Nasab et al. [53] synthesized a series (**C44a**–**C44o**) of *N*-(2-(1H-indol-3-yl)ethyl)-2-oxo-2H-chromene-3-carboxamides bearing tryptamine moiety and tested them against AChE and BuChE. The SAR study revealed few facts about the presence of halogen atoms. One or two chlorine atoms on the benzyl moiety tend to decrease the anti-AChE property of the compounds whereas the flouro atom at ortho or meta position on the benzyl moiety was acting moderately and similarly (compounds **C44m**, **C44n**), but it was found to show improved activity against AChE when at para position (**C44o**). The most active compound **C44o** with flouro at para position of benzyl moiety was 15-fold more stronger (IC_50_ = 0.016 μM) than **C44m** and **C44n** and 9-times superior to its benzyl analogue **C44h**. The activity results for BuChE revealed that these compounds were mild or not active and the inhibitory activity of all compounds against AChE was higher over BuChE. The most potent* O*-benzyl derivative, **C44h** was found display IC_50_ value of 16.2 μM. It was 3-fold more potent than analogue **C44a** (Fig. 44).

**Fig. 45:**
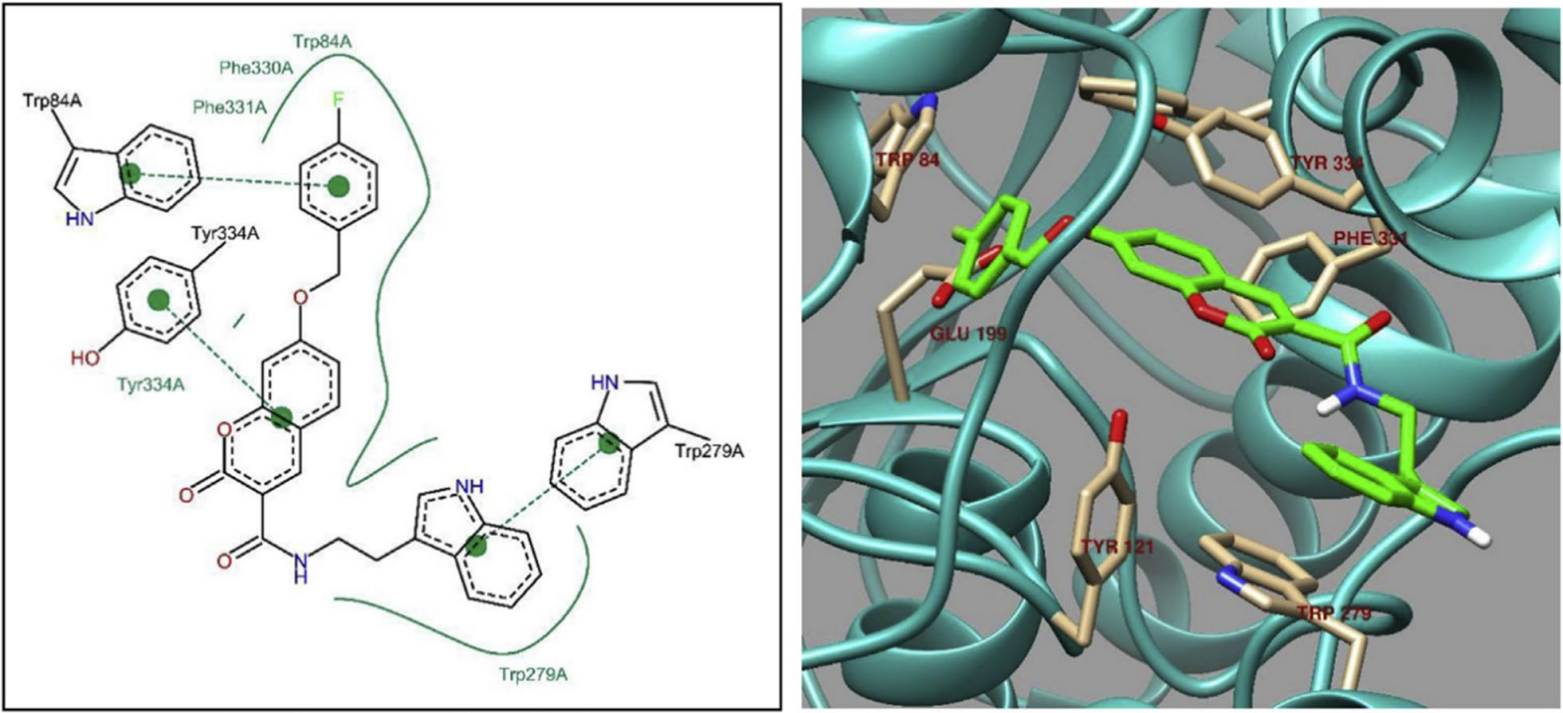
2D (left) and 3D (right) representation of interactions of compound **C44o** in the active site of AChE [53]

In order to get the insight of the binding modes the most active derivative **C44o** was laid across the active site and interestingly occupied PAS and CAS, both (Fig. 45). Its 4-fluorobenzyl moiety was found to be oriented towards the bottom of the active site and formed a face-to-face π–π stacking with Trp84. π–π stacking between coumarin ring and Tyr334 was also observed and indole ring was also found to interact with Trp279 of the PAS via π–π interaction [53].

**Fig. 46:**
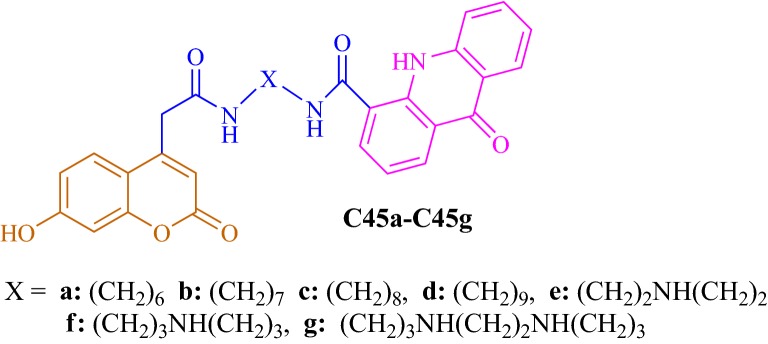
Structures of synthesized of acridine-coumarin hybrids **C45a**–**C45g**

Hamulakova et al. [54] designed and synthesized acridine-coumarin hybrids and check their in vitro acetylcholinesterase inhibitory activity against human erythrocytes and butyrylcholinesterase inhibitory activity against human plasmatic butyrylcholinesterase against tacrine and the reference drug 7-MEOTA. Among all the compounds tested, **C45b** with 7 methylene groups exhibited the highest acetylcholinesterase inhibitory activity, with IC_50_ value of 5.85 μM and with potency 3-times stronger when compared to the reference with IC_50_ of 15 μM. On the other hand, compound **C45c** and **C45f** was found to be most potent against hBuChE with the IC_50_ value of 32.53 μM and 43.40 μM (Fig. 46). Molecular modelling studies were performed to predict the binding modes of compounds **C45b**, along with **C45c** and **C45f** with hAChE/hBuChE.

**Fig. 47:**
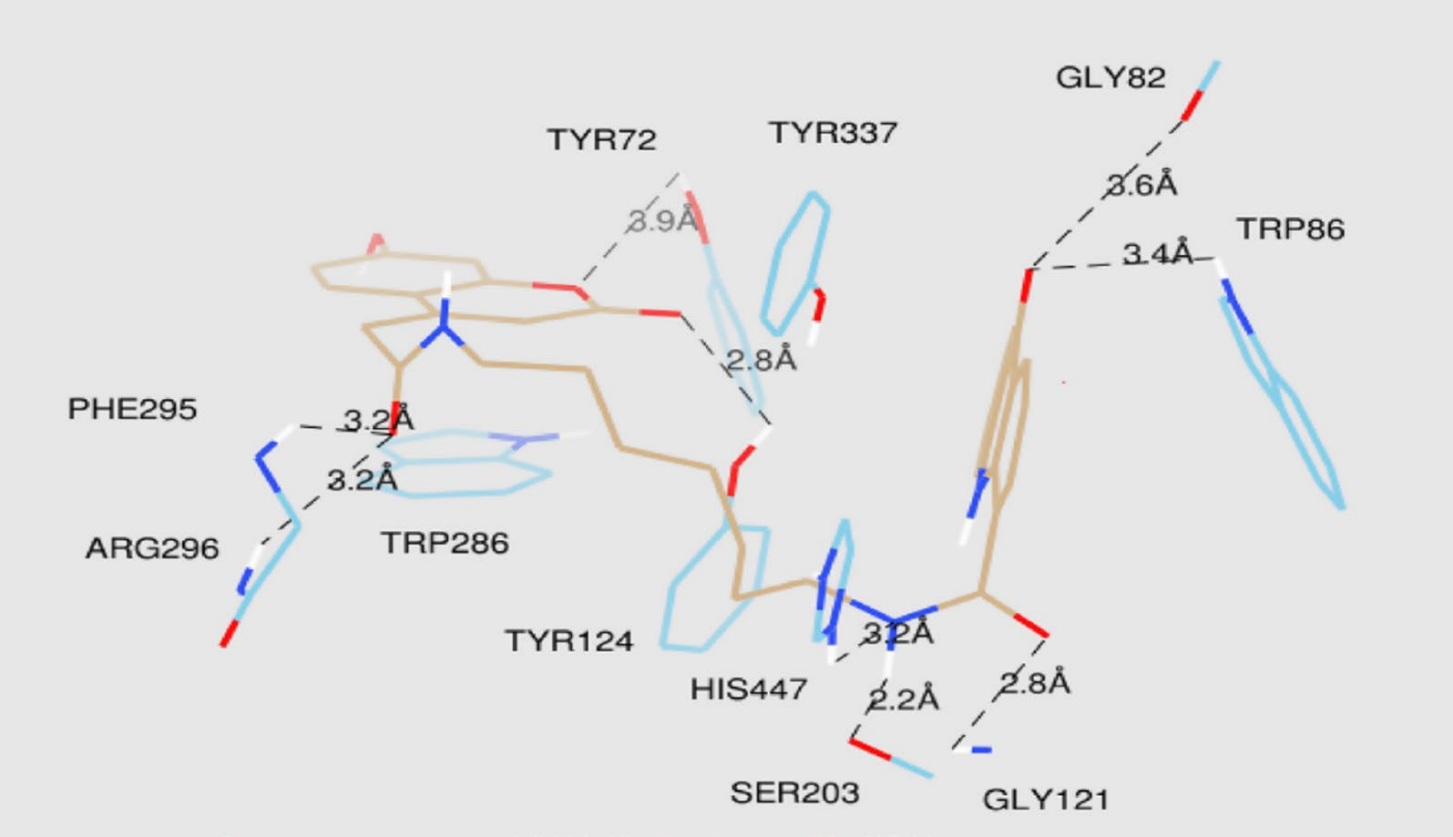
Top-score docking pose of derivative **C45a** depicting its putative hydrogen bonds formed with amino acid residues in the active-site gorge of hAChE [54]

**Fig. 48:**
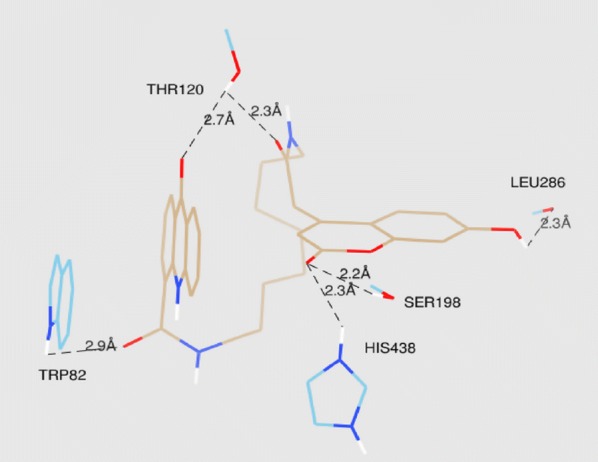
Top-score docking pose of derivative **C45b** depicting its putative hydrogen bonds formed with amino acid residues in the active-site gorge of hBuChE [54]

**Fig. 49:**
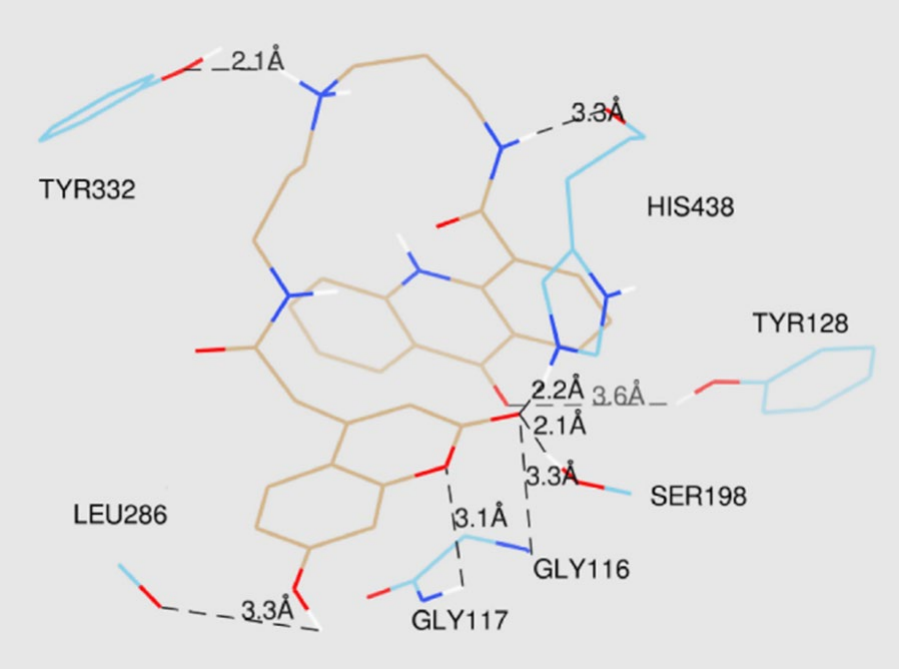
Top-score docking pose of derivative **C45f** depicting its putative hydrogen bonds formed with amino acid residues in the active-site gorge of hBuChE [54]

The lowest energy binding pose of derivative **C45b** with hAChE is depicted in Fig. 47. π–π binding interactions between coumarin core and the aromatic residue of Trp286 in the PAS of the enzyme was reported. Interaction of Ser302 and His447 of amino acids with the amide group of the acridone core was also reported. Additionally, interaction between amino acids of the active site of the enzyme and ligand was also reported. The direct interaction of compound **C45c** with the catalytic triad via His438 andSer198 was clearly reported in the active site of the enzyme hBuChE (Fig. 48). Moreover hydrogen bonds and π–π interactions between amino acids of the catalytic cavity and **C45c** was also reported. The pose with the lowest binding energy for derivative **C45f** with hBuChE is depicted in Fig. 49. Direct interactions weith the residues His438 andSer198 and one additional intermolecular interaction between the catalytic cavity of the enzyme BuChE and derivative was also reported. The docking results suggests that acridine seems to be a possible substitute for tacrine in the family of dual binding site inhibitors [54].

**Fig. 50:**
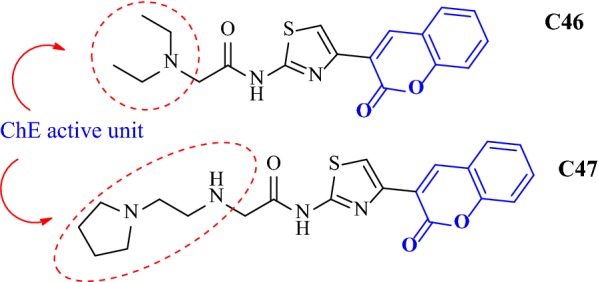
Molecular structure of the most anti-AChE (**C46**) and anti-BuChE (**C47**) thiourea derivatives

Sonmez et al. [55] designed and synthesized coumaryl-thiazole derivatives with the acetamide moiety as a linker between the alkyl chains and/or the heterocycle and tested their potency against AD [55]. Docking studies were performed to check the binding profile of coumarin derivatives into the active site of AChE and BuChE enzymes against galantamine. Both these cholinesterases (AChE and BuChE) are similar in structure and also 65% of the amino acid sequences for both of these are similar [56]. The basic difference, is the presence of aromatic amino acid in AChE over BuChE which possess aliphatic amino acids, making them both selective, against different inhibitors of the two enzymes [57]. From all the thiazole derivatives compound **C46**, 2-(diethylamino)-*N*-(4-(2-oxo-2H-chromen-3-yl)thiazol-2-yl)acetamide (IC_50_ = 43 nM) was the most potent AChE inhibitor with a selectivity index of 4151.16 over BuChE and 56-fold more stronger than the standard galantamine (IC_50_ = 2.4 μM). The BuChE activity of most of the compounds was lesser than their AChE activity except for compound **C47** that exhibited the strongest inhibition against BuChE with an IC_50_ value of 2.35 μM, which was 2 and 7.5 fold more than the standards donepezil (IC_50_ = 4.66 μM) and galantamine (IC_50_ = 17.38 μM) (Fig. 50).

**Fig. 51:**
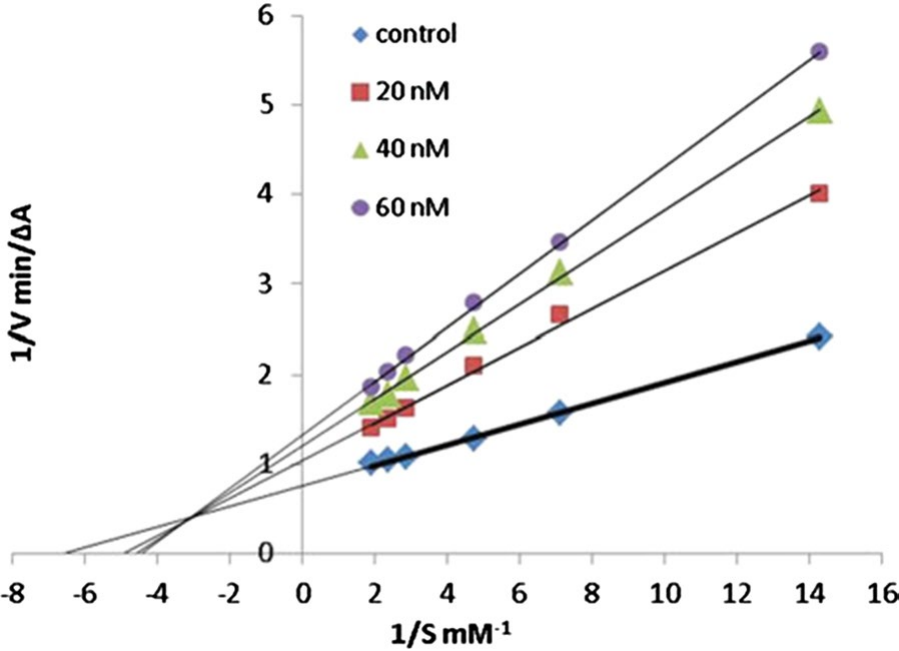
Kinetic study on the mechanism of AChE inhibition by compound **C46**. Overlaid Lineweaver–Burk reciprocal plots of AChE initial velocity at increasing substrate concentration (0.05–0.50 mM) in the absence of inhibitor and in the presences of different concentrations of **C46** are shown [55]

Kinetic enzymatic study was carried out in order to explore the mechanism of inhibition of compound **C46** with the enzyme AChE. The Lineweaver–Burk plot (Fig. 51) displayed increased slopes (decreased Vmax) and intercepts (higher Km) at higher inhibitor concentration. This pattern indicated a mixed-type inhibition and hence it was concluded that compound **C46** was able to bind to CAS, PAS and the catalytic triad of AChE. The inhibition constant Ki, was equal to 31 nM [55].

**Fig. 52:**
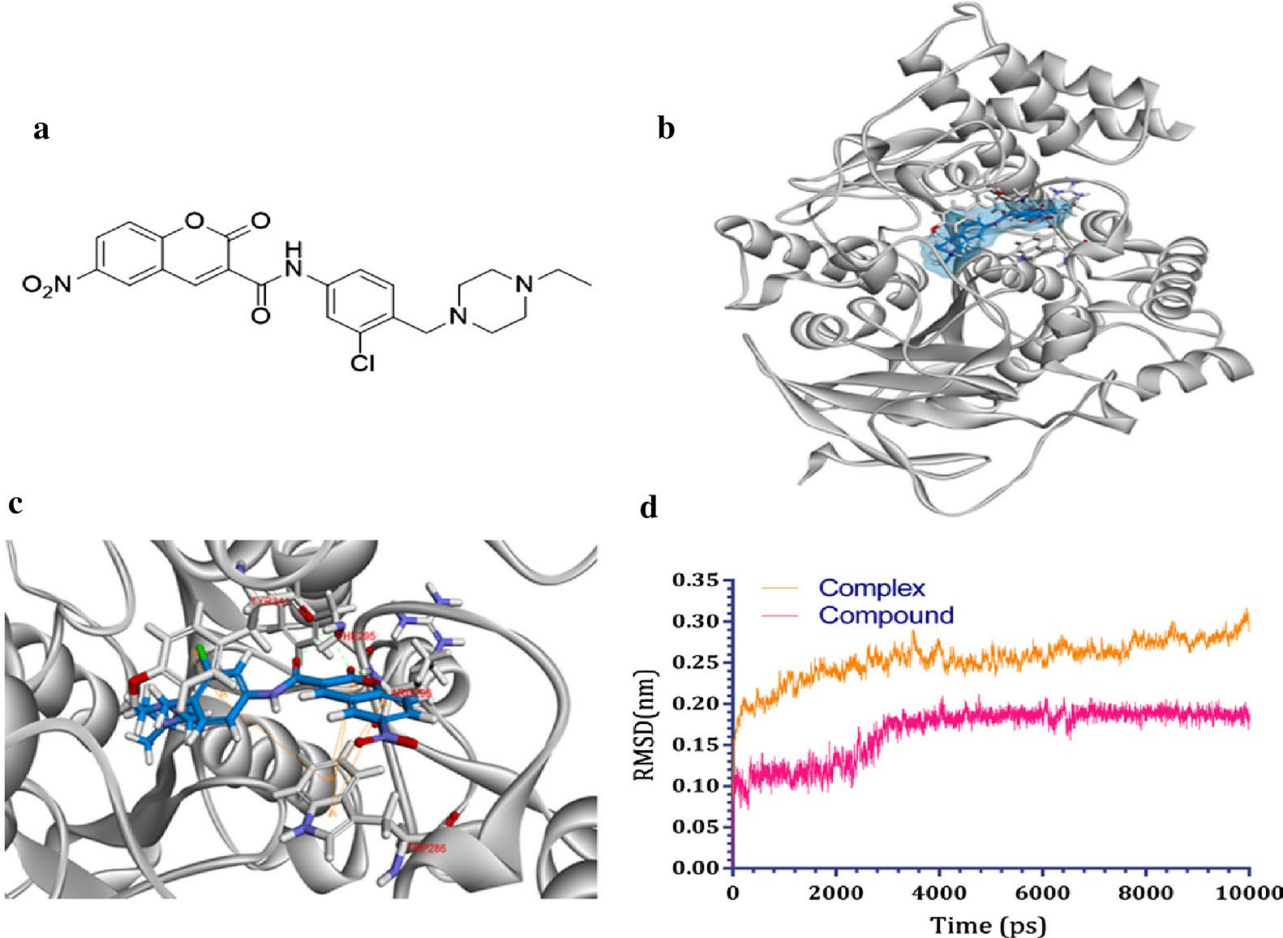
Molecular modelling, docking and molecular dynamics (MD) simulations of AChE targeting compound **C48**-AChE. **a** Molecular structure of compound **C48**. **b** Molecular docking of compound **C48**. **c** The interactions of compound **C48** and active pocket. **d** Molecular dynamics of compound **C48**-AChE complex [58]

Yao et al. [58] designed and synthesized coumarin derivatives containing piperazine ring and tested them for their AChE and BuChE inhibitory activity against two standards, huperzine A and Iso-OMPA. Results suggested that these compounds display significant inhibition for AChE over BuChE and compound *N*-(3-chloro-4-((4-ethylpiperazin-1-yl)methyl)phenyl)-6-nitro-2-oxo-2H-chromene-3-carboxamide (**C48**) was reported to be the most potent AChE inhibitor with IC_50_ value of 34 nM. The docking results revealed that compound **C48** was able to bind to AChE, with PAS of AChE via Trp286 and Arg296 residues and Trp286 was the main residue in ligand recognition as it was found to bind the aromatic rings of coumarin by four π–π stacking. The OC=O group of the coumarin nucleus was reported to interact with Phe295 and Arg296 by two hydrogen bonds. In addition, the central part of amide bond formed hydrogen bond with Phe295 in the deep aromatic narrow gorge. Moreover, the aniline moiety of **C48** formed two π–π stacking with residues Tyr341 and Trp286, whereas its ethylpiperazine moiety covered the CAS via hydrophobic interactions with Phe330 and Trp84, respectively. These results depicted the binding stability of C48 to AChE. In order to get the insight about the stability of the complex compound **C48**-AChE, 3 ns MD simulations were successfully performed on compound **C48**-AChE complex, and the observations indicated well behaved systems (Fig. 52) [58].

## Coumarin analogues as MAO inhibitors

### Introduction to MAO and its sub-isoforms MAO-A and MAO-B

Monoamine oxidase is a flavin adenine dinucleotide (FAD) containing enzyme, which is tightly bound to the outer membrane of the mitochondria of the neuronal cells, glial cells and to other cells [59]. It works as a catalyst in the oxidative deamination of monoamines either from endogenous sources or from exogenous sources. Therefore, it affects the concentrations of neurotransmitter amines as well as many xenobiotic ones [60]. It is also responsible for the biotransformation of a Parkinson producing neurotoxin i.e. 1-methyl-4-phenyl-1,2,3,6-tetrahydropyridine (MPTP) into 1-methyl-4-phenylpyridinium [61–63]. It is reported to also inhibit MAO activity-suppressed cell death hence actively participating in the apoptosis process [64]. MAO exists in two isoforms, which are MAO-A and MAO-B which differ from each other in quite a number of factors such as different inhibitor, amino acid sequence, and substrate specificities (Table 1).

**Table 1:** Main difference between the two isoforms MAO-A and MAO-B

MAO-A	MAO-B
It preferentially oxidizes nor-epinephrine and serotonin	Preferentially deaminates β-phenylethylamine and benzyl-amine [65, 66]
It is selectively inhibited by clorgyline	Selectively inhibited by **l**-deprenyl [65, 66]
Both the MAO isoforms have a different tissue distribution. Occurs in cathecholaminergic neurons as well as glia	MAO-B is predominant in the human brain, and is compartmentalized into different cell types. It occurs mainly in glial cells and serotoninergic neurons [67, 68]
MAO-A inhibitors have been used mostly in the treatment of mental disorders, in particular depression and anxiety [80, 81]	Used in the treatment of Parkinson’s disease and perhaps, Alzheimer’s disease [70, 82]

**Fig. 53:**
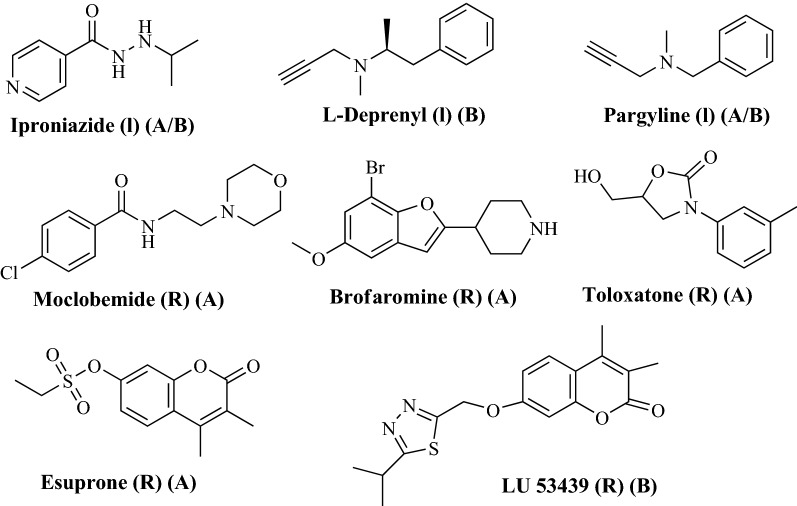
Reversible (R) and irreversible (I) MAO-A/B inhibitors

As compared to the nonselective-irreversible MAO-A and MAO-B inhibitors (MAO-Is) which were initially used with severe side effects for treating depression [69], the present selective and reversible inhibitors of MAO-A and MAO-B are rather more useful for treating depression, anxiety as well as coadjuvant agents in the treatment of Parkinson’s and Alzheimer’s disease [70]. Figure 53 depicts the molecular structure of few such nonselective-irreversible and selective-reversible inhibitors. Like in the category of nonselective-irreversible are iproniazide [71] and pargyline [72], in the selective-reversible category of MAO-A-Is are moclobemide, brofaromine, toloxatone [73–77] and esuprone [78] and in the selective-reversible category of MAO-B-Is is LU 53439 [79].

**Fig. 54:**
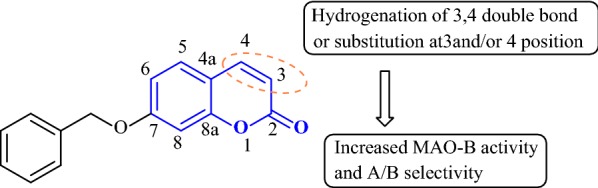
7-Benzyloxy coumarin **C49**

The reason behind so many different structure of MAO-Is is because of the fact that the active site of MAOs are still to be discovered. The structure of the active site of MAO-A is still unknown today, therefore there is a limitation in the design of new potent selective MAO inhibitors. Lately in the year 2002, Binda et al. have made an excellent effort in describing the experimental crystal structure of human MAO-B. These group of researchers crystallized the pargyline inhibitor covalently bounded to the N5 atom on the re side of the flavin moiety of the enzyme, identifying the residues bonding the catalytic cavity [83]. A step forward and a recent discovery in 2007 by the same author Binda et al. about the crystal structures of the two isoforms of human MAO were determined, which elucidates the mechanism underlying the selective interactions between these proteins and their ligands, probes the catalytic mechanism [84]. Among the various types of MAO-Is already discovered, the recent interest was diverted towards the (1H)-benzopyran structure and one of the most important example of benzopyran structure is the coumarin nucleus. It has been reported that the hydrogenation of 3,4-double bond and substitution at position 3 and/or 4 of the coumarin ring, modulated the MAO-B inhibitory property and A/B selectivity [85, 86]. Besides esuprone and LU53439 as discussed earlier (Fig. 53), recently 7-benzyloxy coumarin derivatives (**C49**) (Fig. 54) are gaining immense attention due to their inhibitory activity and selectivity towards MAO-B [87].

### Coumarin analogues as dual MAO-A and MAO-B inhibitors

**Fig. 55:**
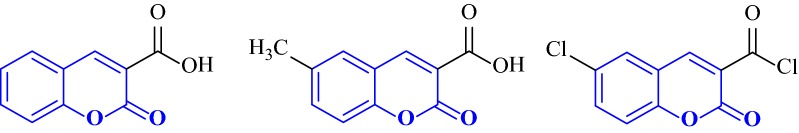
Molecular structure of compound **C50**, **C51** and **C52**

Chimenti et al. [88] designed and synthesized coumarin derivatives and studied their role in inhibition of MAOs via computation docking study. It was reported for the coumarin-3-carboxylic acids to be selective-reversible inhibitors of isoform MAO-B. Specifically compound **C50** was the most potent with IC_50_ value of 7.76 and selectivity index of 2.94 followed by compound **C51** with IC_50_ of 7.72 and SI of 2.80. For the 3-acyl chlorides coumarin derivatives, it was reported to be active, against both the isoforms MAO-A and MAO-B with compound **C52** being the strongest against MAO-B with IC_50_ value of 8.0 (Fig. 55).

**Fig. 56:**
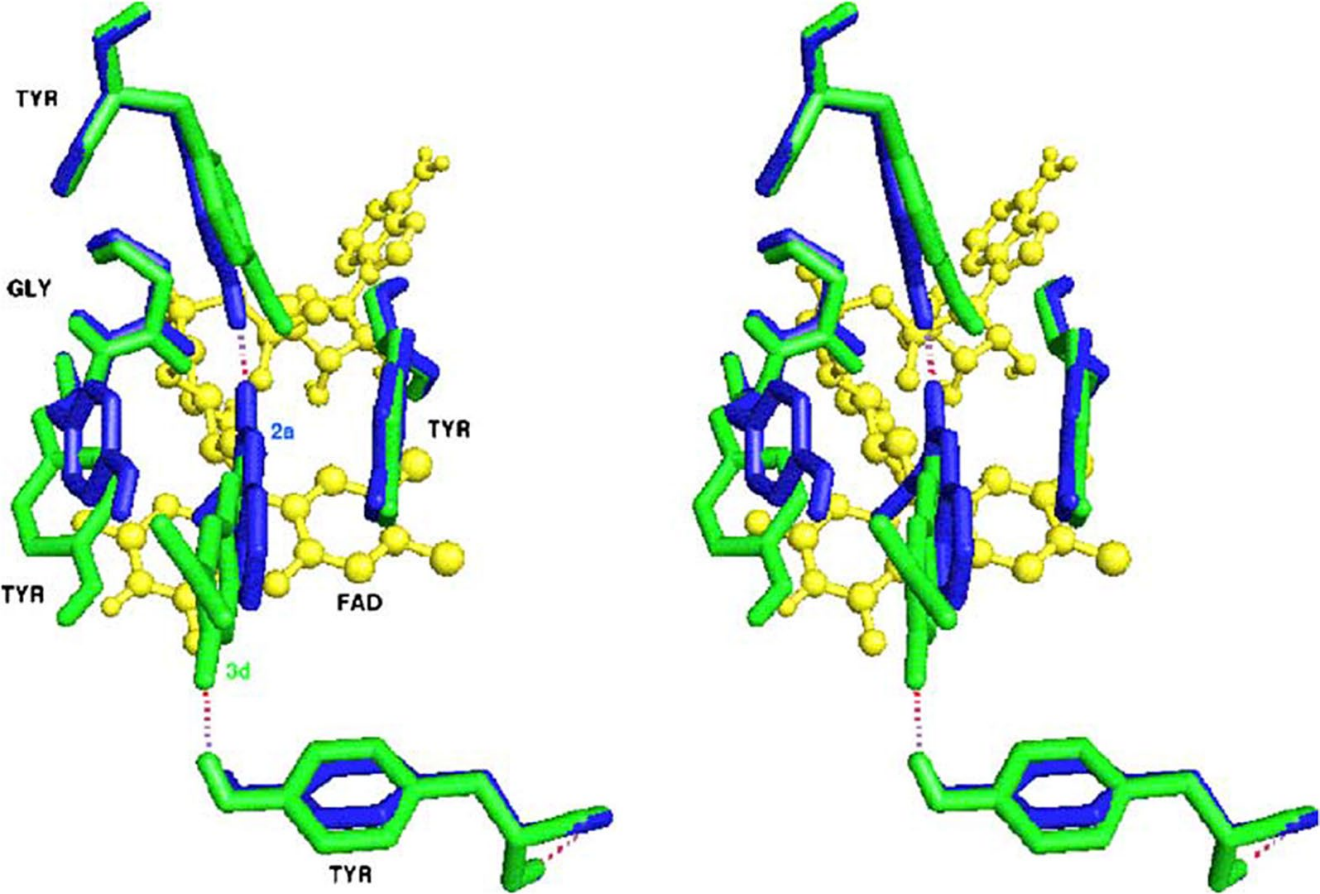
Stereoview comparison of lowest energy MAO-B complexes with **C52** (green polytube model) and **C50** (blue polytube model) within the enzymatic cleft. The cofactor is displayed in ball-and-stick style and the hydrogen bond network in dashed lines [88]

In order to rationalise the activity as well the selectivity and get the insight information related to the enzyme and inhibitor interaction docking was performed with the two most active compounds **C50** and **C52** with the sub isotype MAO-B. It was reported that both these compounds easily fit into the active site of MAO-B with quite different binding modes. The acid moiety of **C50** was reported to direct towards the cofactor and the corresponding acid chloride moiety in compound **C52**, was found to be in the direction opposite protruding towards Tyr326. In addition, both these compounds were found to make one hydrogen bond interaction. For the case of **C50**, the H-bond was established with the phenolic-OH of Tyr188 and for **C52** with the phenolic-OH of Tyr326. On substituting the carboxylic-OH group with chlorine, it was found that compound **C50** was establishing one H-bond with the phenolic-OH of Tyr188, and **C52** was reported to establish another H-bond with the phenolic-OH of Tyr326. The difference in this behaviour was justified by the stronger steric and electrostatic repulsion between the acyl chloride moiety and Gly434. The presence of chlorine at position-6 in the complex, which was actually in close contact with residues Gly434 and tyr188, induces the coumarin moiety of compound **C52** to move far from these two residues resulting in different binding modes for the acid and chloride compounds. Therefore, the main difference between these two binding modes is because of the H-bond acceptor contributions of the hydroxyl side chain moieties of Tyr326 and Tyr188, which can only establish productive interactions with coumarin derivatives (Fig. 56) [88].

**Fig. 57:**
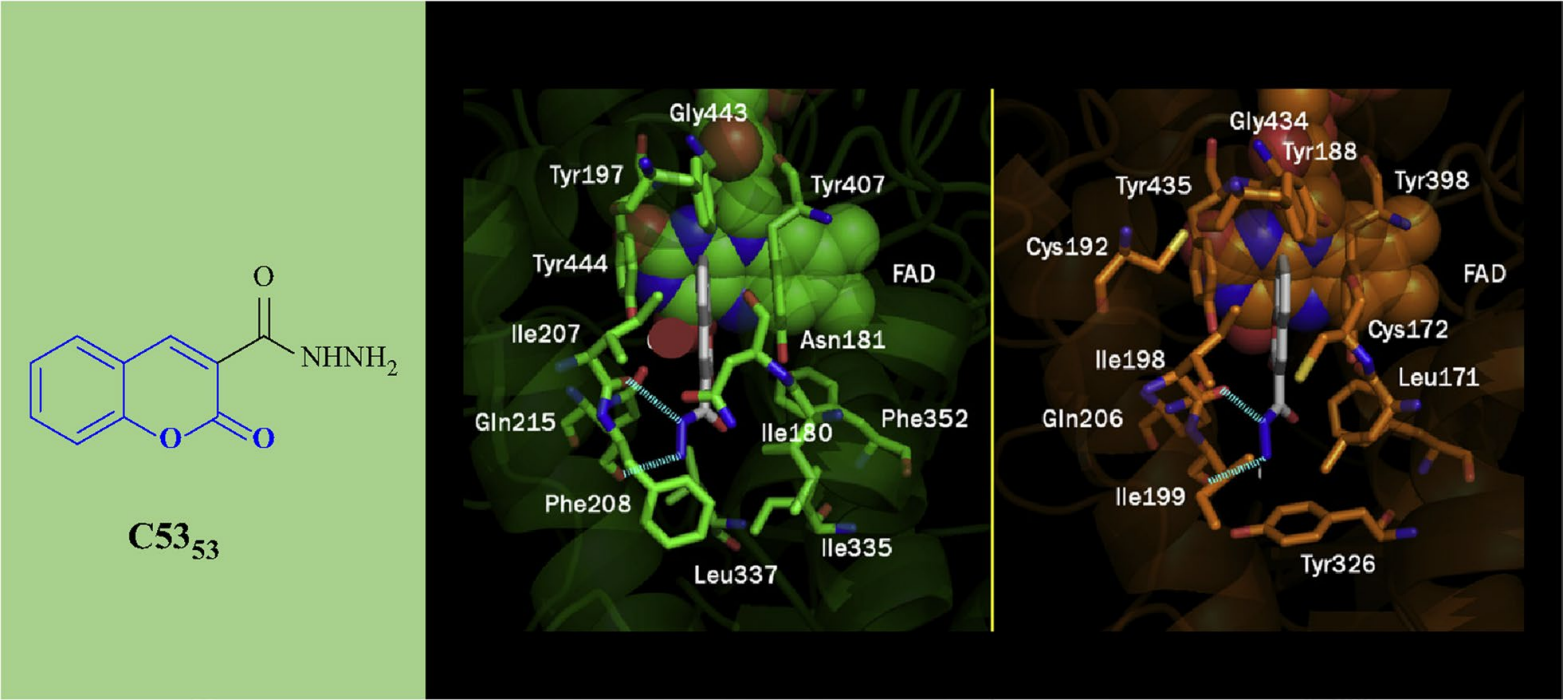
Most stable binding of **C53**_**53**_ with respect to hMAO-A (left side) and hMAO-B (right side). Ligands are shown in CPK colored sticks, interacting residues are coloured in green (hMAO-A) or orange (hMAO-B) carbons in sticks rendering. FAD is reported in spacefill with green (hMAO-A) or orange (hMAO-B) carbon atoms. Non-interacting aminoacids are in transparent green (hMAO-A) or orange (hMAO-B) cartoon notation. Inhibitor-enzyme hydrogen bonds are reported in cyan dashed lines [89]

Secci et al. [89] designed and synthesized a big library of 3-carbonyl (**C53**_**1**_–**C53**_**26**_), 3-acyl (**C53**_**27**_–**C53**_**52**_), and 3-carboxyhydrazido (**C53**_**53**_–**C53**_**58**_) coumarins derivatives and tested them in vitro for their human monoamine oxidase A and B (hMAO-A and hMAO-B) inhibitory activity. They performed a detailed study related to the position of different substituents on the coumarin ring, that in which way or the other they are effecting the MAO inhibitory activity and selectivity. They concluded from their observation that introduction of hydrazido moiety at position-3 (**C53**_**53**_) and substitution at position-7 of 3-ethylester coumarin ring was enhancing the IC_50_ value from μM to the nM range (IC_50_ = 3.22 nM) with respect to the reference drug R-(–)-deprenyl and also displaying selectively for the hMAO-B isoform. Hence to get the insight information for the most potent compound of the library viz **C53**_**53**_, molecular docking was performed against MAO-A and MAO-B taking in consideration the most stable binding configuration of **C53**_**53**_ (Fig. 57). Coumarin moiety was reported to be into the binding cleft of hMAOs and was found to form 2 H-bonds with the targets and π–π stacking interactions with Tyr407 and Tyr444 of hMAO-A and with Tyr435 and Tyr398 of hMAO-B. Table 2 displays that the ΔG of binding was in good correlation (r^2^ = 0.76) to the inhibitory activities for compound **C53**_**53**_ [89].

**Table 2:** Comparison between theoretical affinity and experimental inhibition data of **C53**_**53**_

Compound	hMAO-A		hMAO-B	
	pIC_50_	ΔG (kcal/mol)	pIC_50_	ΔG (kcal/mol)
**C53**_**53**_	4.00	− 35.10	8.49	− 38.10

**Fig. 58:**
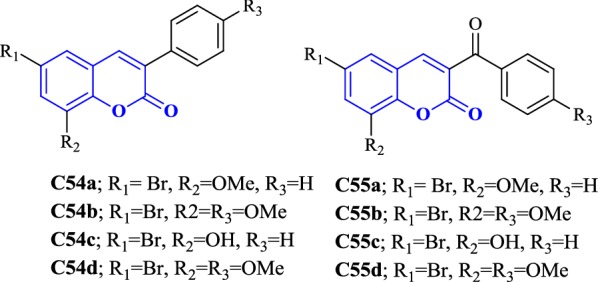
Molecular structure of compounds **C54a**–**d** and **C55a**–**d**

Matos et al. [90] designed and synthesized two different series of 3-phenylcoumarins (**C54a**–**C54d**) and 3-benzoylcoumarins (**C55a**–**C55d**) (Fig. 58). Bromo atom was introduced at position-6 of the coumarin ring whereas OCH_3_/OH group was introduced at position-8 and then these compounds were evaluated for their MAO-A and MAO-B inhibitory activities using R-(–)-deprenyl and iproniazide as standards. Interestingly, the presence and absence of the carbonyl moiety between the coumarin ring and 3-phenyl ring was focused for the MAO-A/MAO-B inhibitory properties. The 3-phenylcoumarin derivatives, **C54a** (IC_50_ = 83.48 nM) with bromo at position 6 and methoxy at position 8 and derivative **C54b** (IC_50_ = 1.35 nM) with bromo at position 6, methoxy at position 8 and another methoxy at the para position of the 3-phenyl ring were found to be the most active compounds among the eight. To be added, it was reported that the introduction of a substituent in the para position of the 3-phenyl ring might had helped in improved activity, specifically when it is a methoxy group (**C54b**). This derivative was found displaying higher MAO-B activity even better than the standard R-(–)-deprenyl (IC_50_ = 19.60 nM) with the inhibition level more than 74,074-fold with respect to its MAO-A isoform and as compared to other derivatives. On the other hand, the presence of hydroxyl group in the places of methoxy group was however found lowering the activity and selectivity as reported for the derivatives **C54c** (IC_50_ = 30.91 nM) and **C54d** (IC_50_ = 16.87 nM). On the comparison of these results with the other series of 3-benzoylcoumarins where the 3-phenyl group was replaced by the 3-benzoyl group, it was reported that the carbonyl moiety was selectively lowering the MAO-B activity as compared to MAO-A activity and the structure activity relationship was just the opposite as found for the 3-phenylcoumarins. In here, the 6-bromo-8-hydroxy derivatives (**C55c** and **C55d**) (IC_50_ = 46.81 nM and 19.17 nM) were reported to be more MAO-A active than their corresponding 6-bromo-8-methoxy forms (compounds **C55a** and **C55b**), which do not displayed any of the MAO inhibitory properties [90].

**Fig. 59:**
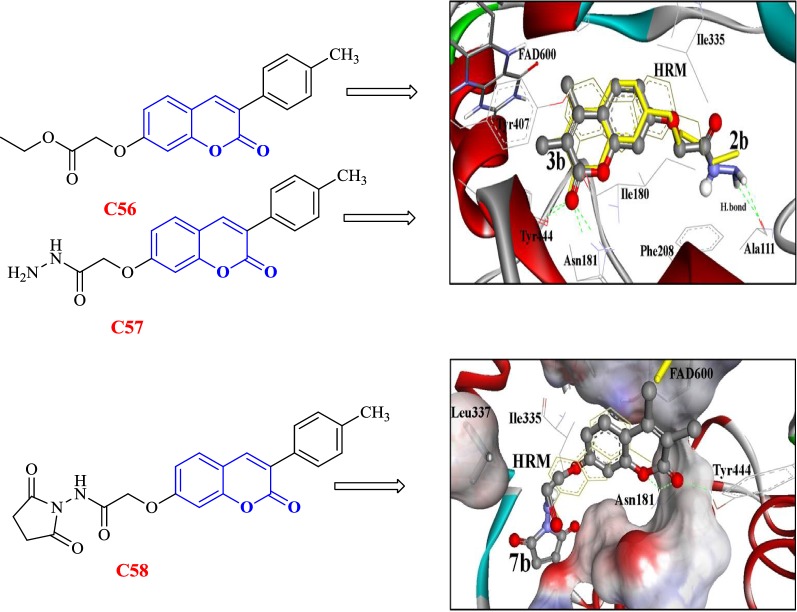
Molecular structures and binding affinities of compound **C56** (yellow stick) and **C57** (ball and stick, coloured by element) and **C58** into binding pocket of MAO-A (2Z5X) [91]

Abdelhafez et al. [83] designed and synthesized 7-oxycoumarin derivatives and evaluated them in vivo and in vitro for their MAO-A and MAO-B inhibitory properties. The in vitro studies provided very good results with Ki values in the range of picomolar in the terms of potency and MAO-A selectivity, whereas in vivo studies provided with better direct correlation. Overall, compounds **C56**, **C57** and **C58** were reported to be the most potent in vitro and in vivo MAO-A and MAO-B inhibitors. In order to get the insight of the binding nature of the compounds, molecular docking was performed by docking these derivatives into the binding sites of MAO-A (PDB: 2Z5X) and MAO-B (PDB: 2XFN). The aforementioned docking results of the biologically potent derivatives **C56**, **C57** and **C58**, displayed the best fitting into the binding site of MAO-A as compared to the other derivatives. Compound **C56** was found to exhibit two H-bonds between its 2-C=O and OH of Tyr444 and NH of Asn181 amino acid within RMSD of 0.66 Å. Compound **C57** was reported to tightly bound with four H-bonds through the 2-C=O terminal and NH_2_ group and OH of Tyr444, NH of Asn181 and C=O of Ala111 within RMSD of 1.51 Å. Analogue **C58** was reported to display very good binding affinity and was found to tightly bound with three H-bonds between the O (pyrone) and 2-C=O, with NH of Asn181 and OH of Tyr444, within RMSD of 1.01 Å (Fig. 59) [91].

**Fig. 60:**
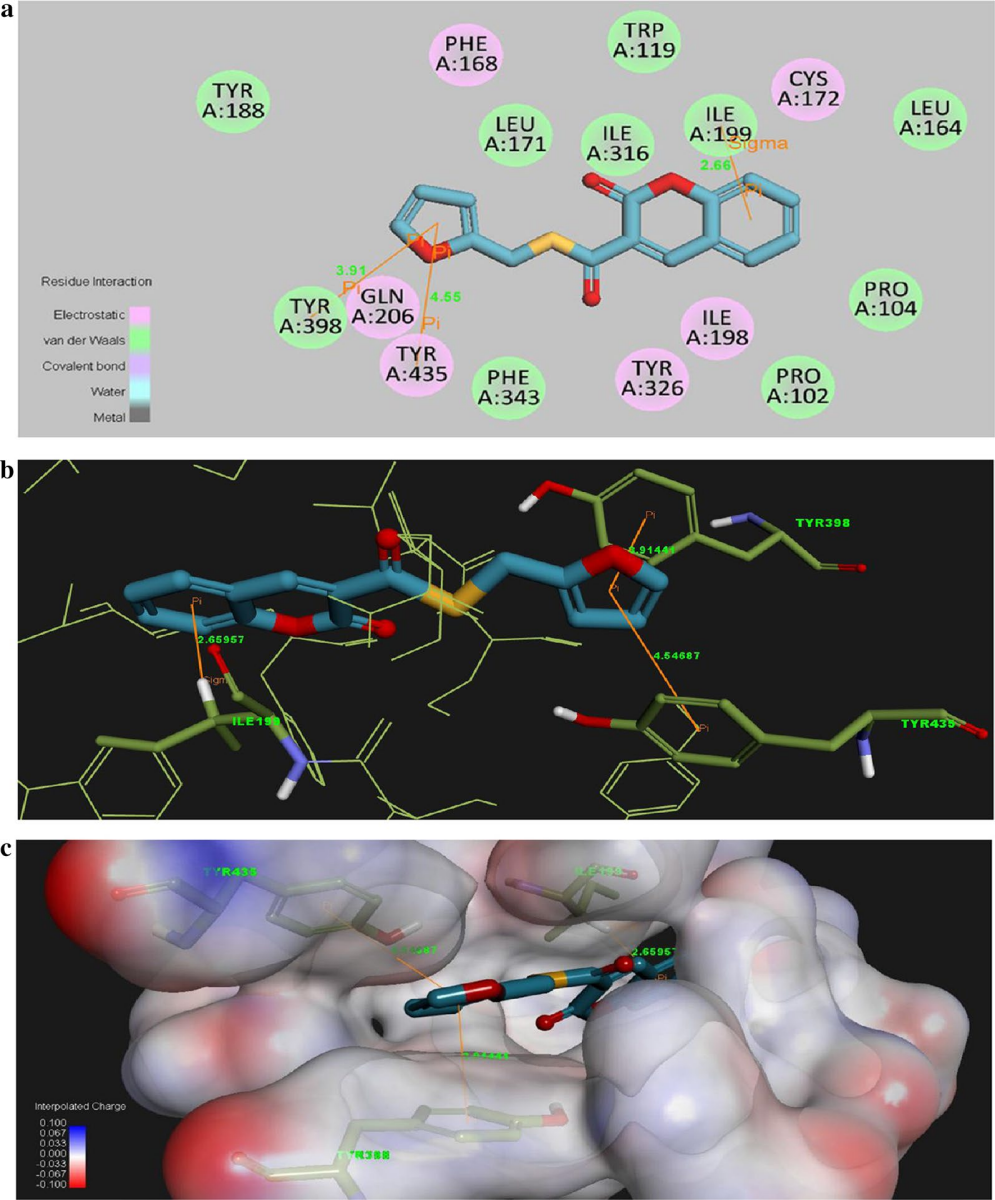
Molecule docking of the compound **C59** into the ligand binding model site of MAO-B (**a**). 2D Picture of binding (**b**). 3D Picture of binding (**c**). Molecule well fitted in the active pocket at the enzyme surface [92]

He et al. [92] designed and synthesized two new series of coumarin derivatives viz 2H-chromene-3-carboxamides and S-2H-chromene-3-carbothioates as hMAO inhibitors and performed their 3D docking studies to get the better assess to their selectivity. Among all the derivatives, analogue **C59** was reported to be the most active (IC_50_ = 0.21 μM) and 189.2 folds more MAO-B selective with respect to the MAO-isoform and as compared to the standard iproniazid, with IC_50_ of 7.65 μM and selectivity of 1-fold. Its docking study revealed that the H-atom of the amino acid residue Ile199 was responsible for the binding of ligand’s benzene ring through the sigma-π interaction. Additionally two π–π interactions were also reported between the compound and the amino acid around it. One was between the furan-ring of compound **C59** and benzene-ring of Tyr398 and the other between the furan-ring of compound **C59** and benzene-ring of Tyr435 (Fig. 60) [92].

**Fig. 61:**
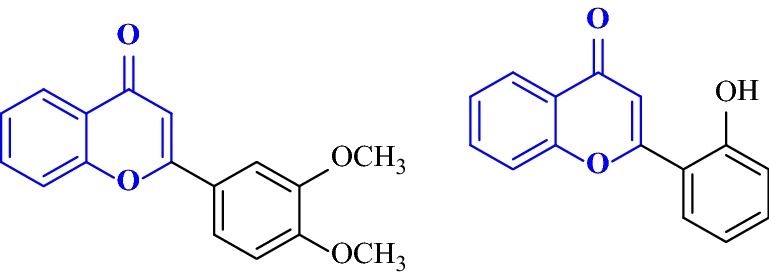
Molecule structures of compounds **C60** and **C61**

**Fig. 62:**
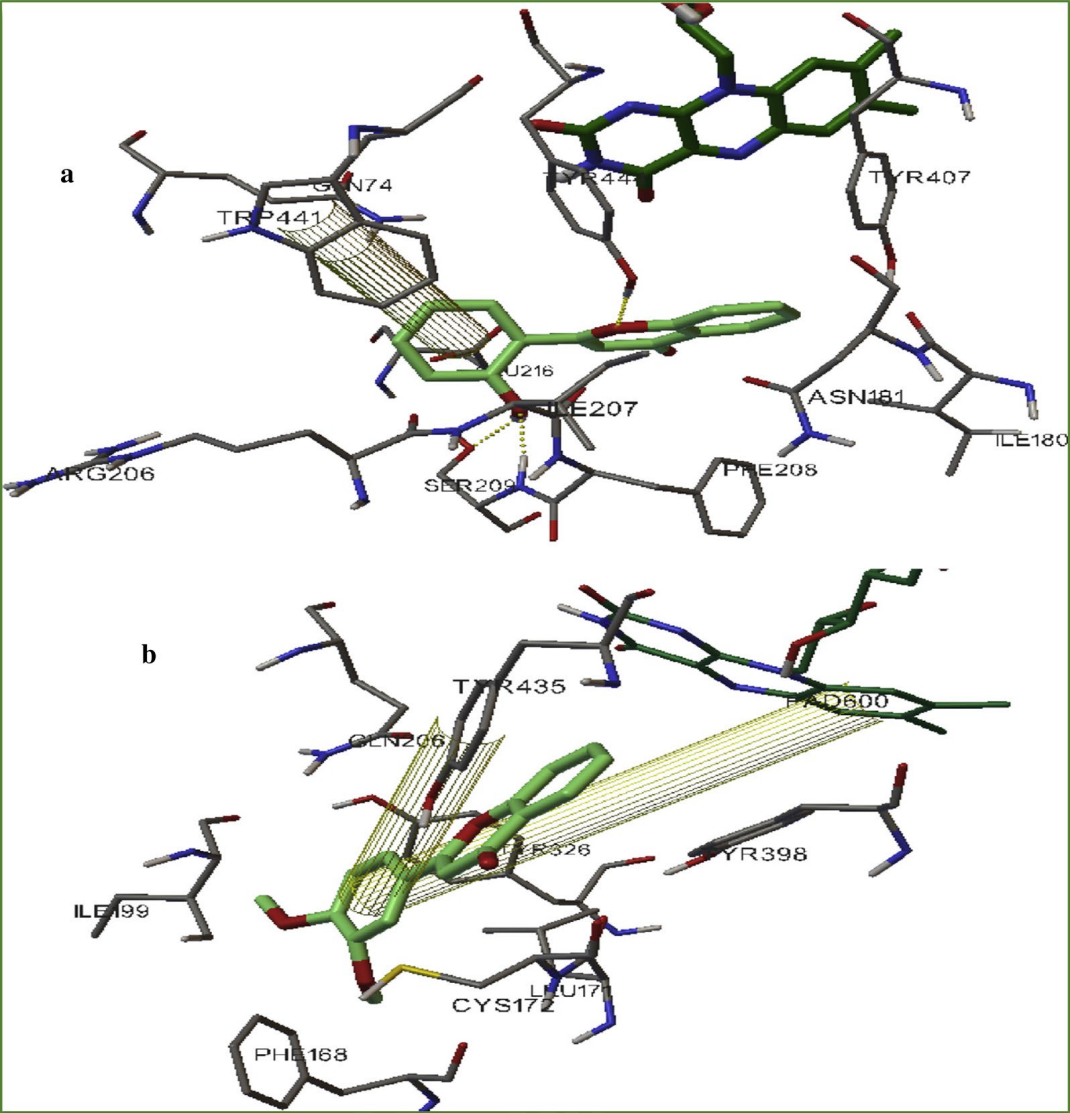
**a** Interaction of compound **C61** with human MAO-A. **b** Interaction of compound **C60** with human MAO-B [93]

Nayak et al. [93] designed a large library of twenty 2-aryl-4H-chromen-4-ones coumarin derivatives and performed their hMAO inhibitory activity using recombinant hMAO-A and hMAO-B against selegiline as standard. Result showed that sixteen compounds were selective towards hMAO-B, and among them compound **C60** which was carrying 3,4-di-OMeC_6_H_3_ group was found to be the most active (IC_50_ = 0.16 μM) and also the most selective (SI = 30.0) MAO-B inhibitor. With respect to this, compound **C61** carrying the 2-OH-C_6_H_4_ group was found to be the most active (IC_50_ = 0.52 μM) and the most selective (SI = 11.5) MAO-A inhibitor (Fig. 61). The reason behind the potent hMAO-B inhibition property and selectivity of the sixteen derivatives was due to the presence of the deactivating functional groups at para position of the phenyl ring as compared to the compounds with unsubstituted phenyl ring. The more the deactivating nature of the substituent present on the phenyl ring, the more was the recorded potency (NO_2_ > Br > Cl > CN) due to less difference in interaction with the MAO-B protein. However, the selectivity criteria for MAO-B was recorded to follow the reverse order. Additionally the compounds possessing meta positioned activating substituents were also reported to exhibit good MAO-B but for sure not better than the derivatives possessing para positioned deactivating functional groups. Molecular docking performed on the most active compound **C60** displayed that the phenyl ring bearing 2 OCH_3_ groups, attached to the position-2 of the chromenone was accommodated within the hydrophobic tunnel leading to solvent exposed entrance whereas the ring of the chromenone was accommodated in the cage composed by FAD, Tyr398 and Tyr435. A π–π interaction between the phenyl ring oriented at position-2 of chromenone and Tyr435 and FAD was also observed. Docking study of the most active MAO-A inhibitor **C61** revealed three H-bonding interaction viz., (i) hydroxyl hydrogen of **C61** with side-chain hydroxyl oxygen of Ser209, (ii) hydroxyl oxygen of **C61** with backbone amino hydrogen of Ser209 and (iii) chromenone ring oxygen with hydroxyl hydrogen of Tyr444. A π–π interaction between o-hydroxy phenyl group at 2nd position of chromenone and Trp441 was also obswerved. All these interactions kept pocket1 (Aromatic cage: FAD, Tyr407, Tyr444) unoccupied, pocket 3 (Ile180, Ile335, Leu337, Met350, Phe352) partially occupied by chromenone benzene ring and pocket 2 (Gly71, Gln74, Arg206, Ile207, Phe208, Glu216, Trp441) partially occupied by o-hydroxy phenyl ring at 2nd position of chromenone (Fig. 62) [93].

### Coumarin analogues as selective MAO-B inhibitors

**Fig. 63:**
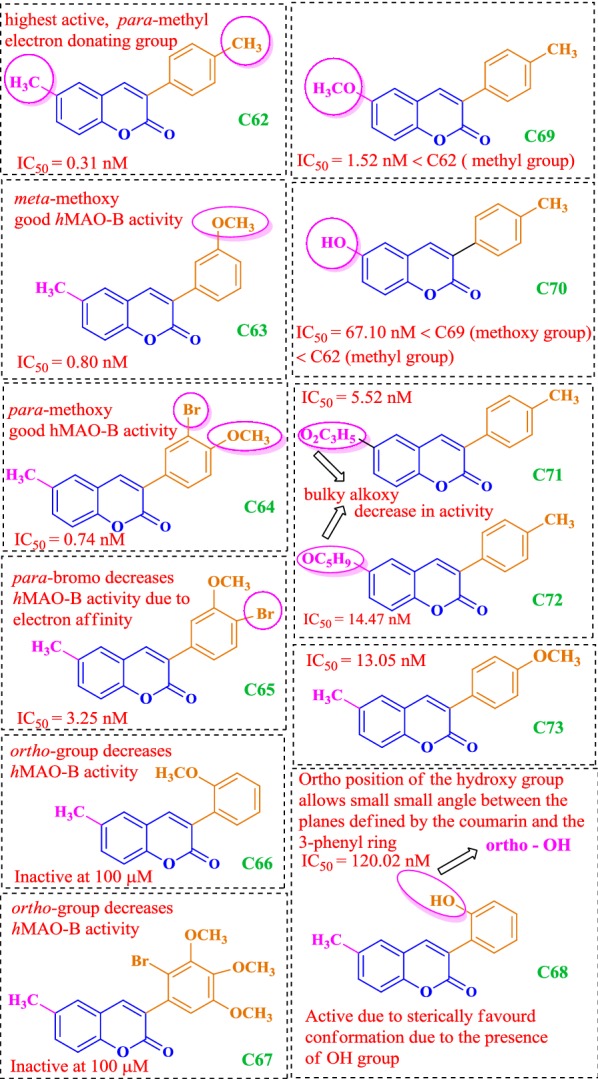
Molecular structures of compounds **C62**–**C73**

Matos et al. [94]  synthesized 22 derivatives of 3-arylcoumarins with various alkyl, hydroxyl, halogen, and alkoxy groups in the two benzene rings and check their potential against hMAO-A and hMAO-B. The results showed these compounds to be selective against isoform hMAO-B isoenzyme, displaying very good _IC50_ values in the range of nanomolar and picomolar. Compound **C62**, was the most potent compound of the series, even 64 times stronger than the drug selegiline and displaying highest IC_50_ of 0.31 nM against hMAO-B, followed by compound **C63** and **C64**, displaying IC_50_ against hMAO-B of 0.80 nM, and 0.74 nM, respectively. It was found that modification with the electron donating group at para position of the benzene ring helped in increasing the selectivity towards the isoform B as reported for derivative **C62** possessing a p-methyl moiety in the 3-phenyl ring and a 6-methyl moiety in the aromatic coumarin ring as compared to the compound **C65**, with a para-bromo and meta-methoxy groups, respectively. Presence of electron withdrawing group at para in its case was reported to diminish the activity. Compound **C63** possessed a meta-methoxy and compound **C64** possessed a para-methoxy and a meta-bromo group, respectively in the 3-phenyl ring and a 6-methyl moiety in the aromatic coumarin ring. Additionally, it was found that almost any substituent at ortho position in the 3-phenyl ring tends to decrease the activity as observed for the cases **C66** and **C67** with an ortho-methoxy and an ortho-bromo except for the compound **C68** with an ortho-hydroxy group, which was further explained by docking studies. Moreover, the presence of 6-methyl group in the coumarin moiety tend to increase the activity (**C62**) as compared to the presence of methoxy (**C69**) or hydroxyl groups (**C70**). Also, bulkier alkoxy groups at 6-position such as, 2-oxopropoxy (**C71**) or a cyclopentyloxy (**C72**), significantly reduced the activity (Fig. 63) [94].

**Fig. 64:**
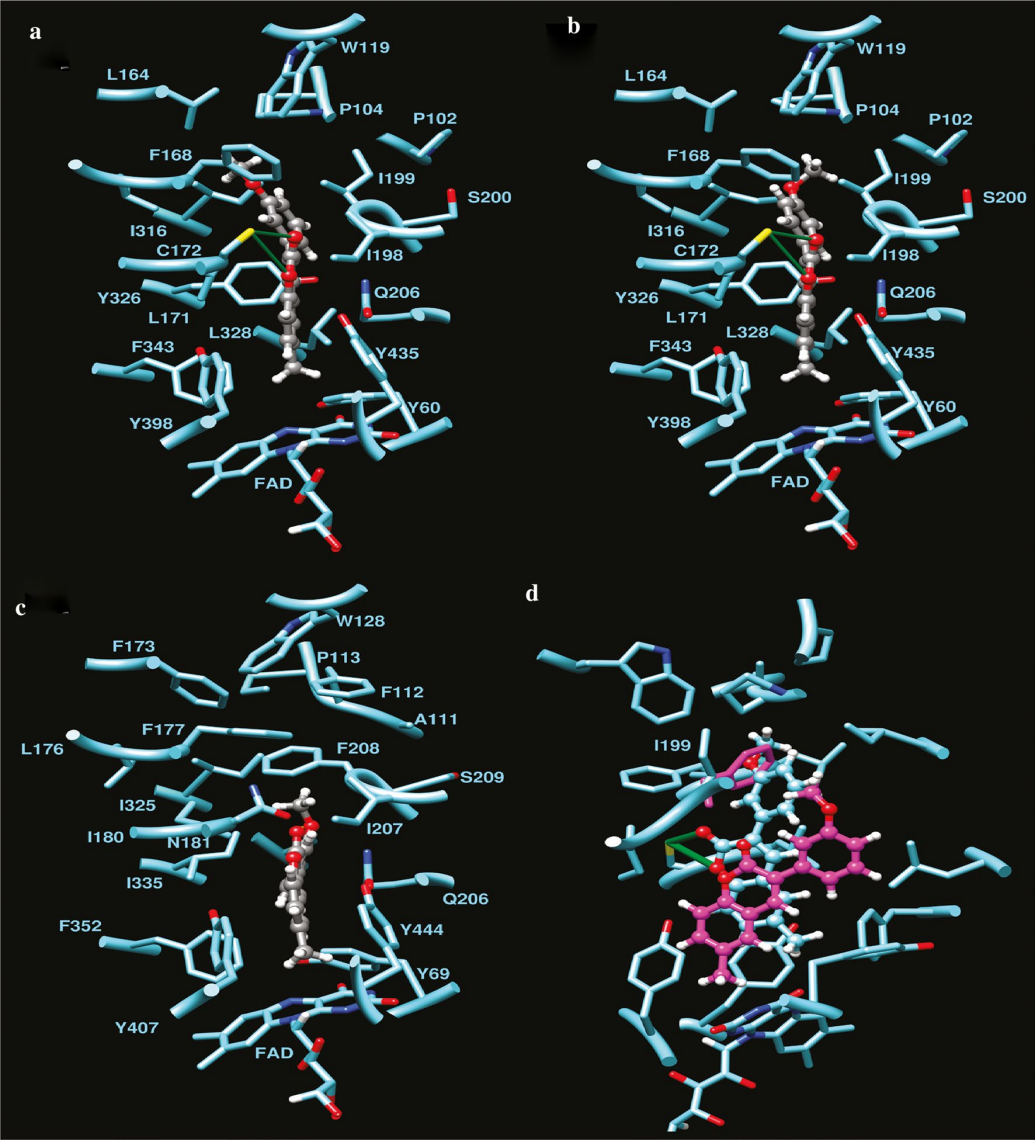
Molecular docking studies of coumarin derivatives **C73** and **C63**. Most stable binding poses of compound **C63** (**a**) and **C73** (**b**) into MAO-B binding site, and compound **C63** (**c**) into MAO-A binding site. Compounds are colored by atom type, and predicted H-bonds are represented as green pseudobonds. Superposition of binding poses of compound **C63** (**d**) to both isoenzymes, MAO-A (ligand and Phe208 in magenta), and MAO-B [94]

All these results were found to be consistent with the docking results with the observation that small substituents are more favourable at sixth position of the coumarin nucleus. The bulky substituents resulted in the disruption of the proposed binding mode by occupying most of the space and hence resulting in the loss of the key interactions necessary for the stabilization of the ligand-enzyme complex (**C71** and **C72**). Same ways a small angle between the planes of coumarin and 3-phenyl ring was observed in the docking stimulations, when OH group was present at ortho position of the 3-phenyl ring (**C68**). However these conformations were sterically prohibited when methoxy/bromo group was present at the same position and abolished the activity against hMAO-B (**C66** and **C67**). Results showed that para or meta substituents in the 3-phenyl ring allowed the compound to occupy the hydrophobic subpocket making it considerably active (**C63** and **C73**) (Fig. 64a, b). Moreover, it was also clear by comparing the predicted binding mode of compound **C63** to MAO-A (Fig. 64c) and MAO-B (Fig. 64d) that, the entrance cavity only exists in MAO-B and its formation was not affected by the presence of Ile199 residue whereas, in MAO-A its formation was prevented by the presence of the Phe208 residue in the same position as MAO-B (Fig. 64) [94].

Matos et al. [85] also synthesized another series of 6-methyl-3-phenylcoumarins **C74**–**C77** as potent MAO-A and MAO-B inhibitors. Results showed the compounds to be selective against isoenzyme MAO-B displaying very good IC_50_ values in lower nanomolar range. Derivatives **C74**, **C75** and **C77** showed very good inhibition and in particular compound **C77**, with the methoxy substituent at meta position was found to be 24 times more potent and selective than the reference compound R-(–)-deprenyl (IC_50_ = 19.60 nM) displaying IC_50_ value of 0.80 nM. On the other hand, compound **C76** with ortho methoxy group does not displayed any inhibitory activity even at the highest tested concentration level making it clear that the ortho position is not favorable for enzymatic inhibition (Fig.. 65) [85].

**Fig. 65:**
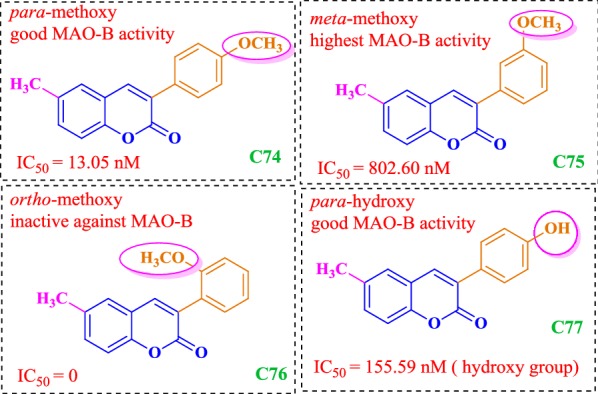
Molecular structures of the compounds **C74**–**C77**

**Fig. 66:**
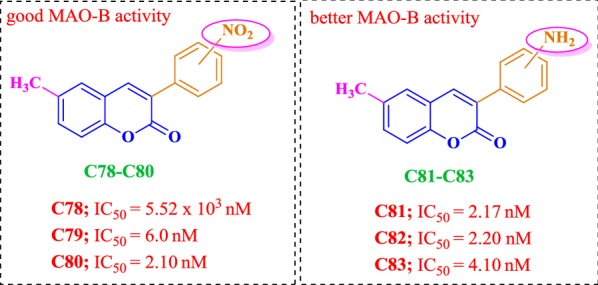
Molecular structures of the compounds **C78**–**C83**

In the same context Matos et al. [96] designed two series of amino (**C78**–**C80**) and nitro 3-arylcoumarins (**C81**–C**83**) and compared them as hMAO-A and hMAO-B inhibitors [95]. The overall result showed the compounds to be selective against isoenzyme hMAO-B. Comparative study revealed that amino compounds were more potent and selective against MAO-B than the nitro compounds, though the nitro compounds were also found to display quite good MAO-B inhibitory properties. The comparative IC_50_ values are displayed in Fig. 66 against the standard selegiline with IC_50_ value 19.60 nM.

**Fig. 67:**
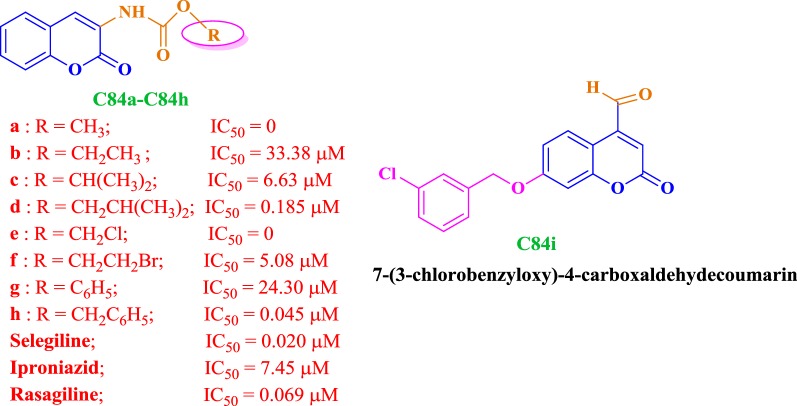
Molecular structures of the compounds **C84a**–**C84i**

In continuation to their work, Matos et al. [95] synthesized a series of (coumarin-3-yl)carbamates **C84a**–**C84h**, and subjected them against MAO inhibitory activity (Fig. 67). Interestingly, the compounds were reported to be selective MAO-B inhibitors and their selectivity depend on their substituents linked to the carbamate group, whereas none of the synthesized derivatives exhibited activity against MAO-A isoform even at the highest concentration level of 100 μM [95]. Among all the compounds, derivative **C84h** (benzyl(coumarin-3-yl)carbamate), was found to be the most active, which displayed IC_50_ value of 0.045 μM, comparable with selegiline, the reference compound with IC_50_ of 0.020 μM. The IC_50_ values of all the compounds with their substituents are displayed in Fig. 67.

**Fig. 68:**
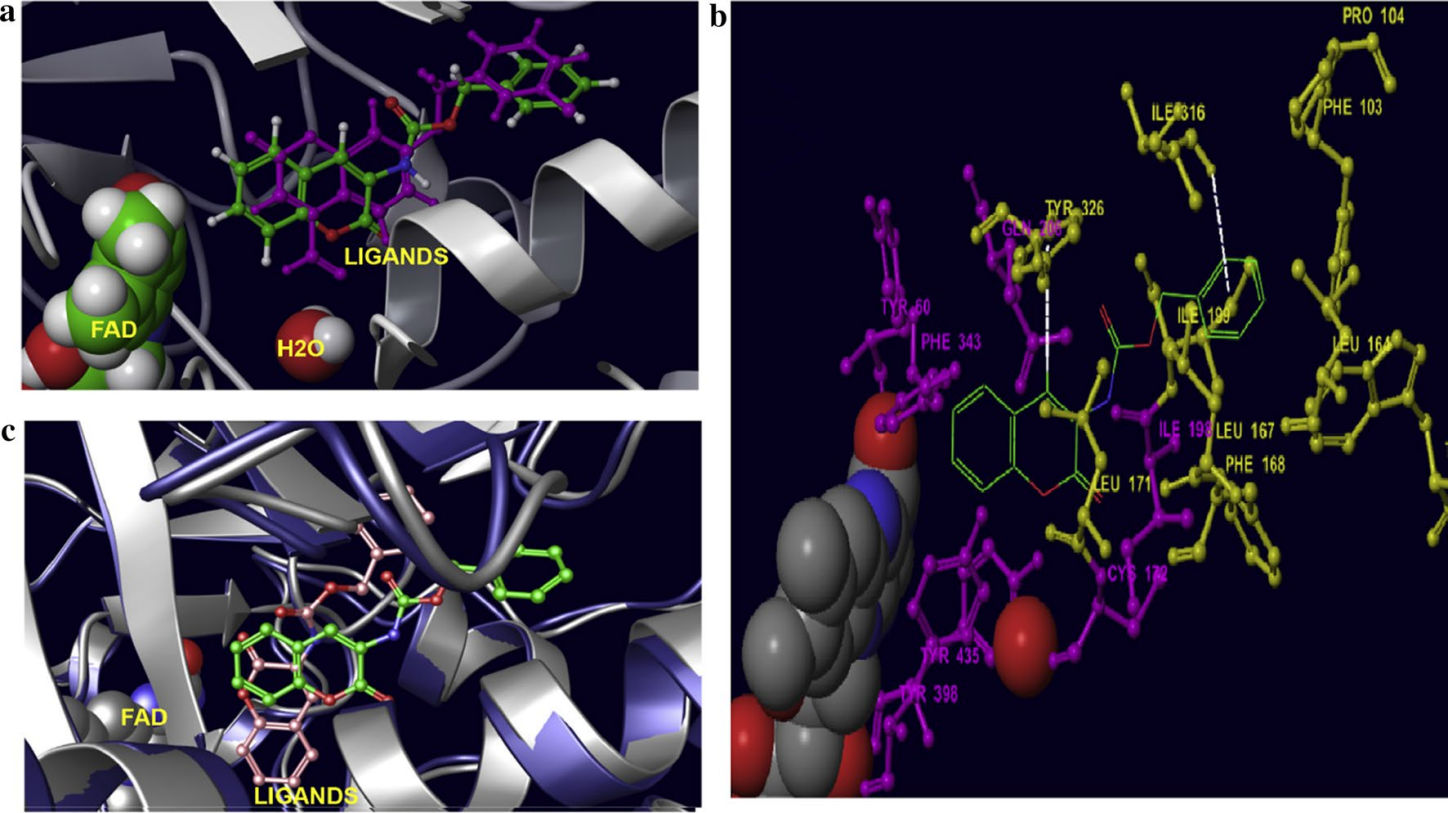
Comparison of the co-crystallized ligand **C84i** (purple color) and the calculated pose for the compound **C84h** (colored by element, green carbons) by the docking simulation in the hMAO-B (**a**, **b**, **c**) [95]

Further molecular docking study was performed to get the insight of the enzyme-inhibitor key interaction, which contributes, to the most stable complex conformation. It was found that the length of the compound **C84h** causes the coumarin ring’s position to shift towards the FAD, compared to the co-crystallized coumarin **C84i** (7-(3-chlorobenzyloxy)-4-carboxaldehydecoumarin) in 2V60 and the benzyl group was found positioned towards the hydrophobic pocket in the entrance cavity establishing hydrophobic interactions with residues Phe103, Pro104, Trp119, Leu164, Leu167, Phe168, Ile199 and Ile316 (Fig. 68a). Different residues were found to interact with the ligand through van der Waals interactions (Fig. 68b), mainly defined by Ile199, Leu171, Gln206, Tyr326, Ile198, Tyr398 and Phe168. In addition to these, docking results without water molecules showed a conformation for compound **C84h** placed deeply in the cavity (Fig. 68c).

**Fig. 69:**
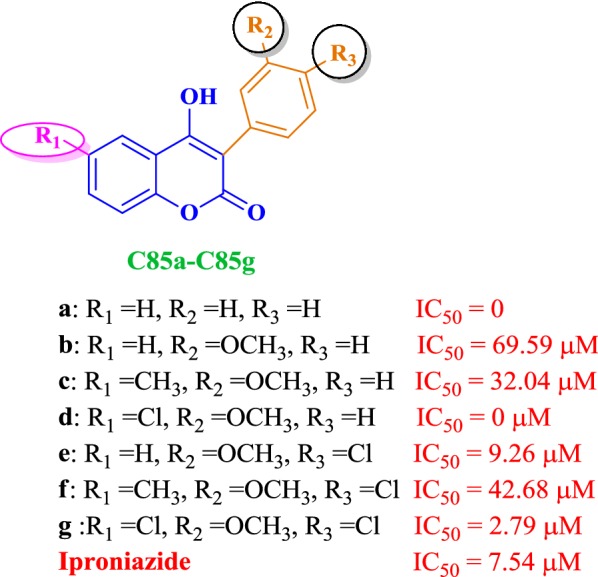
Molecular structures of the compounds **C85a**–**C84g**

**Fig. 70:**
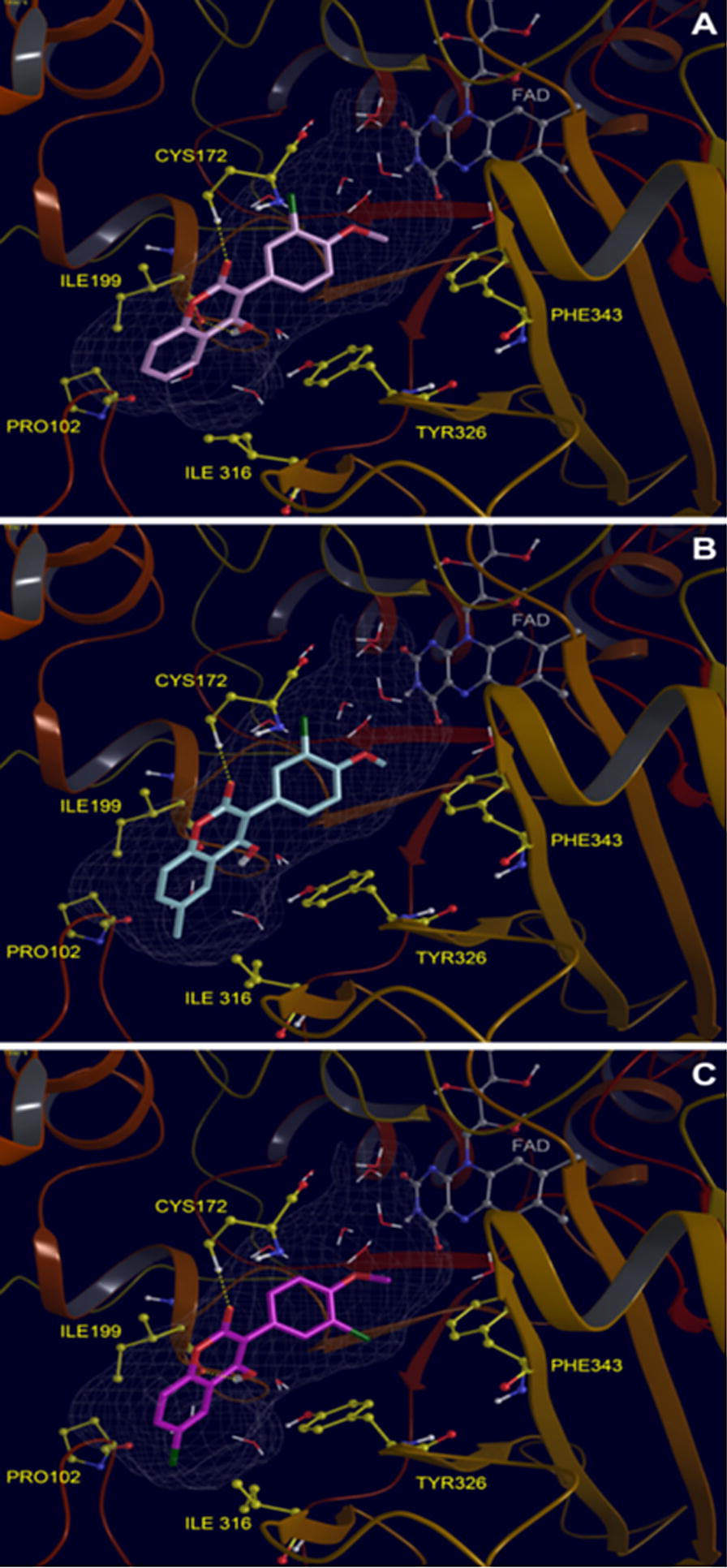
Best docking poses retrieved for compounds **C85e** (**A**), **C85f** (**B**) and **C85g** (**C**) into the MAO-B (PDB code: 2V60). Coumarins are represented in tube with carbon atoms colored in plum for **C85e**, turquoise for **C85f** and purple for **C85g** [97]

Serra et al. [97] synthesized a series of 3-aryl-4-hydroxycoumarin derivatives (**C85a**–**C85g**) as selective MAO-B inhibitors by introducing various groups in the 3-phenyl ring attached to the coumarin core and none of the compounds were found to show MAO-A inhibitory activity even at the highest concentration level of 100 μM (Fig. 69) [97]. The MAO inhibitory activity was evaluated in vitro by the measurement of the enzymatic activity of human recombinant MAO isoforms expressed in BTI insect cells infected with baculovirus. It was reported that para methoxy (**C85e**, IC_50_ = 9.26 μM) and meta chloro groups (**C85f**, IC_50_ = 42.68 μM) in the 3-phenyl ring influenced the inhibitory activity up to interesting levels. Additionally, it was also reported that the presence of a chloro atom in the six position of coumarin ring in case of compound **C85g (**IC_50_ = 2.79 μM**)**, improved the inhibitory activity and selectivity as compared to the standard iproniazide (IC_50_ = 7.54 μM). Molecular docking study was performed in order to get the insight and to understand the energetically favoured orientations inside the binding pocket of isoenzyme MAO-B, of the top three active compounds viz **C85e**, **C85f** and **C85g**. Very interestingly, same binding pattern was observed for all the three derivatives which is depicted in Fig. 70. All the three hydroxycoumarin moieties were reported to occupy the entrance of the cavity and the 3-arylcoumarin rings were reported to direct towards the FAD cofactor. Cys172 was involved in H-bond formation between carbonyl oxygen of all three coumarins, hence playing its role in complex stabilization. A π–π stacking interaction between Tyr326 and 3-arylcoumarin rings was also observed. Van der Waals and electrostatic interactions with Pro102, Ile316 and Phe343 were also observed for all the three hydroxycoumarin. The para-OCH_3_ group was reported to occupy similar position in all the three coumarins while the Cl substituent in the 3-phenyl ring was found to opt an opposite orientation, in case of compounds **C85e** and **C85g**, hence appearing to be unimportant for the modulation of MAO-B inhibitor activity.

**Fig. 71:**
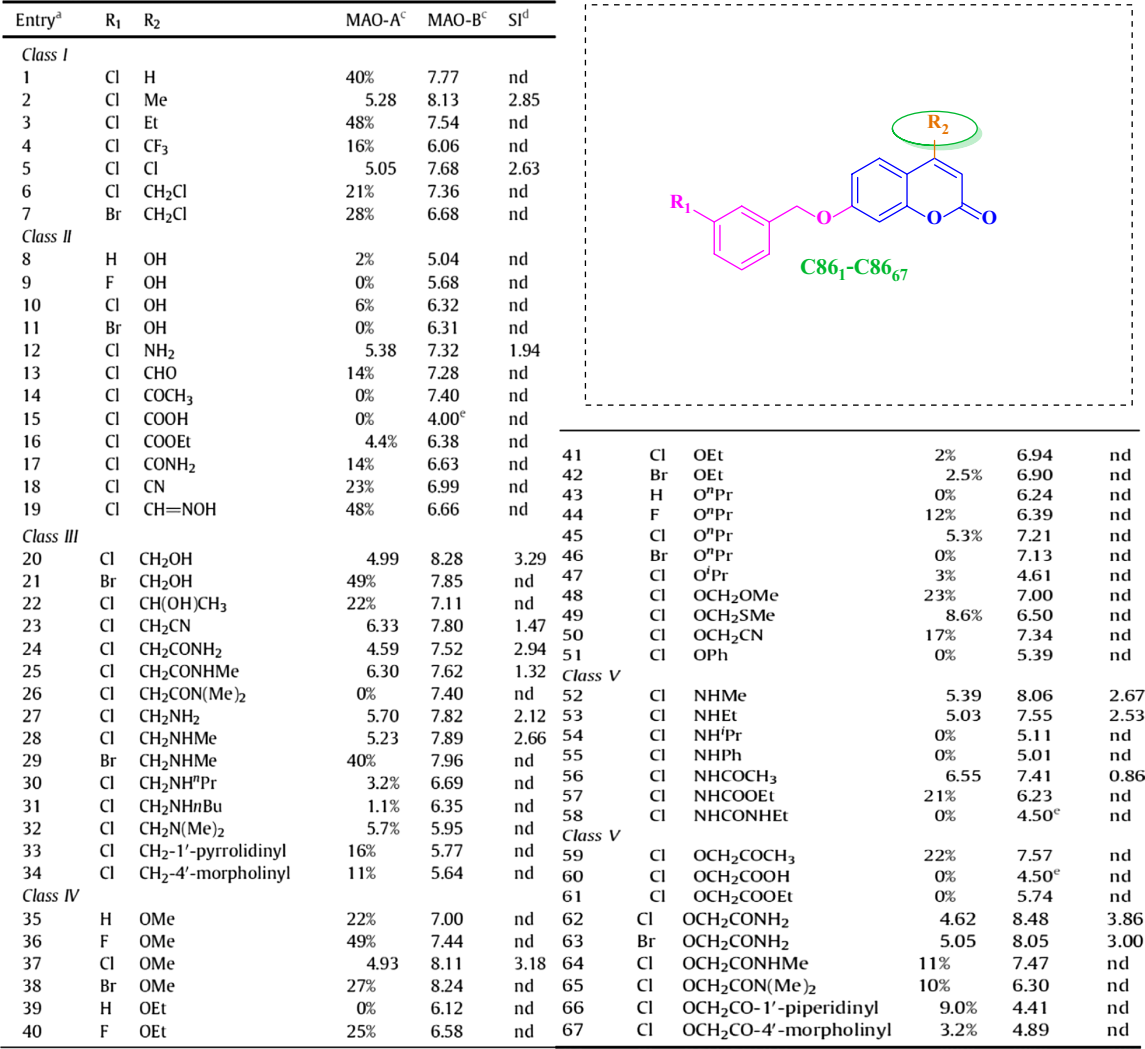
Molecular structures of the compounds **C86**_**1**_–**C86**_**67**_

In order to search potent and selective MAO-B inhibitors Pisani et al. synthesized a large library of 67 derivatives of 2H-chromen-2-one with fine molecular tuning at position 4 of the coumarin skeleton (Fig. 71) [98]. To their interest, all the 67 compounds exhibited high MAO-B selectivity, and few were found displaying quite better potency in the low nanomolar range. For example derivative, 7-metachlorobenzyloxy-4-oxyacetamido-2H-chromen-2-one (**C86**_**62**_) displayed highest MAO-B potency with IC_50_ value of 8.48 μM and selectivity ratio of 7244, over the sub isoform MAO-A.

**Fig. 72:**
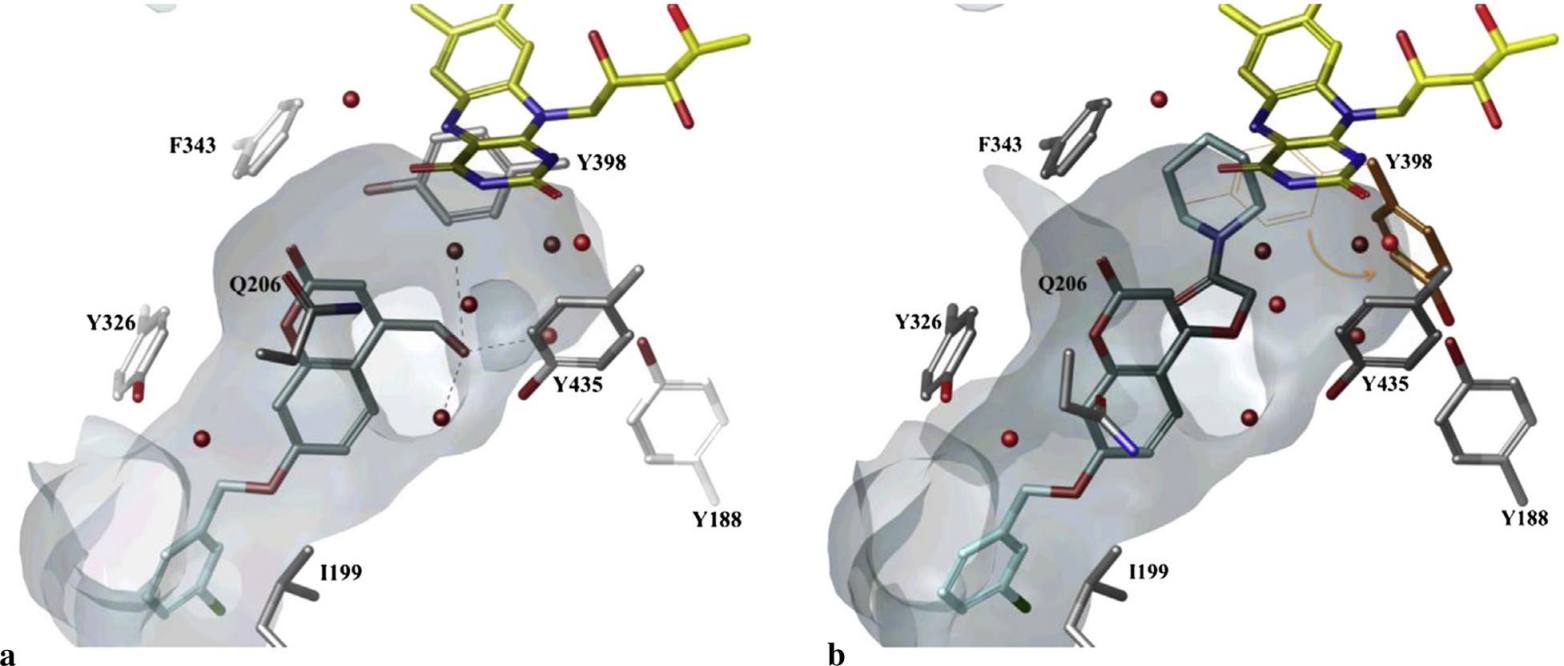
Top-ranked docking pose of compounds **C86**_**20**_ (**a**) and **C86**_**66**_ (**b**) [98]

Molecular docking was performed to obtain the reason behind the drop of affinity and lowering the DS due to the presence of bulkier, branched or cyclic groups which cause steric crowding at position-4 of the coumarin nucleus and for this aspect, compounds **C86**_**6**_, **C86**_**20**_, **C86**_**33**_, **C86**_**34**_, **C86**_**51**_, **C86**_**54**_, **C86**_**62**_ and **C86**_**66**_ were selected as model compounds to observe their binding interactions. To their satisfaction, they reported the experimental results in agreement with the docking results. It was found that one of the most active compound **C86**_**20**_ (IC_50_ = 8.28 μM) with 4-hydroxymethyl group was accommodated perfectly within the binding site of MOA-B, as depicted in Fig. 72a, and formed stabilized HB network, by involving three structural water molecules and for this case the DS was calculated be 79.85 kJ/mol. When a chlorine atom replaced the 4-hydroxymethyl group (**C86**_**6**_) then DS was calculated to be 76.61 kJ/mol and also a slight decrease in the activity was reported (IC_50_ = 7.36 μM), which was justified on the basis of loss of HB interactions. For the case of the most active compound **C86**_**62**_ (IC_50_ = 8.48 μM), possessing a large polar group at position-4 of the coumarin nucleus, displayed HB interactions with specific residues of amino acids and thereby increasing the the DS (74.76 kJ/mol). Compound **C86**_**66**_ with a bulky substituent at position-4 was found to rearrange in a high-energy folded conformation, by the displacement of Y398 from its native position to reduce possible steric clashes and hence expressing very low DS (34.79 kJ/mol), and loss of activity (IC_50_ = 4.41) (Fig. 72b). Similar reasons were reported for the relevant drop in DS, in case of compounds **C86**_**34**_ (IC_50_ = 5.64 μM; DS 56.14 kJ/mol) and **C86**_**33**_ (IC_50_ = 5.77 μM; DS 66.87 kJ/mol) having cyclic substituents at position-4 of coumarin nucleus and hence trying for larger room and conformational rearrangements into the MAO-B binding site.

## Coumarin analogues as Amyloid β peptide (Aβ) inhibitors

**Scheme 1:**
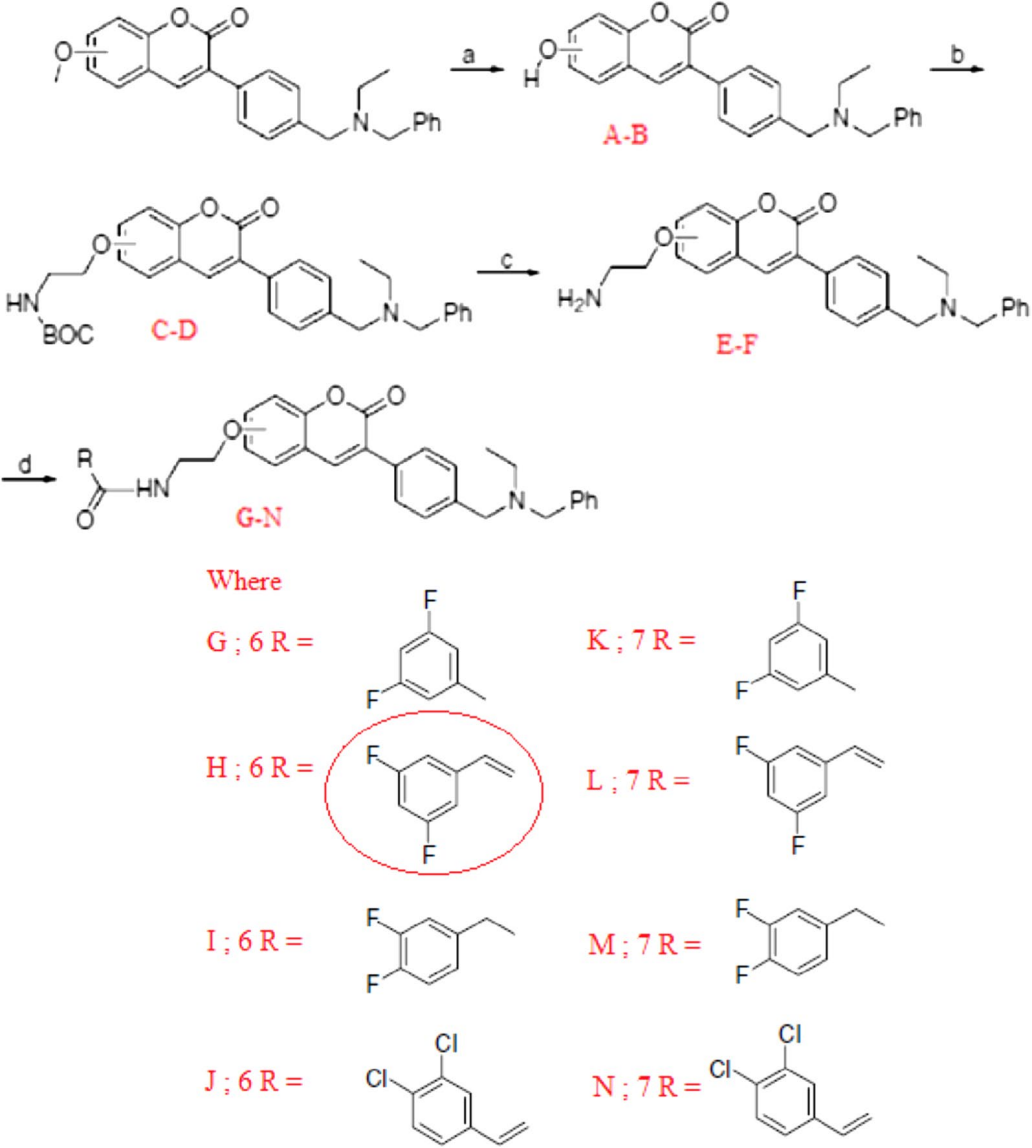
Synthesis of the studied coumarin compounds (**C87**_**G**–**N**_). Reagents: (a) HBr; (b) 2-(Boc-amino)ethyl bromide; (c) CF_3_COOH; (d) DCC, (3,5-difluorophenyl)acetic acid, 3,5-difluorocinnamic acid, 3,4-difluorodihydrocinnamic acid or 3,4-dichlorocinnamic acid [15]

**Fig. 73:**
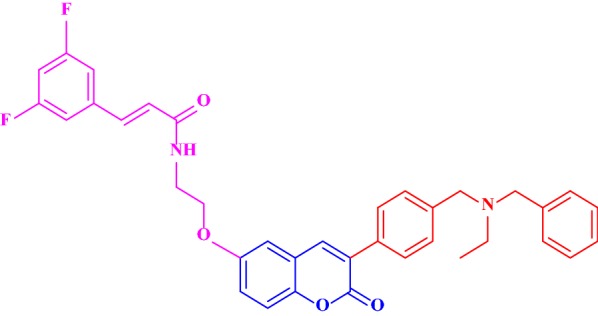
Molecular structure of compound **C87**_**H**_

De novo, coumarins are known for their bioactivity profile. Synthetic as well naturally occurring coumarin derivatives display a wide range of biological properties such as anti HIV [99], antitumor [100], anti-cytotoxicity [101], anti-inflammatory [102] and anti-dengue [103] to mention a few. Therefore, in order to develop the multifunctional compounds, to combat cause and symptoms of neurodegeneration, Piazzi et al. introduced halophenylalkylamidic substituents at position 6 and 7 of the coumarin moiety as dihalophenyl acid that have been repeatedly reported as BACE1 inhibitors [15]. Inhibitors of BACE1 (β-secretase) have a great potential to be developed into anti-dementia drugs as it is believed that they help in lowering β-amyloid accumulation as reported by Jin et al. [26]. Piazzi et al. synthesized these derivatives (**C87**_**G**–**N**_) via parallel synthesis procedure, in 2 series of 4 distinct reactors (Scheme 1). The inhibitory activity of the synthesized compound was first studied against hAChE (Human Acetylcholinesterase) using Ellman’s method to determine the rate of hydrolysis of acetylcholine and secondly against BACE1, using spectrofluorometric method. Out of all the derivatives synthesized, compound *N*-(2-((3-(4-((benzyl(ethyl)amino)methyl)phenyl)-2-oxo-2H-chromen-6-yl)oxy)ethyl)-3-(3,5-difluorophenyl) acryl amide (**C87**_**H**_) showed the most potential as BACE1 inhibitor with IC_50_ value 0.099 μM (Fig. 73).

**Fig. 74:**
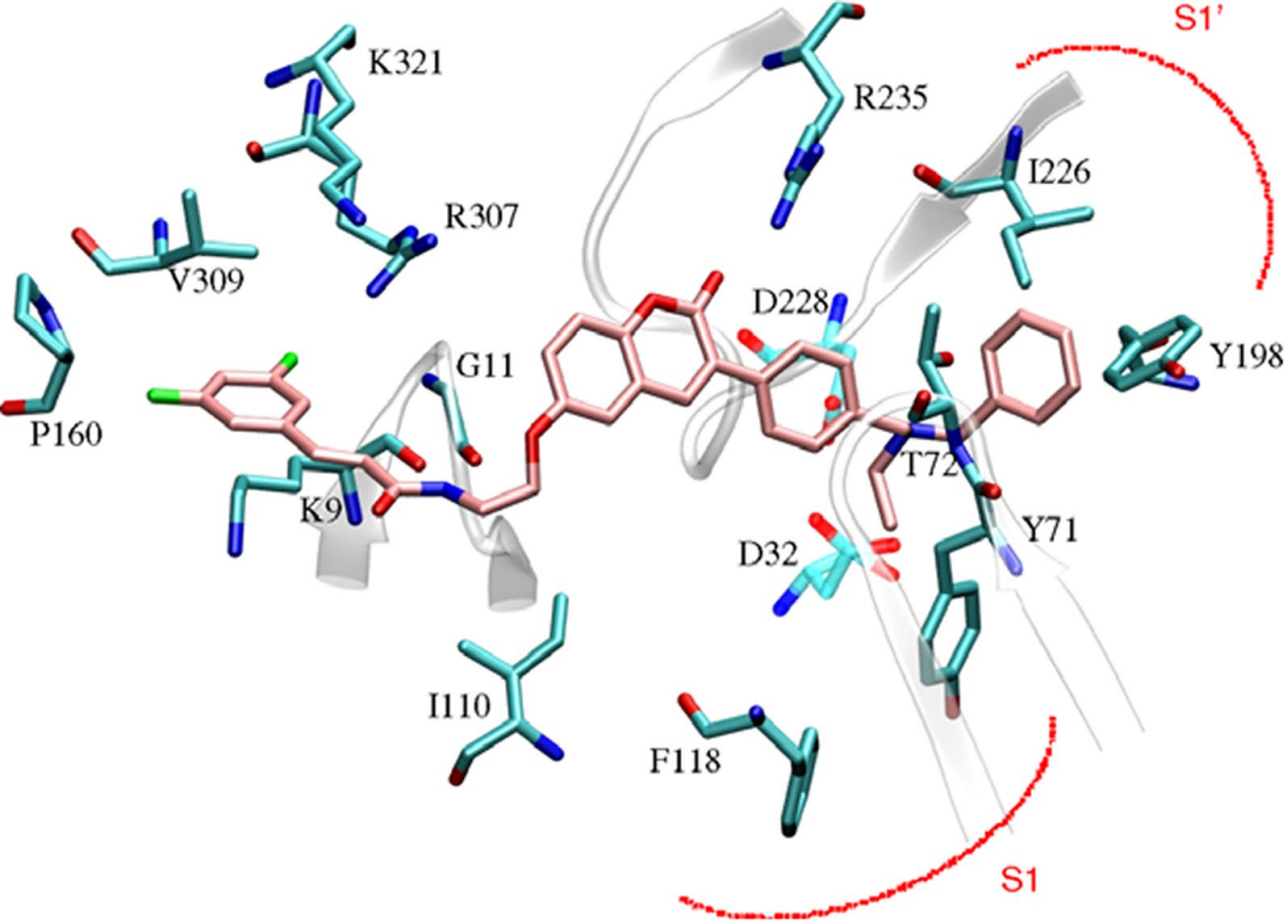
Proposed binding mode of compound **C87**_**H**_ docked at the active site of BACE1 [15]

Compound **C87**_**H**_, was then further docked to the active site of BACE1. The result obtained showed that the compound gave heterogeneous docking poses due to the flexibility of the substituent at position 6 of the coumarin moiety. *N*-Benzyl group were found to interact with Tyr198 and Ile226, whereas the *N*-ethyl portion lied on the aromatic ring of Tyr71. The coumarin core that has 3-phenyl substituent was embedded into the S2 pocket of the enzyme with the carbonyl group being able to form hydrogen bond with the side chain of Arg235 (Fig. 74). Piazzi et al. [15] stated that the flexibility of the bridging amino ethoxy segment and relative structural permittivity of the enzyme portion towards the presence of a substituent at position 6 or 7 of the coumarin nucleus contributed to the relative binding affinity of this series of molecules. The orientation of the substituents at position 6 might be stabilized by the interaction of the amidic nitrogen with Gly11 whereas the 3,5-difluorophenyl portion may possibly be accommodated into a peripheral cationic spot formed by residues Lys9, Arg307, Lys321, Pro160 and Val309 [15].

**Fig. 75:**
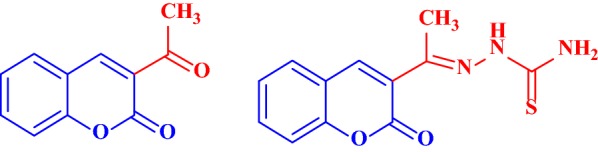
Molecular structure of compound **C88a** and **C88b**

Recently, Ranade et al. [104] studied coumarin thiosemicarbazone (**C88b**) as inhibitors of amyloid β peptide (Aβ) aggregation, as accumulation of Aβ peptide plays an important role in the progression of AD. They used different methods to study the inhibition of Aβ peptide for instance, ThT fluorescence assay, turbidity assay, 1-anilinonaphthalene-8-sulfonic acid (ANS) fluorescence assay and native gel electrophoresis. Interestingly the results from ThT fluorescence assay gave the insight that coumarin thiosemicarbazone has more ability to inhibit aggregation of Aβ peptide as compared to 3-acetyl coumarin (**C88a**) while the turbidity assay gave the information about increase in aggregation of Aβ peptide during initial stages and significant decrease in aggregation lately, for both the coumarin types. Therefore, from both these assays it was concluded that coumarin thiosemicarbazone inhibits the aggregation of Aβ peptide. Assay performed by using the dye 1-anilinonaphthalene-8-sulfonic acid showed that coumarin thiosemicarbazone interacts with hydrophobic regions of Aβ peptide and reduces its aggregation as compared to acetyl coumarin. Results obtained from the native gel assay revealed the ability of coumarin thiosemicarbazone to diminish the formation of high molecular weight aggregates. Hence, the overall result obtained was in favour of coumarin thiosemicarbazone rather than acetyl coumarin and it was concluded from the study that thiosemicarbazone derivatives of 3-acetyl coumarins could play immense role as promising drug molecules for the treatment of AD (Fig. 75) [104].

**Fig. 76:**
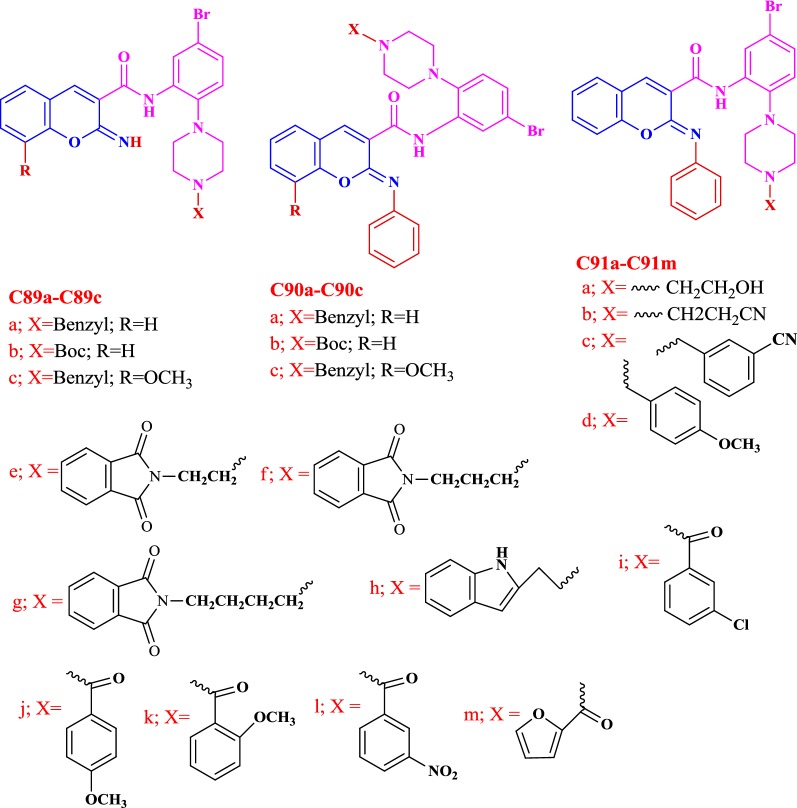
Molecular structure of compounds **C89a**–**c** and **C90a**–**c** and **C91a**–**m**

**Fig. 77:**
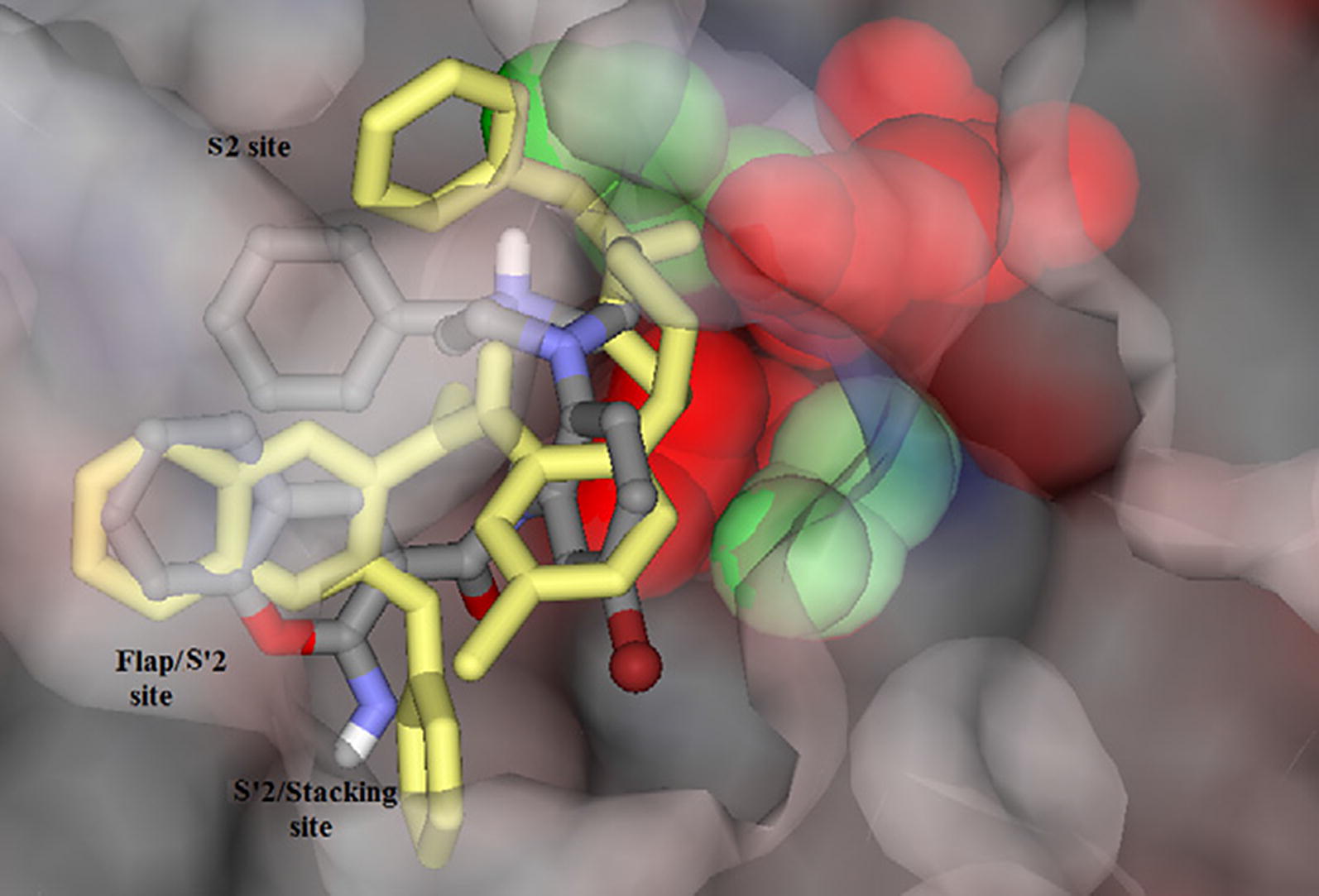
The comparative binding affinities of benzylpiperazine derivative imino-2H-chromene-3-carboxamide **C89a** and its Phenylimino-2H-chromen-3-carboxamide analogue **C90a** [105]

**Fig. 78:**
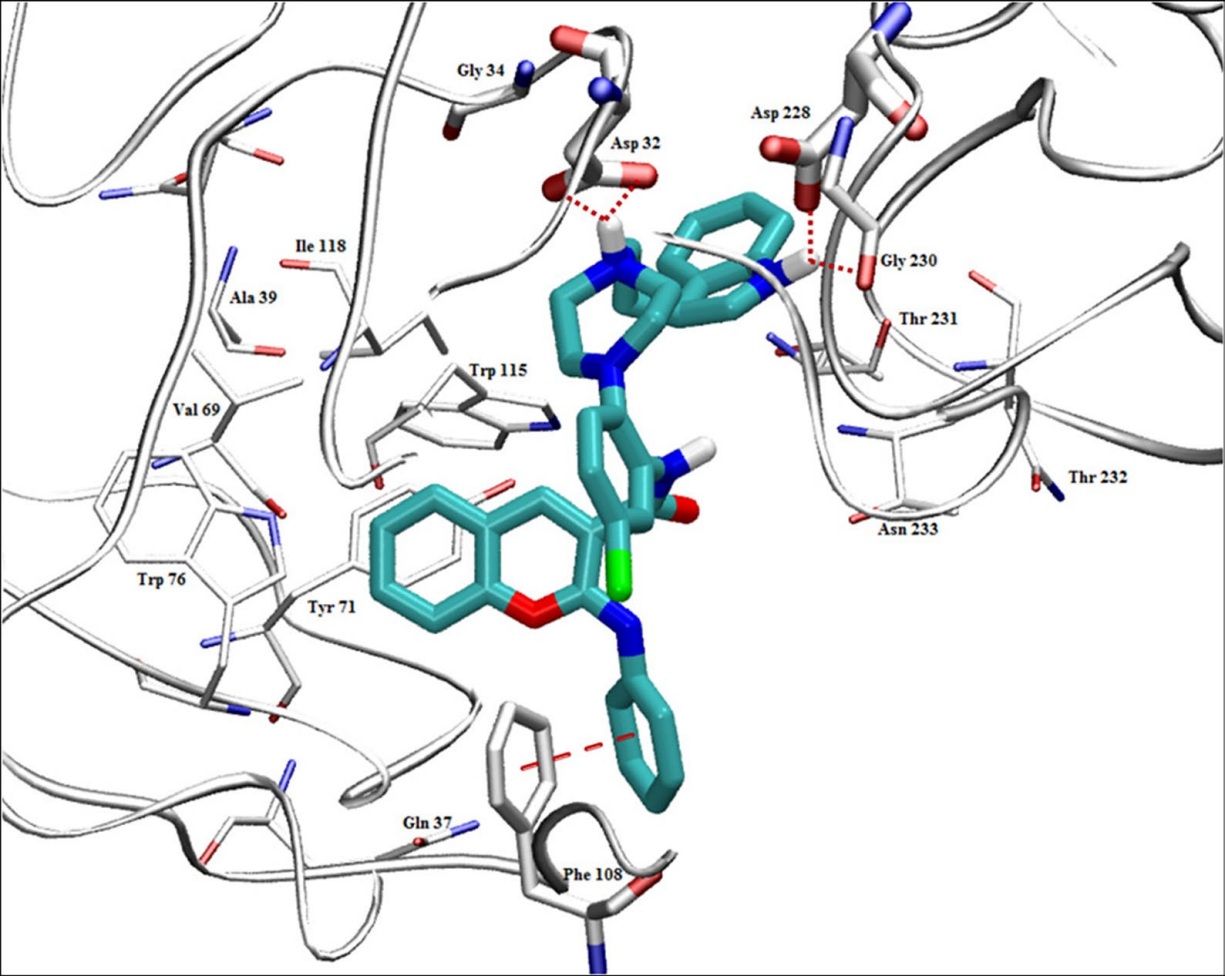
Binding mode of **C91h** into active site of BACE1 [105]

Edraki et al. [105] synthesized a series of phenylimino-2H-chromen-3-carboxamide derivatives **C89a**–**C89c**. **C90a**–**C90c** and **C91a**–**C91m**, as BACE1 inhibitors, which plays an active role in dissolution of aggregated β-amyloid, a peptide that is potentially responsible for enhancing the symptoms of AD (Fig. 76). They evaluated these derivatives for BACE1 inhibitory activity using a FRET-based assay. Furthermore docking was performed to get the insight of their binding behaviour. The results revealed that the phenyl-imino group of the scaffold establishes favourable π–π stacking interaction with side chain of Phe108 of flap pocket as for the case **C89b** and **C90b**. Compound **C89b** was close to inactive against BACE1 enzyme (IC_50_ > 10 μM), but on replacing the hydrogen of imino moiety of the imino-2H-chromene ring, with the phenyl moiety, increased the inhibitory potential of compound **C90b** (IC_50_ = 9.61 μM) (Fig. 77). The derivatives in which heteroatomic groups were attached to N4-piperazine moiety via an aliphatic linkage were reported to possess higher BACE1 inhibitory properties, which was due to the interactions with the residues of S1–S′1 sub-pocket. Some other derivatives displayed high inhibitory potential at 5 and 10 μM on the production of Aβ in N2a-APPswe cells. The highest active compound **C91e**, was reported to possess IC_50_ value 98 nM. This significant inhibitory potential was justified on the basis of the additional hydrophobic interaction with the hydrophobic residue of the S2 sub-pocket of the active site and the network of hydrogen bonding interactions between the heteroatoms of phthalimide moiety and corresponding residue of the active site. On the elongation of methylene linker between phthalimide group and piperazine ring in case of compounds **C91f**, (IC_50_ = 0.326 μM) and **C91g** (IC_50_ = 0.854 μM), the inhibitory properties were reported to decrease due to hindrance of hydrogen bonding interactions at S2 sub-pocket of the active site. Moreover, it was found that introducing 1H-indol ring into N4-piperazine of the scaffold via an ethyl linker increases the inhibition of BACE1 as observed for compound **C91h** (IC_50_ = 0.928 μM) (Fig. 78). More comparisons were made to understand the inhibitory behaviour of other compounds. For instance, comparison of compounds **C89a** and **C90a** demonstrated that the introduction of phenyl moiety into the imino group of benzyl piperazine derivative of imino-2H-chromene-3-carboxamide scaffold **C89a** (IC_50_ = 1.216 μM), resulted in enhanced inhibitory potential of compound in phenylimino-2H-chromen-3-carboxamide derivative **C90a** (IC_50_ = 0.503 μM) [105].

## Coumarin analogues as multifunctional inhibitors

**Fig. 79:**
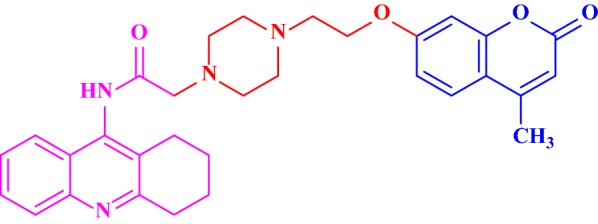
Molecular structure of compound **C92**

Xie et al. [9] designed a series of novel tacrine-coumarin hybrids as multifunctional ChE inhibitors for AD treatment. They connected tacrine to coumarin scaffold through a suitable linker. Tacrine are known as the first ChE inhibitor for AD treatment, before FDA withdrew it, due to its role in severe liver toxicity. Despite its withdrawal, tacrine is still a widely used scaffold in the designing of multifunctional molecules for AD treatment, due to its ability to inhibit ChEs through CAS. Among the tacrine-coumarin hybrids synthesized, compound 2-(4-(2-((4-methyl-2-oxo-2H-chromen-7yl)oxy)ethyl)piperazin-1-yl)-*N*-(1,2,3,4-tetrahydroacri-din-9-yl) acetamide (**C92**) showed the highest inhibitory activity against AChE (Fig. 79) [9].

**Fig. 80:**
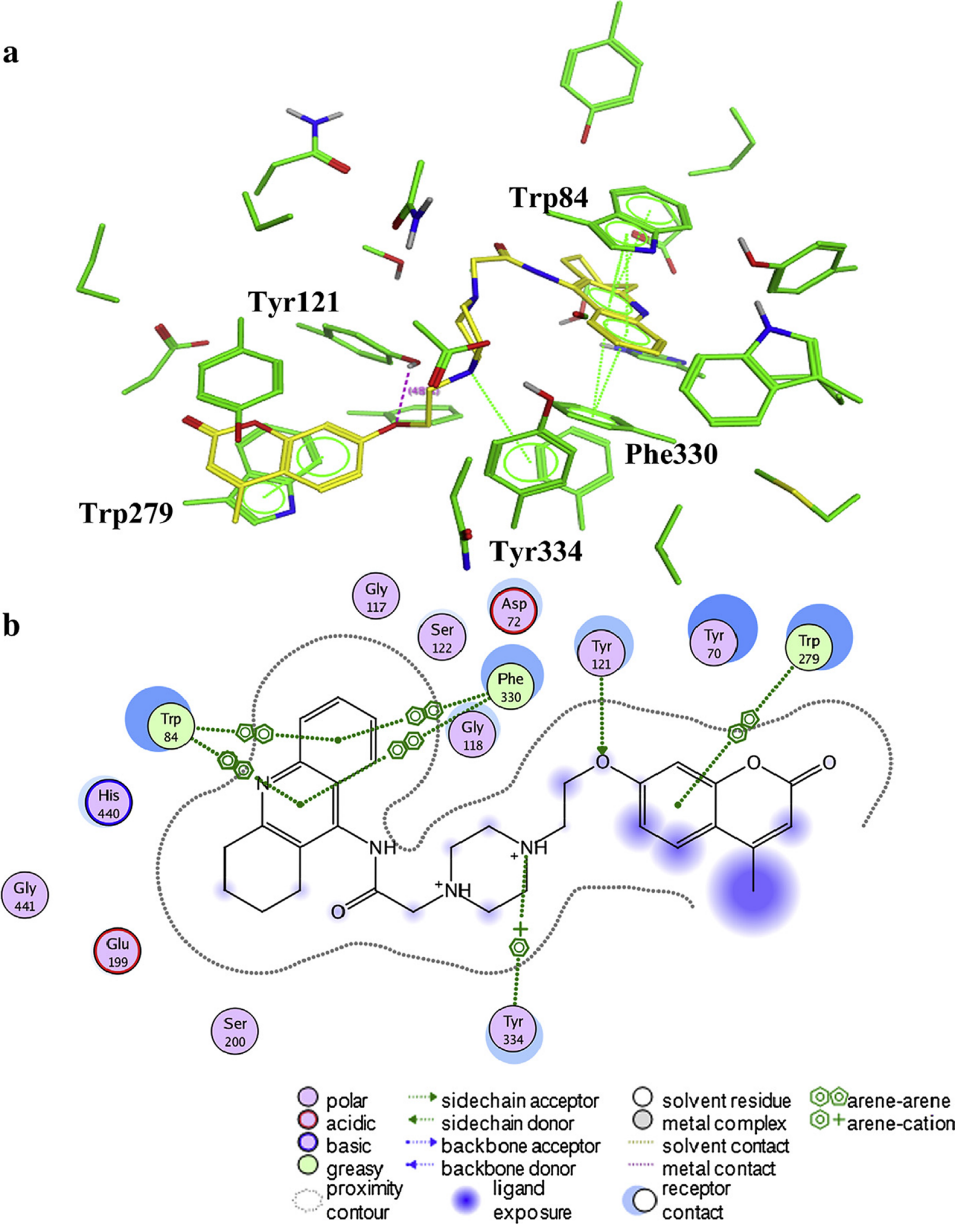
3D docking model of **C92** with TcAChE (**a**, **b**) [9]

Among the factors that contributed to the good AChE inhibitory activity of compound **C92** was the linker length between coumarin moiety and piperazine, and the substituent at position 4 of the coumarin moiety. **C92** with only two-carbon linker provided a good AChE inhibitory activity compared to other hybrids with linker length more than two carbons. The compounds, which possessed methyl substituent at position 4 of coumarin moiety, showed better AChE inhibitory activity compared to the compounds, which has H, methoxy and phenyl group as substituent. The molecular modelling results for **C92** showed, that the tacrine moiety of **C92** was bound to the CAS of AChE and stacked against the phenyl ring of Phe330 and the indole ring of Trp84, whereas the coumarin ring interacted with the indole ring of Trp279 of PAS via π–π stacking interactions (Fig. 80). As expected to be, the piperazine was found to bind to the middle gorge site of AChE through a cation-π interaction, between its protonated nitrogen atom and Tyr334, enhanced by a hydrogen bond, that was formed between the oxygen atom of **C92** side chain and Tyr121. Besides a favourable AChE inhibitory result, compound **C92** also showed promising metal chelating property and low cell toxicity. The multifunctional ability of **C92** makes it to be considered as a promising lead compound for further research and its derivatization in the future.

**Fig. 81:**
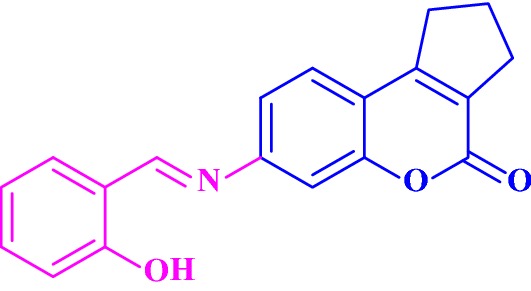
Molecular structure of compound **C93**

Huang et al. [12] designed new multifunctional molecules endowed with the hope of developing novel multifunctional drug that are able to improve clinical symptoms as well as diminish the progression of diseases. They combined coumarin and the pharmacophore moiety of clioquinol (CQ), a renowned metal chelator. Various literatures have reported coumarin analogues as potent MAO-B inhibitors and Aβ anti-aggregation, whereas CQ has been reported to help in decreasing the rate of cognitive decline in moderately severe AD patients in a phase II clinical trial. This integration was made to obtain a novel series of coumarin derivatives that were expected to be biometal chelators, as well as inhibitors of MAO-B and Aβ aggregation. The inhibitory activities of the synthesized derivatives against hMAO-B was identified by measuring their effects on the production of H_2_O_2_ from p-tyramine with pargyline and iproniazid as references. Among the derivatives tested, compound 7-((2-hydroxybenzylidene)amino)-1,3,3a,9b-tetrahydrocyclopenta[c] chromen-4(2H)-one (**C93**) gave the most promising result as selective hMAO-B inhibitor when compared to the references pargyline and iproniazid (Fig. 81) [12].

**Fig. 82:**
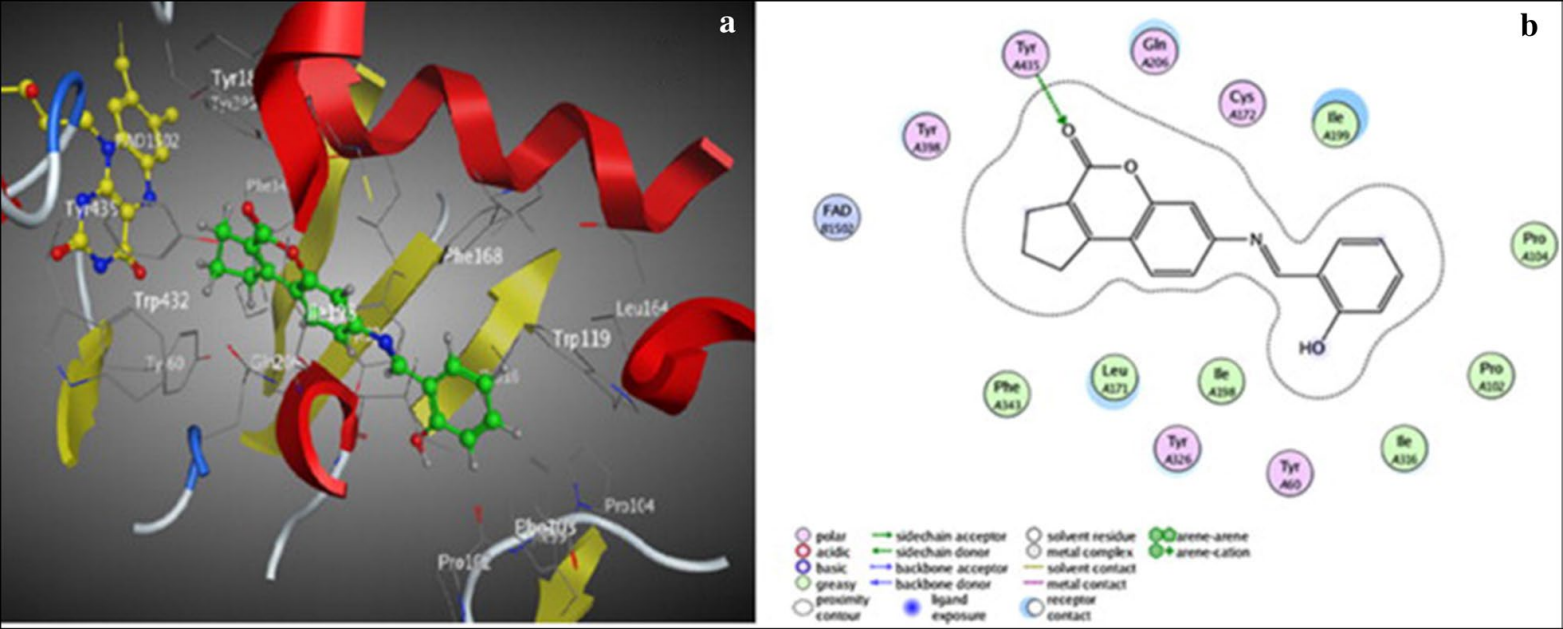
Molecular docking of **C93** with MAO-B (**a**, **b**) [12]

Huang et al. [12] therefore stated, that the steric effect at positions 3 and 4 might have been benefited to hMAO-B inhibitory activity. The result showed by compound **C93** which has cyclopentenyl substituent at the 3 and 4 positions of compound, strongly supported their hypothesis. The carbon nitrogen double bond might also have been played a part in improving its MAO-B inhibitory activity. There was a significant decrease in hMAO-B inhibitory activities, when the carbon nitrogen double bond was hydrogenated. Huang et al. justified this by stating that the carbon nitrogen double bond has influence on the hMAO-B inhibitory activity to different degrees. When tested for its metal-chelating effect, the result showed that there was an interaction between Cu^2+^ and compound **C93** due to the increase of Cu^2+^ concentration. Based on this observation, Huang et al. concluded that the compound might have effectively chelated Cu^2+^ and have served as metal chelators in AD treatment. Docking studies were performed on compound **C93**, to identify its interaction mode with MAO-B. The result obtained showed that the coumarin moiety was located within the substrate cavity of the enzyme and a hydrogen bond was observed with Tyr435 (Fig. 82). The substituent of compound **C93** at position 7, was directed towards the hydrophobic pocket in the entrance cavity, establishing hydrophobic interactions with residues Phe103, Pro104, Trp119, Leu164, Leu167, Phe168, Ile199 and Ile316. Huang et al. stated that the interaction made by this compound with the enzyme is the best explanation for its good hMAO-B inhibitory activity.

**Fig. 83:**
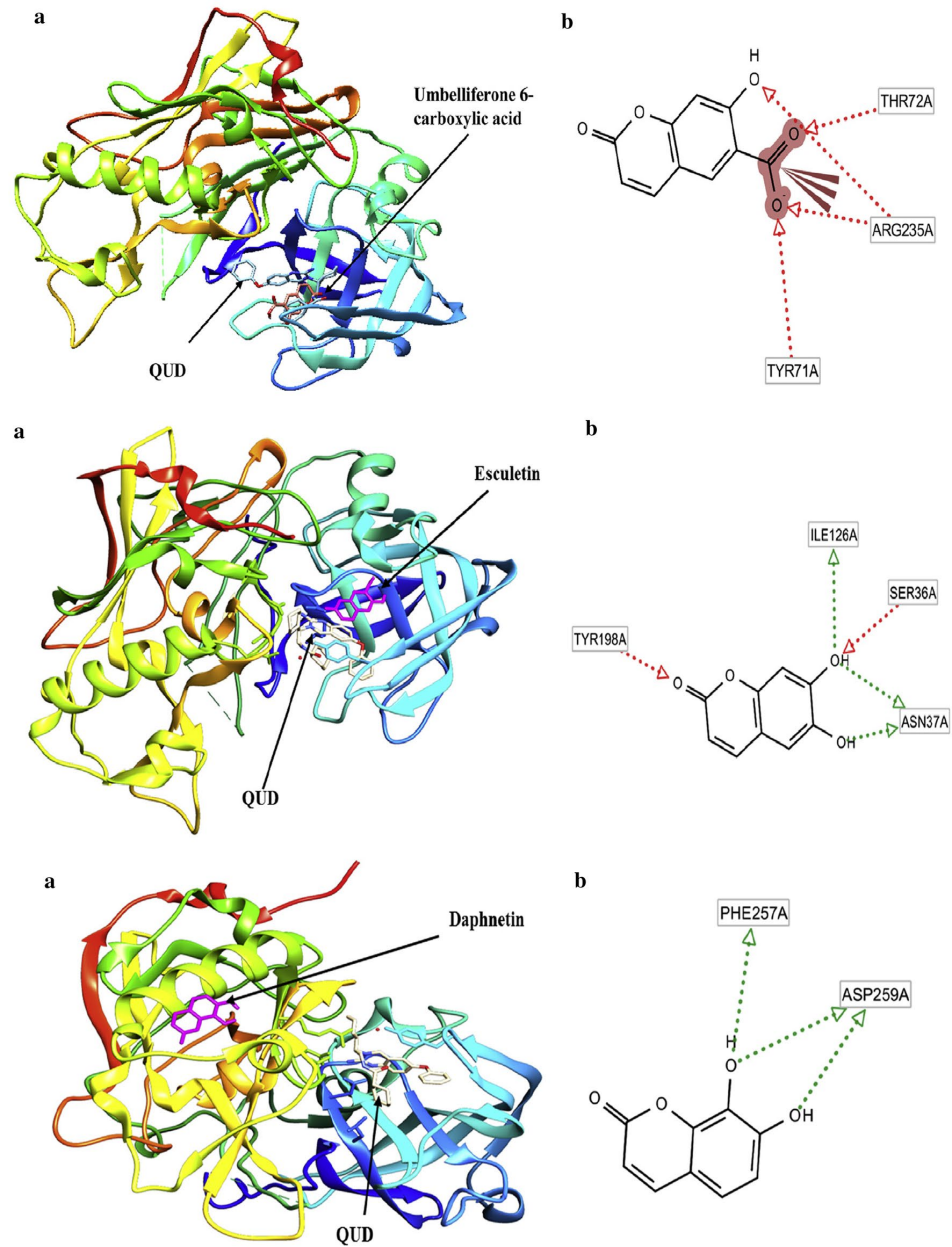
**a** Molecular docking model BACE1 with **C94b**, **C94h** and **C94j**: (**b**) Diagram of the ligand interaction of **C94b**, **C94** **h** and **C94j** in the active site of BACE1 [106]

It is of great importance that natural as well as synthetic coumarins, both display large range of pharmacological properties and hence they own a special place in the field of pharmaceutical research. In this respect Ali et al. isolated umbelliferone (**C94a**), umbelliferone 6-carboxylic acid (**C94b**), scopoletin (**C94c**), isoscopoletin (**C94d**), 7-methoxy coumarin (**C94e**), scoparone (**C94f)**, scopolin (**C94g**), esculetin (**C94h**) and 2′-isopropyl psoralene (**C94i**) from Angelica decursiva and evaluated their inhibitory effects against acetylcholinesterase (AChE), butyrylcholinesterase (BuChE), and β-site amyloid precursor protein cleaving enzyme 1 (BACE1) enzyme activity. 2′-Isopropyl psoralene was isolated for the first time from Angelica decursiva. The results showed that among all the coumarins esculetin and daphnetin (**C94j**) exhibited good AChE inhibitory activity with IC_50_ values of 6.13 and 11.57 μM against berberine (IC_50_ = 0.717 μM), whereas daphnetin, esculetin, and umbelliferone 6-carboxylic acid exhibited highest BuChE inhibitory activity with IC_50_ values of 8.66, 9.29, and 27.19 μM against berberine (IC_50_ = 7.01 μM). Both, esculetin and daphnetin have a catechol group which was noted to be significant for enhancing activity against AChE and BuChE and the presence of sugar moiety (umbelliferone 6-carboxylic acid, umbelliferone, scopoletin, isoscopoletin, 20-isopropylpsoralene, IC_50_ values 104.12, 145.19, 150.28, 153.77 and 173.89 μM, respectively) as well methoxylation (7-methoxy coumarin IC_50_ = 186.47 μM) were the main cause to diminish the activity. Finally, umbelliferone 6-carboxylic acid, esculetin and daphnetin exhibited promising BACE1 inhibitory activity with IC_50_ values of 0.34, 7.67, and 11.19 μM against quercetin (IC_50_ = 13.98 μM). These coumarins have carboxyl or catechol groups, which were reported to effectively enhance the anti-AD properties. They scrutinized the potentials of these three coumarins as BACE1 against the most potent nonpeptic BACE1 inhibitor reported to date according to protein data bank which is 2-amino-3-{(1r)-1-cyclohexyl-2-[(cyclohexylcarbonyl)amino]ethyl}-6-phenoxyquinazolin-3-Ium (QUD). They generated a 3D structure of BACE1 by using Autodock 4.2. to perform the molecular docking studies. They found that the corresponding ligand interactions of umbelliferone 6-carboxylic acid in the active site of BACE1 was encouraged by hydrogen-bonding interrelationship between the Arg235 residue and one carboxylic group interrelation between each of the Tyr71, Arg235 and Thr72 residues with one hydroxyl group at C-7 and one carboxylic group at C-6. Similarly, comparable ligand interaction for esculetin in the active site of BACE1, consisted of two hydrogen-bonding interactions with the Asn37, Ser36, and Ile126 residues of BACE1 and two hydroxyl groups of the compound, while binding of daphnetin in the active site of BACE1 was arbitrated by two hydrogen-bonding interrelations with Asp259 and Phe257 residues of the enzyme and two hydroxyl groups of the compound (Fig. 83). The binding energies of these three coumarin were − 4.58, − 6.25 and − 6.37 kcal/mol, which was comparable to that of QUD (− 10.99 kcal/mol). Their study concluded that additional hydrogen bonding and carboxylic groups could help in stabilizing the open form of BACE1 and could help in tighter binding to the active site, hence resulting in more effective enzyme inhibition [106].

**Fig. 84:**
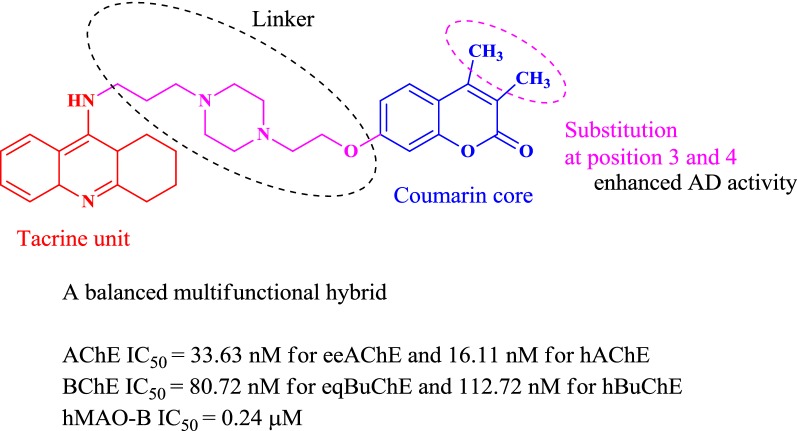
Molecular structure of compound **C95**

**Fig. 85:**
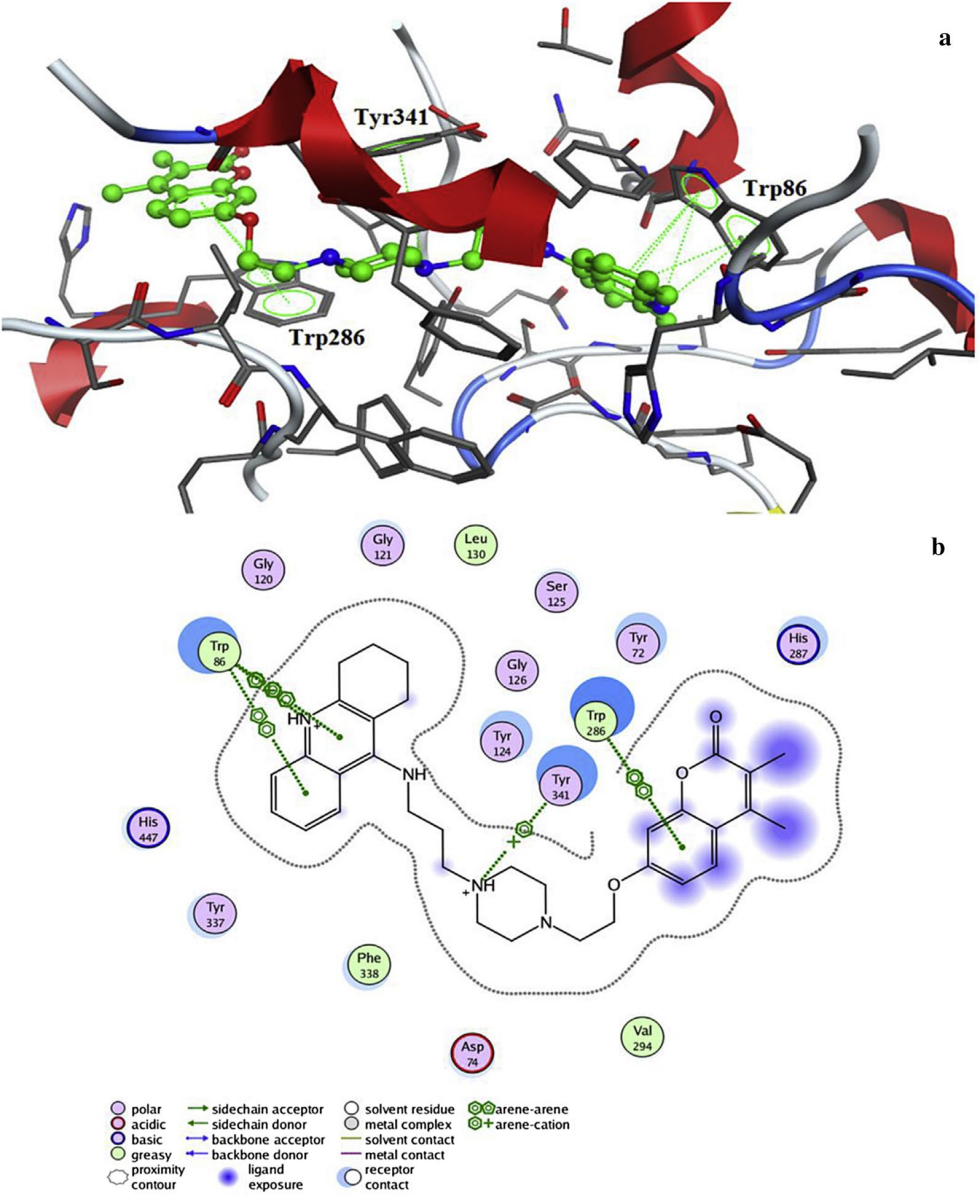
**a** 3D docking model of compound **C95** with AChE. **b** 2D schematic diagram of docking model of compound **C95** with AChE [107]

**Fig. 86:**
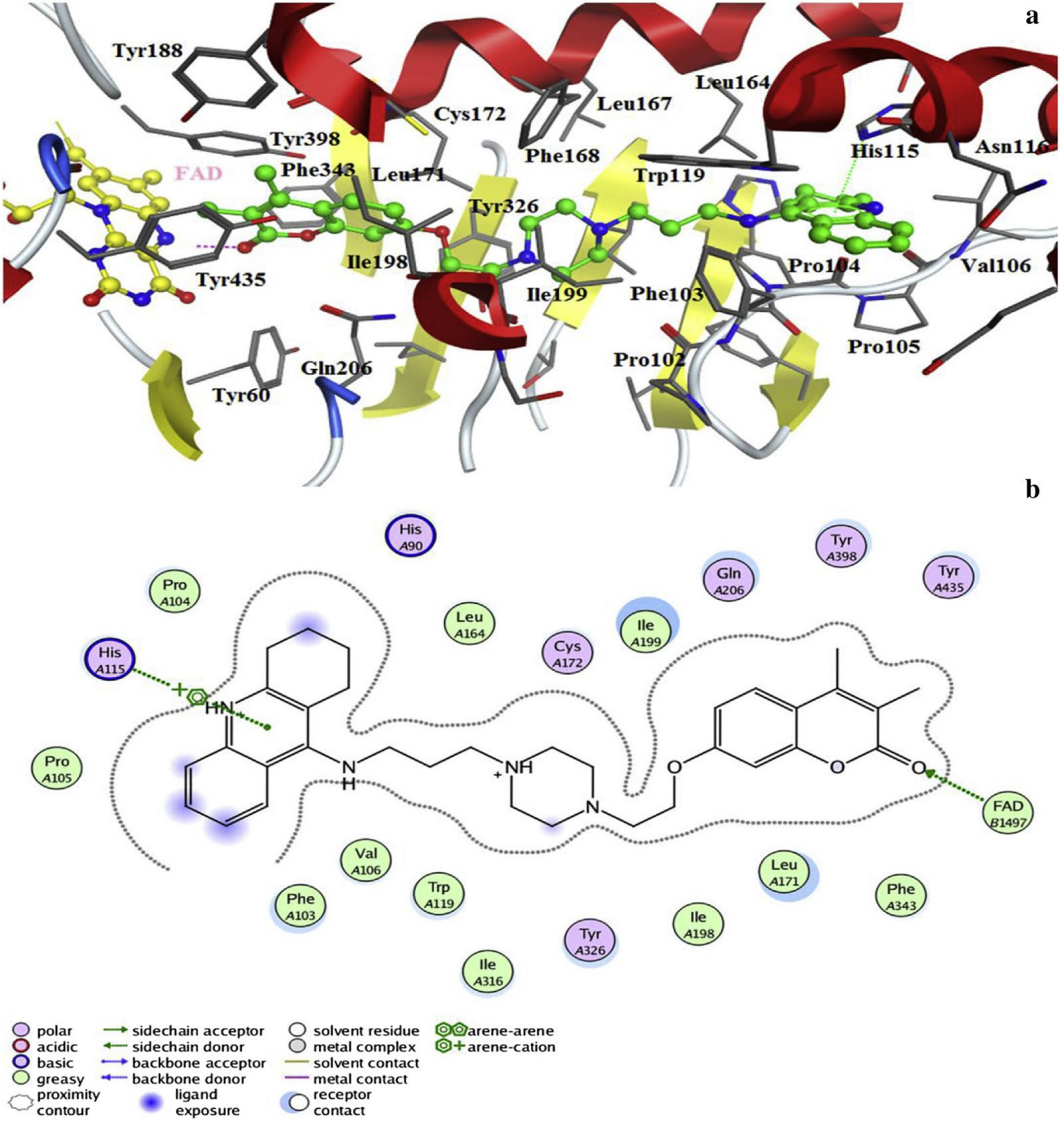
(**a**) 3D docking model of compound **C95** with MAO-B. **b** 2D schematic diagram of docking model of compound **C95** with MAO-B [107]

In continuation to their previous work based on the synthesis of multi-target inhibitors of AD, Xie et al. [107] synthesized another series of multifunctional tacrine-coumarin hybrids and evaluated their performance as ChEs, MAOs and the ability to penetrate the blood–brain barrier (BBB). Most of the compounds displayed potent inhibitory activity towards AChE, BuChE but selective inhibition against MAO-B. The length of the linker bridging the coumarin nucleus and the tacrine unit was found to enhance the AChE activity whereas it didn’t influenced much to the inhibition of BuChE and MAO-B. Among all the compounds, compound **C95** was the most promising compound displaying highest inhibition for MAO-B, eelAChE, eqBuChE, hAChE as well as hBuChE and reported as the ‘mixed type multifunctional strong inhibitor’ for AD (Fig. 84). Its docking results revealed its simultaneous binding to CAS, PAS, mid-gorge site of AChE and covering the substrate and entrance cavities of MAO-B. Moreover, it also showed good penetration ability for the BBB, regardless of the possible interaction with carriers or efflux transport proteins (Figs. 85 and 86) [107].

**Fig. 87:**
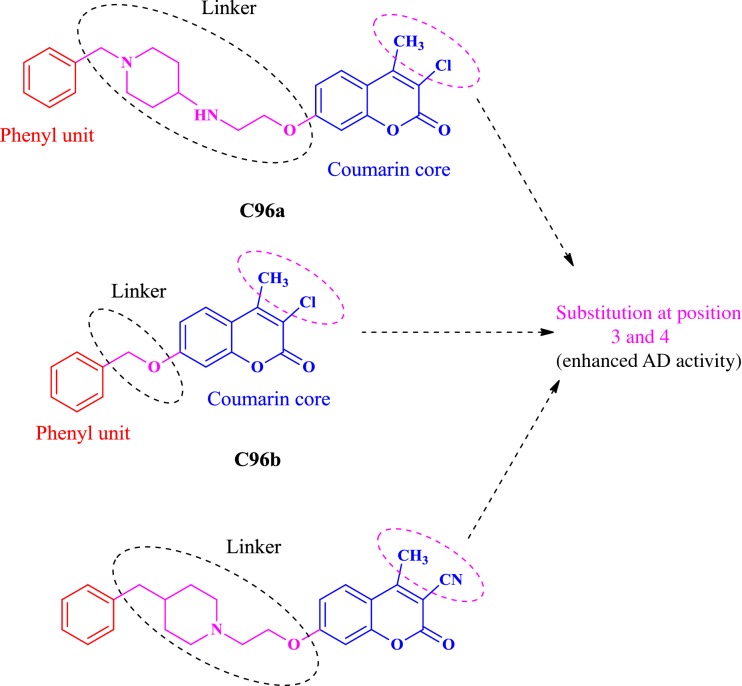
Molecular structure of compounds **C96a**–**C96c**

**Fig. 88:**
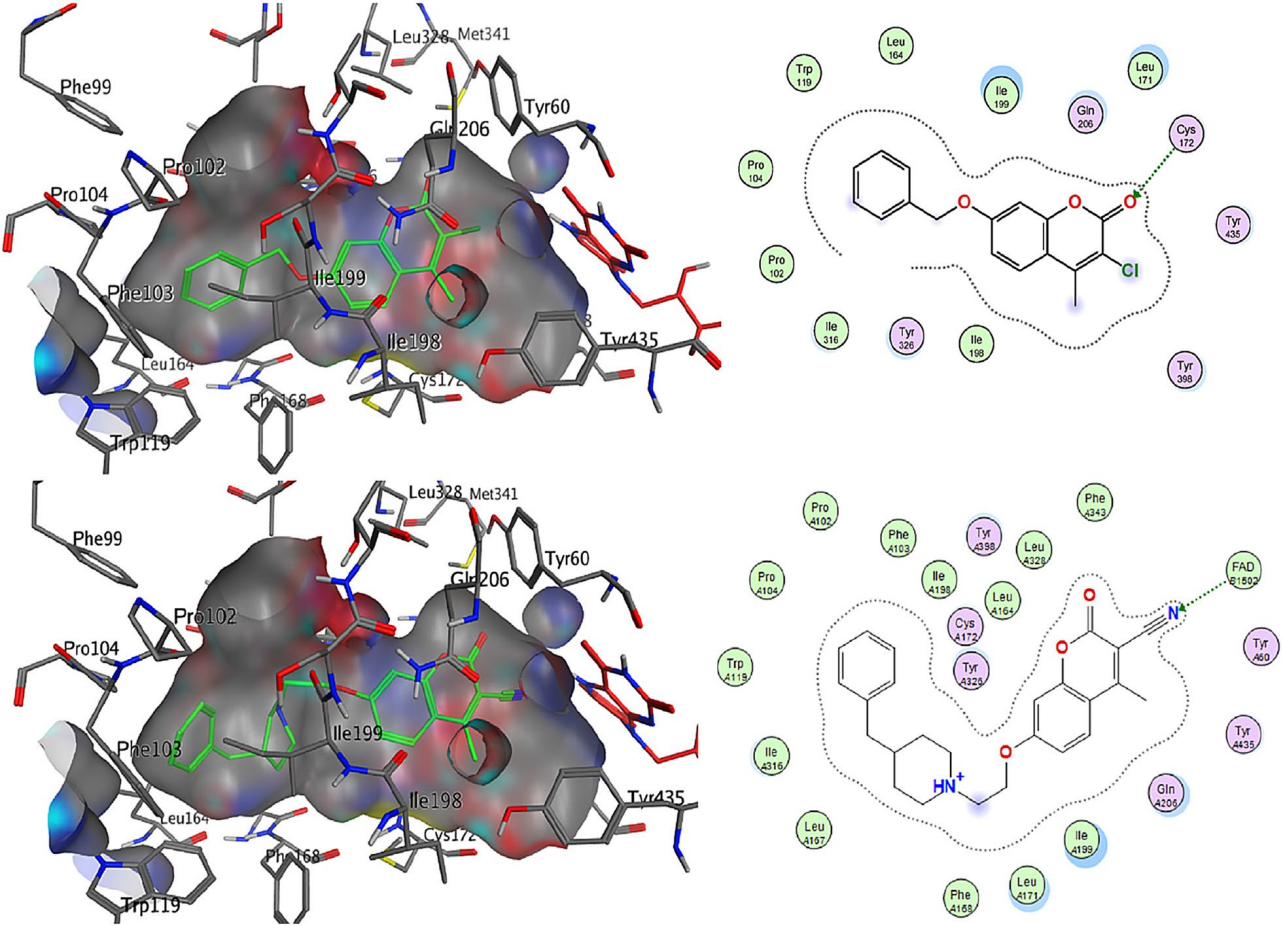
The hMAO-B active site cavity (left) and interaction maps (right) displaying the binding and interactions of compounds **C96b** (top) and **C96c** (bottom) [108]

**Fig. 89:**
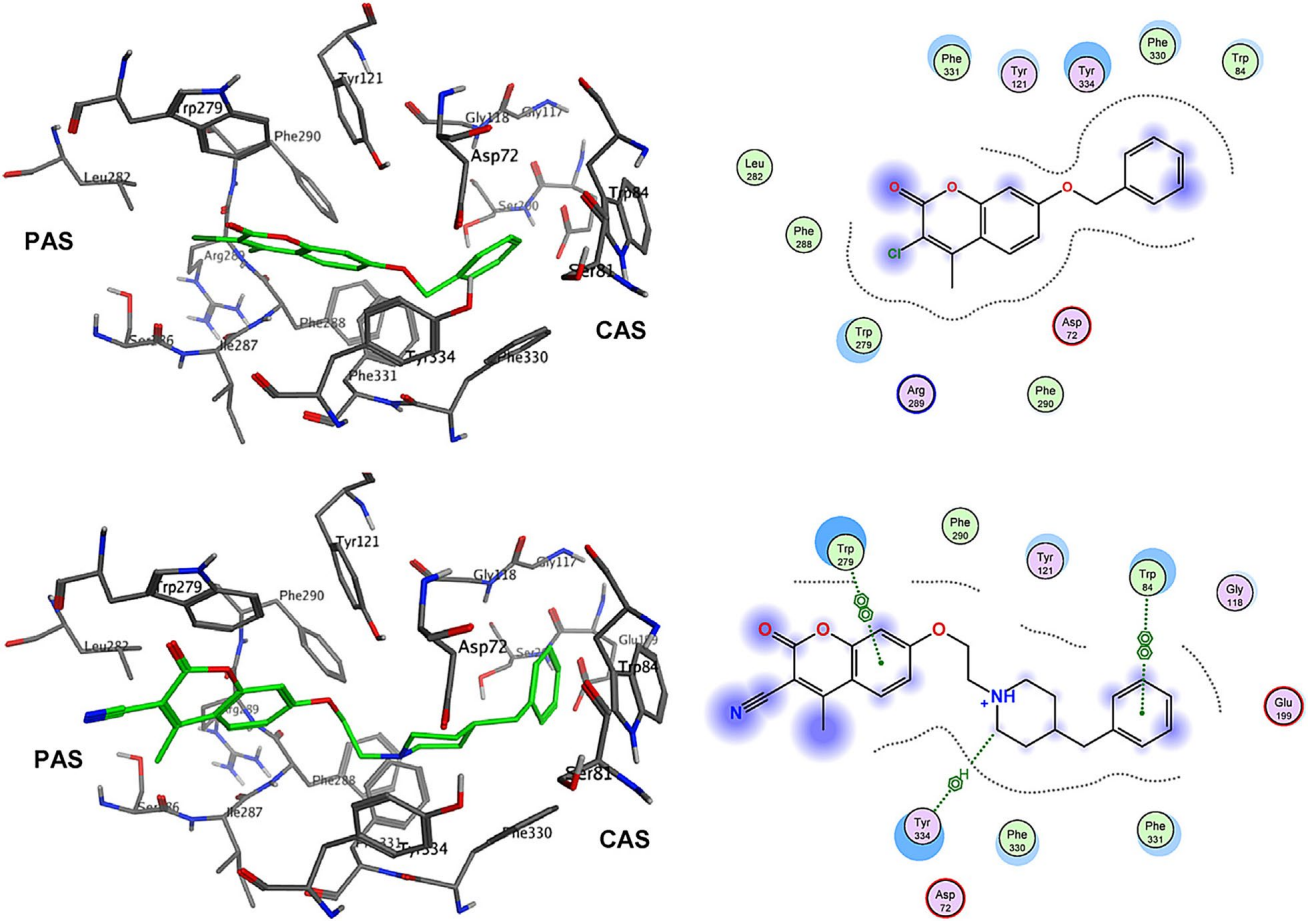
The eelAChE active site cavity (left) and interaction maps (right) displaying the binding and interactions of compounds **C96b** (top) and **C96c** (bottom) [108]

**Fig. 90:**
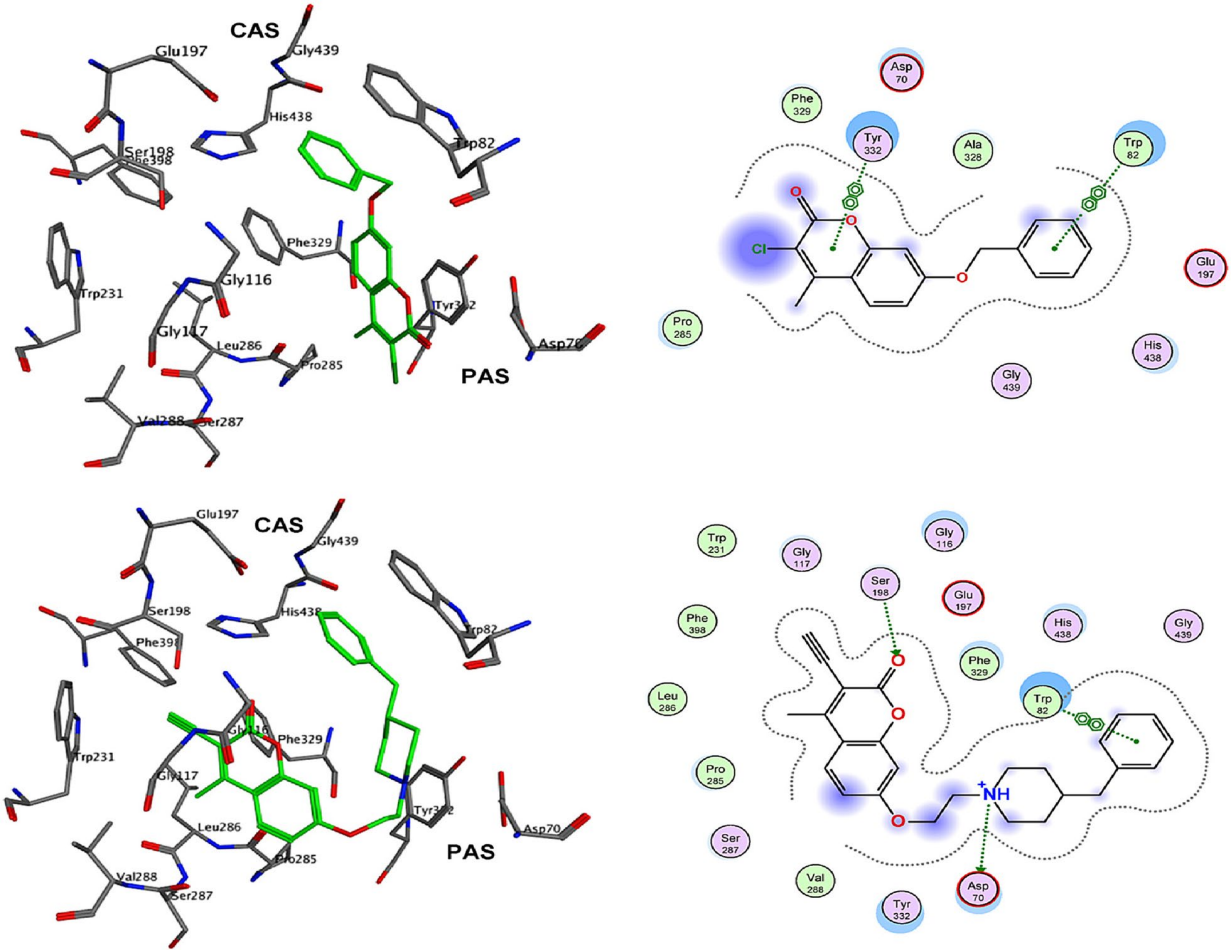
Complex of compound **C96b** (top) and **C96c** (bottom) with the eqBuChE homology model (left) and the interaction maps (right) [108]

Joubert et al. [108] designed a series of 7-substituted coumarin ligands as multi-targeted inhibitors of AD and checked their ChEs bioactivity profile by application of Ellman’s method using donepezil and tacrine as standards against electrophorus electricus enzyme (eelAChE) and equine serum enzyme (eqBuChE). Most of the analogues exhibited potent inhibitory activity with IC_50_ values of micromolar and nanomolar. It is worth to mention that the alkyl ether linker at position-7 greatly influenced the inhibition. Compound **C96a** (IC_50_ = 1.58 μM) displayed highest AChE inhibitory and **C96b** (IC_50_ = 0.96 μM) displayed highest BuChE inhibitory power (Fig. 87). Interestingly all the synthesized compounds displayed considerable and selective MAO-B inhibitory activities when compared to the standards selegiline and clorgyline in the nanomolar range. Overall compound **C96c** was found to be the most important multifunctional ligand displaying balanced bioactivity profile to inhibit ChEs (eelAChE: IC_50_ = 9.10 μM; eqBuChE: IC_50_ = 5.90 μM) and hMAO-B (IC_50_ = 0.30 μM) along with **C96b**. Molecular docking was performed to get the insight of the binding modes and interaction of these two best-ranked compounds. The results obtained suggested that within the active site of MAO-B the coumarin moiety of both the compounds was bounded in the polar region of the substrate cavity, in the close vicinity of the FAD cofactor, and the ‘aromatic sandwich’ was defined by the residues Tyr398 and Tyr435. The carbonyl moiety of **C96b** showed a hydrogen bond interaction with Cys172 and the nitrile on the position-3 of **C96c** exhibited a hydrogen bond interaction with N5 of FAD. Moreover the benzyl- and *N*-benzylpiperidine side chain of **C96b** and **C96c** was found to extend and past the Ile199 which was situated in the entrance cavity of the enzyme. Within the hydrophobic environment of the entrance cavity, the benzyl- and *N*-benzylpiperidine side chain were reported to be stabilized by Van der Waals interactions. The binding orientations and interactions are depicted in Fig. 88. Moreover, the reason behind the selectivity for BuChE over AChE in case of compound **C86b** was reported to be mainly its inability to reach the CAS and having no interactions with its surrounding residues. However, the results obtained for **C96c** showed that the coumarin moiety was bounded to the PAS site of the enzyme, establishing a π–π stacking interaction between its phenyl ring and the indole ring of Trp279. Its piperidine moiety was found to interact with Tyr324 in the mid-gorge and its *N*-benzylpiperidine moiety which was located at the CAS, showed a π–π stacking interaction with Trp84 (Fig. 89). The important docking results of these compounds as a good BuChE inhibitor dipicts that the benzyl- and coumarin moieties were bounded in the CAS and PAS region of the enzyme respectively and hence establishing a π–π stacking interactions with Trp82 and Tyr332. **C96c** was found to be well accommodated within the active site gorge and showed a binding mode with a U-shaped conformation. The lowest energy binding orientation of **C96c** enabled its benzyl moiety to interact with Trp82 in the CAS allowing a π–π stacking interaction (Fig. 90) [108].

## Conclusion

AD remains among one of the serious problem and the most common cause of dementia worldwide. It causes depression, abnormal behaviour, anxiety as well as irreversible decline in cognitive, social and physical function. The present review presents coumarin and its derivatives as an important starting tool in the development of anti-Alzheimer drug discovery process. In search of new anti-Alzheimer drugs, coumarin-bearing derivatives have been synthesized abundantly and screened for their anti-Alzheimer properties. Interestingly, many of these derivatives are found to possess promising activity due to their unique features that render them attractive for the discovery and development of novel anti-Alzheimer drugs.

Coumarins bearing acetamide pendant (**C2**) were reported as potent AChE inhibitors (IC_50_ 1.2 μM) whereas coumarins with phenylpiperazine fragment (**C8**) were also found to be effective AChE inhibitors as they effectively adopt a sandwich structure by entering into the gorge of AChE enzyme, resulting in a parallel π–π stacking. Insertion of aromatic groups at position 7, and the presence of electron withdrawing substituents (for example fluoro) at position 4 in the coumarin core (**C11**) was found to effectively increase their dual ability to inhibit AChE and BuChE. It is worth to mention the position of methoxy group, as it highly influences BuChE/AChE selectivity in 71 versus 9000 ratio if it is inserted at positions 7- (**C13a**) and 8- (**C14a**) in the coumarin nucleus. Nevertheless, 3-acyl chlorides coumarin derivatives, such as **C52** was reported to be active, against both the isoforms of MAO viz. MAO-A and MAO-B and in particular towards MAO-B with IC_50_ value of 8.0. Results has also revealed that if phenyl-imino group is present in the coumarin scaffold then it establishes favourable π–π stacking interaction with the side chain of Phe108 of flap pocket as found for the cases **C89b** and **C90b**. Moreover, it was also reported that tacrine-coumarin hybrids act as multifunctional ChE inhibitors for AD treatment as reported for the compound **C92**.

In conclusion, further modifications in the coumarin nucleus could help in the development of new potential therapeutic agents with improved chemotherapeutic profiles, multifunctional role, reduced toxicity, short therapy time and rapid mechanism of action.

## Abbreviations

AD: Alzheimer’s disease
NFTs: neurofibrillary tangles
Aβ: beta-amyloid
APP: amyloid precursor protein
CSF: cerebrospinal fluid
AChE: acetylcholinesterase
MAO: monoamine oxidase
BuChE: butyrylcholinesterase
hAChE: human acetylcholinesterase
hBuChE: human butyrylcholinesterase
ROS: reactive oxygen species
BACE1: β-secretase
PAS: peripheral anionic site
eelAChE: electrophorus electricus acetylcholinesterase
eqBuChE: horse serum butyrylcholinesterase
TcAChE: *Torpedo californica* acetylcholinesterase
CAS: catalytic active site
ES: ecstatic site
ABS: acyl binding site
CS: catalytic site
FAD: flavin adenine dinucleotide
MPTP: 1-methyl-4-phenyl-1,2,3,6-tetrahydropyridine
MAOIs: MAO-A and MAO-B inhibitors
ANS: 1-anilinonaphthalene-8-sulfonic acid
